# Supplementary material for: Deep-learning-enabled online mass spectrometry of the reaction product of a single catalyst nanoparticle
Source: Nat Commun. 2025 Aug 5;16:7203. doi: 10.1038/s41467-025-62602-3 (PMC12325981; doi:10.1038/s41467-025-62602-3)
Supplement: Supplementary file 1 — Supplementary Information [file 41467_2025_62602_MOESM1_ESM.pdf]

Supplementary information: Deep-learning-enabled online mass spectrometry of the reaction product of a single catalyst nanoparticle

Henrik Klein Moberg, Giuseppe Abbondanza, Ievgen Nedrygailov, David Albinsson, Joachim Fritzsche, Christoph Langhammer*

Department of Physics, Chalmers University of Technology, Göteborg, SE-41296 Sweden.

*Corresponding author(s). E-mail(s): clangham@chalmers.se;

Contents

1 Supplementary Note 1. Catalyst Pre-treatment 3

2 Supplementary Note 2. Catalyst Characterization 4

3 Supplementary Note 3. Raw QMS data for all measurements on n=1000, n=10, n=1 6

4 Supplementary Note 4. Encoder-Decoders for Denoising 9

5 Supplementary Note 5. Comparative Studies of the DAE and other denoising techniques 11

6 Supplementary Note 6. Complete n=1 and n=0 data set of Fig. 5 in main text 19

7 Supplementary Note 7. Synthetic Data Generation & Deep Learning Training 20

8 Supplementary Note 8. Calibration of Resistive Nanofluidic Chip Heaters 26

9 Supplementary Note 9. Ethylene hydrogenation on 1000 Pd nanoparticles 28

10 Supplementary Note 10. Control Experiment: Detection of Pulsed CO_2 in the Absence of Pd Nanoparticles 28

1 Supplementary Note 1. Catalyst Pre-treatment

Prior to nanofluidic chip installation on the experimental setup (Supplementary Figure 1), a temperature stabilization protocol was employed where the conduit connecting the nanofluidic chip outlet to the quadrupole mass spectrometer (QMS) inlet, alongside the primary chamber of the QMS, was heated to 80 °C. This ensured a consistent thermal environment for subsequent measurements. The system was then pumped until the base pressure in the QMS chamber was reduced to the desired level of 10^{-10} mbar, typically over an extended period spanning several days. After the vacuum conditions were established, the sample was introduced, and the QMS components were kept at $T = 35$ °C to eliminate variations due to ambient temperature fluctuations.

The catalyst's activation was conducted through a preconditioning routine involving 20 cycles of alternating carbon monoxide (10% CO in Ar) and oxygen (15% O_2 in Ar) gas pulses, with each pulse lasting for a duration of 15 minutes. This was followed by a complete CO oxidation sequence to activate the catalyst and ensure QMS signal stability. During these procedures, the temperature of the sample was consistently maintained at $T = 280$ °C.

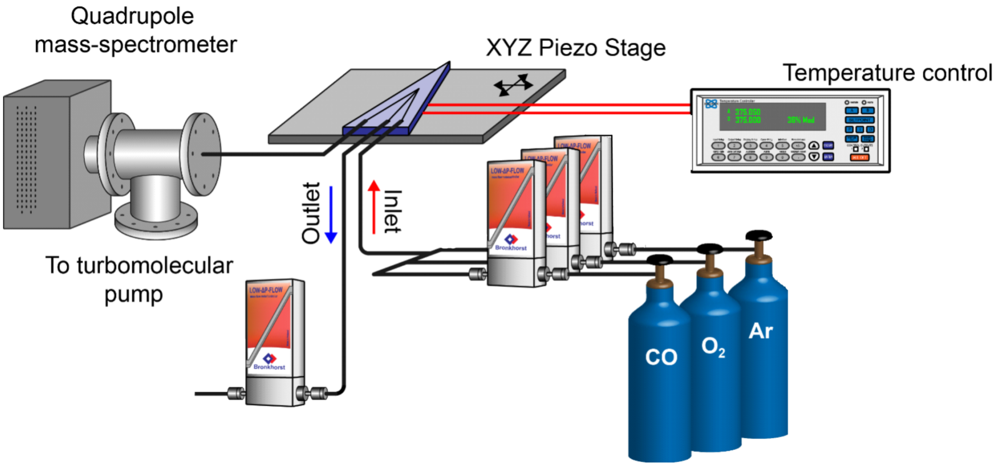

Supplementary Fig. 1 Schematic illustration of the experimental setup. The setup is comprised of a holder for the nanofluidic chip that, on the outlet side, is connected to a quadrupole mass spectrometer (QMS) mounted onto an ultra-high vacuum chamber equipped with both a turbomolecular pump and a rotary pre-vacuum pump. The temperature control unit for the resistive heater fabricated onto the backside of the chip is indicated by the red lines. On the inlet side, the chip is connected to mass flow controllers that enable gas supply at up to 4 bar inlet pressure.

2 Supplementary Note 2. Catalyst Characterization

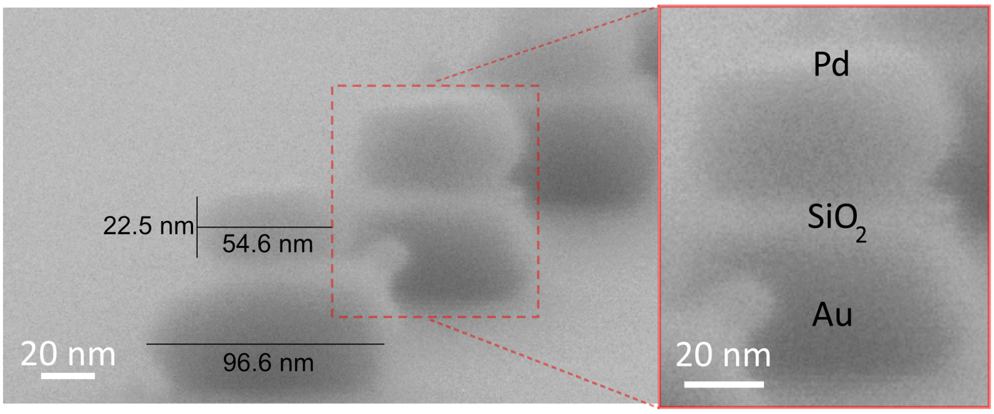

Supplementary Fig. 2 Side-view SEM image of Au–SiO_2–Pd hybrid nanoparticles The nanoparticles considered in this study consist of a plasmonically active but catalytically inert Au base covered by an 8 nm thick SiO_2 spacer layer onto which the Pd catalyst particle is grown via subsequent physical vapor deposition through a nanofabricated lithography mask. Based on the this side-view image, we can estimate the size of the Pd particles, and thus their active surface area, as $A_s = \pi(d/2)^2 + \pi dh = 0.0072 \pm 0.00086\ \mu\text{m}^2 = 7200 \pm 860\ \text{nm}^2$, by taking the average of the three particles in the image.

To get a reliable estimate of the surface area of our Pd catalyst nanoparticles we used side-view scanning electron microscopy of the Au–SiO_2–Pd hybrid structures applied (Supplementary Figure 2). This analysis reveals that the Pd particles on the top of these structures on average have a diameter $d = 59.4 \pm 3.7$ nm and thickness $h = 23.5 \pm 1.6$ nm. This, in turn results in an estimated average surface area for the Pd nanoparticles as $A_s = \pi(d/2)^2 + \pi dh = 0.0072 \pm 0.00086\ \mu\text{m}^2 = 7200 \pm 860\ \text{nm}^2$. We also note, that the size of the Pd particles is smaller than the size of the plasmonic Au particles underneath. This is the result of a gradual closing of the lithography mask opening during the subsequent physical vapour deposition of the Au, SiO_2 and Pd layers.

Supplementary Figure 3 presents a zoomed-out top-view of the $N = 1000$ Pd chip, where the two bright horizontal bands mark the channel walls and the intervening field contains the full grid of $N = 1000$ individual Pd nanoparticles. Each bright dot in the array corresponds to a single Pd catalyst particle, demonstrating uniform size, spacing, and coverage across the channel. This large-area SEM overview verifies the precise control over nanoparticle templating and underpins the quantitative comparison of catalytic activity between the many-particle ($n = 1000$), few-particle ($n = 10$), and single-particle ($n = 1$) regimes.

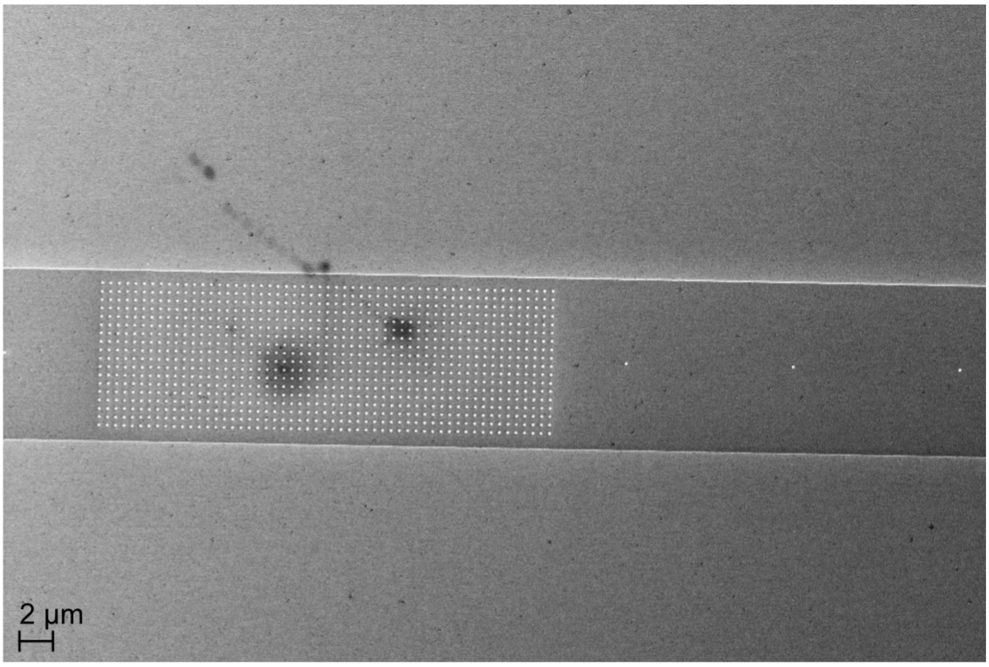

Supplementary Fig. 3 Zoomed-out SEM of a chip showing the full array of $N = 1000$ Pd nanoparticles. Top-view SEM micrograph of the nanofluidic channel region (bounded by the two bright horizontal lines) displaying the complete array of 1000 Pd nanoparticles (nominal diameter ~ 60 nm, center-to-center pitch ~ 270 nm) grown through the lithographic mask. The uniform particle placement across the entire channel area confirms the successful templating of Pd via physical-vapor deposition. Scale bar: 2 μm.

3 Supplementary Note 3. Raw QMS data for all measurements on n=1000, n=10, n=1

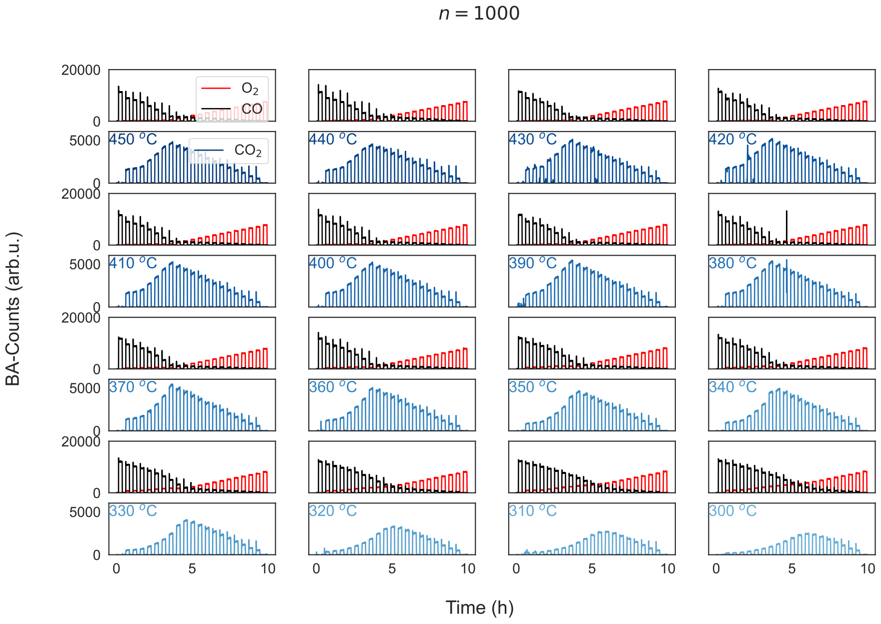

Supplementary Fig. 4 Baseline-adjusted (BA) QMS CO_2, O_2 and CO counts for the $n = 1000$ sample at all investigated temperatures. This figure depicts the raw QMS readings for CO (black), O_2 (red), and CO_2 (blue) for the $n = 1000$ Pd nanoparticle sample, as acquired in our experiments spanning a temperature range of T = 300-450 °C. Each panel corresponds to a different temperature. Intervals of zero signal for CO and O_2 correspond to Ar flushes in between reaction pulses.

Supplementary Figure 4 presents the raw measured BA-Counts for CO, CO_2 and O_2 for the $n = 1000$ Pd nanoparticle sample across a temperature range of T = 300-450 °C. The data is segmented into different panels, each corresponding to a separate temperature. The clear demarcations for intervals of zero signal for CO and O_2 correspond to flushes of Ar between reaction pulses. The signal for CO_2 is measured using the BA-Counts for the $m/z = 44$ channel, which corresponds to the $^{12}C^{16}O_2$ isotope. This channel is the primary channel of interest, as it is the most abundant isotope of CO_2. Interpreting the signal, we see that the CO_2 output is characterized by a series of pulses, each corresponding to a single steady-state reaction event. The CO_2 output is thus a direct representation of the catalytic reaction, and the signal's structure is clearly indicative of the reaction dynamics.

Similarly, Supplementary Figure 5 details the equivalent QMS output for the $n = 10$ Pd nanoparticle sample and Supplementary Figure 6 details the equivalent output on the $n = 1$ Pd nanoparticle sample, representing the scenario of a single nanoparticle.

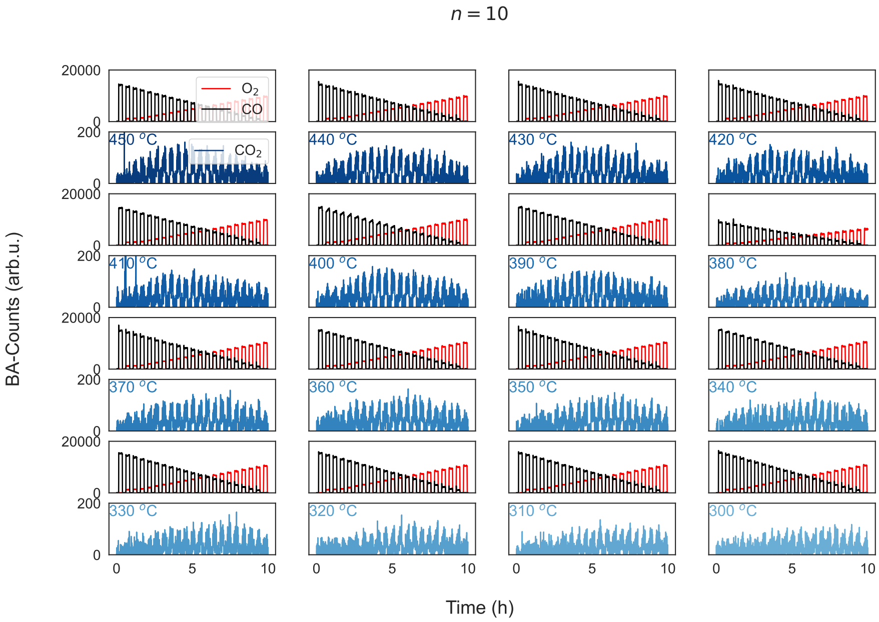

Supplementary Fig. 5 BA-QMS CO_2, O_2 and CO counts for the $n = 10$ sample at all investigated temperatures. This figure depicts the raw QMS readings for CO (black), O_2 (red), and CO_2 (blue) for the $n = 10$ Pd nanoparticle sample, as acquired in our experiments spanning a temperature range of 300-450 °C. Each panel corresponds to a different temperature. Intervals of zero signal for CO and O_2 correspond to Ar flushes in between reaction pulses.

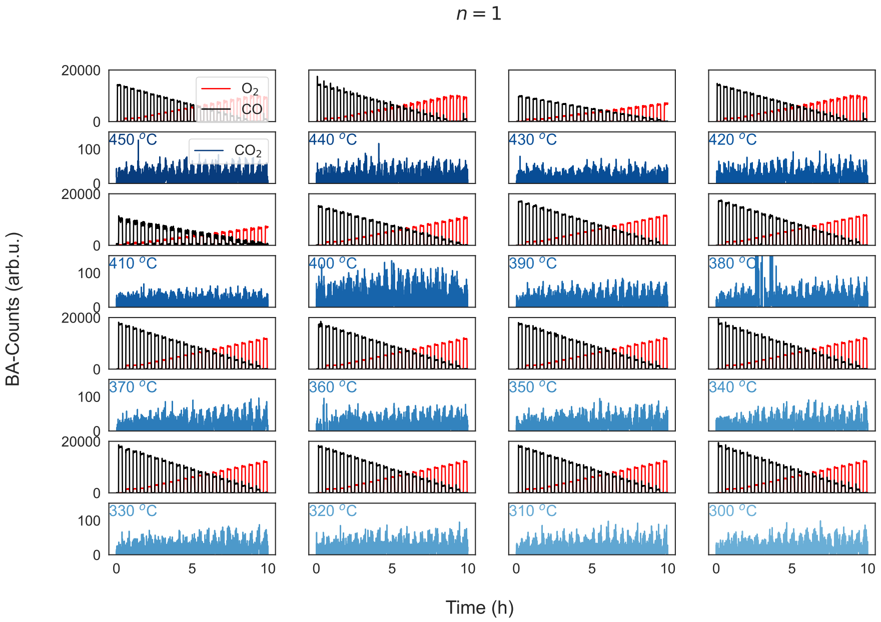

Supplementary Fig. 6 BA-QMS CO_2, O_2 and CO counts for the $n = 1$ sample at all investigated temperatures. This figure depicts the raw QMS readings for CO (black), O_2 (red), and CO_2 (blue) for the $n = 1$ Pd nanoparticle sample, as acquired in our experiments spanning a temperature range of 300-450 °C. Each panel corresponds to a different temperature. Intervals of zero signal for CO and O_2 correspond to Ar flushes in between reaction pulses.

4 Supplementary Note 4. Encoder-Decoders for Denoising

Deep auto-encoders function through a straightforward concept involving a downsampling encoder network and an upsampling decoder network, without any direct links between the two. The encoder's job is to compress the input data into a smaller, essential representation in a space called "latent space," which retains only significant features. The decoder then uses this compact form to reconstruct the original data. While auto-encoders typically aim to replicate the input data precisely, denoising auto-encoders (DAEs) focus on restoring only the relevant parts of the input, filtering out any noise or irrelevant information.

Particularly, DAEs excel when there's a clear distinction between signal and noise—when they're not mixed. This is often the case in systems where meaningful signals are masked by higher-frequency noise. Neural networks, by their nature as piece wise linear approximators, find it easier to capture and represent lower-frequency patterns than higher-frequency ones, making them suitable for this task. In particular, DAEs have been shown to work best when higher level representations of a signal are robust to the corruption of the data, i.e. when noise and signal are uncorrelated [1].

Understanding how neural networks function precisely is complex, but with DAEs, a probabilistic view can offer a comprehensive interpretation. By learning to reverse the corruption process—essentially estimating the original signal from the noisy one—DAEs can be seen as estimating the underlying patterns or distributions in the data.

Table 1 outlines this principle, demonstrating how DAEs are trained to estimate the original distribution of data by alternating between sampling from the corrupted data and the estimated clean data. This method implies that DAEs can be an effective tool for getting to the heart of the data-generation process itself. Essentially, by corrupting random variable X (signal) to $\bar{X}$ through distribution $C(\bar{X}|X)$, what the DAE is really training to accomplish is estimating the reverse conditional distribution $P(X|\bar{X})$ [1]. In [1], they show that by combining this reverse distribution estimator $P(X|\bar{X})$ with the known corruption process $C(\bar{X}|X)$, a simple Markov chain that alternates between sampling from $P(X|\bar{X})$ and $C(\bar{X}|X)$ (equivalent to encoder/decoder) can find the underlying signal distribution $P(X)$. Thus, this suggests that the general performance of DAE networks is effectively to implicitly estimate the underlying data-generating process, and that a simple Markov chain which draws samples from the denoising model and corrupting distribution converges to said estimator.

Regular Denoising Auto-encoders (DAE) have been shown to achieve incredible results in applications ranging from speech recognition [2], to anomaly detection [3] for finding faults in microwave circuits [4] and finding fake twitter followers [5], to LIGO gravitational wave detection [6].

**THE GENERALIZED DENOISING AUTO-ENCODER TRAINING ALGORITHM**
Requires a training set or training distribution $\mathcal{D}$ of examples X, a given corruption process $\mathcal{C}(\tilde{X} \mid X)$ from which one can sample, and with which one trains a conditional distribution $P_\theta(X \mid \tilde{X})$ from which one can sample.
**repeat**<br>- sample training example $X \sim \mathcal{D}$<br>- sample corrupted input $\tilde{X} \sim \mathcal{C}(\tilde{X} \mid X)$<br>- use $(X, \tilde{X})$ as an additional training example towards minimizing the expected value of<br>$-\log P_\theta(X \mid \tilde{X})$, e.g., by a gradient step with respect to θ through the negative log-likelihood loss, until convergence of training.

Supplementary Table 1 The Generalized Denoising Auto-encoder Training Algorithm.

5 Supplementary Note 5. Comparative Studies of the DAE and other denoising techniques

The efficacy of the denoising autoencoder (DAE) was benchmarked against the established denoising techniques of Fourier transform-based filtering [7], Principal Component Analysis (PCA) [8], Total Variation (TV) denoising [9], and Wavelet denoising [10]. The comparative analysis was predicated on the capability to retain the integrity of the original signal post-denoising. In every instance assessed, the autoencoder demonstrated superior noise mitigation while maintaining the fidelity of the original signal, as detailed below.

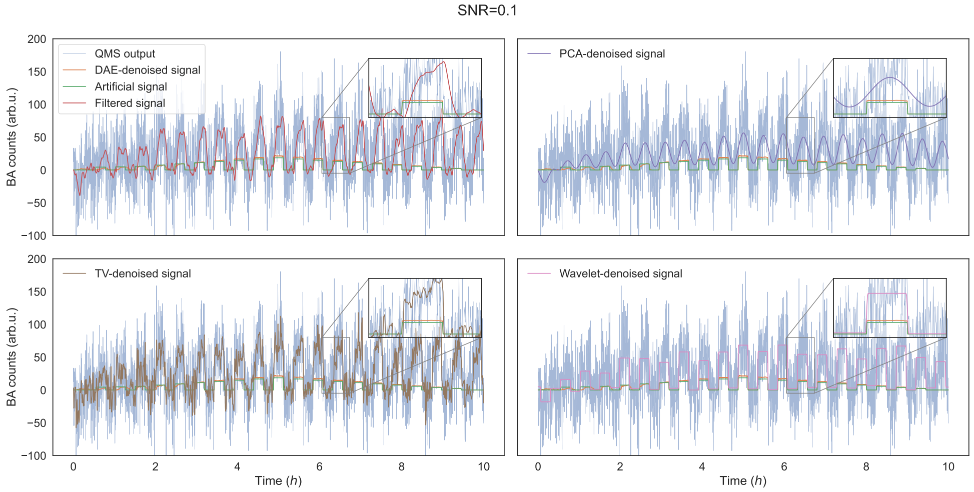

Supplementary Fig. 7 Comparative analysis of signal denoising techniques for signal of magnitude SNR= $\frac{\mu(\text{signal})}{\sigma(\text{noise})} = 0.1$ This figure illustrates the output of the denoising methods we set out to compare, after denoising an artificial QMS output (blue) consisting of true experimental measurement noise acquired from a typical CO oxidation sequence but executed at $T = 40$ °C where essentially no reaction occurs superimposed with an artificial signal pulse (green) with a magnitude defined by signal-to-noise ratio SNR= 0.1. Here, SNR is defined as the ratio of the mean value of the signal (across all CO_2 pulses) to the standard deviation of the noise (across all CO_2 pulses), reflecting the varying degrees of data quality from high-noise (low SNR) to low-noise (high SNR) conditions. The compared denoising methods are the DAE (orange), Fourier transform-based filtering (red), PCA (purple), Total Variation denoising (brown), and Wavelet denoising (pink). The efficacy of each method is evaluated based on its ability to maintain the integrity of the artificial signal pulse, with the DAE showing a clear advantage in noise reduction and retained signal fidelity.

Supplementary Figures 7 and 8 displays the denoising performance of the various methods on a set of artificially created QMS output data consisting of actual QMS measurement noise acquired during a typical CO oxidation sequence used in this work, but executed at $T = 40$ °C where essentially no (measurable) reaction occurs, superimposed to an artificial signal corresponding to a distinct CO_2 pulse. In the following, we discuss the different denoising techniques in more detail.

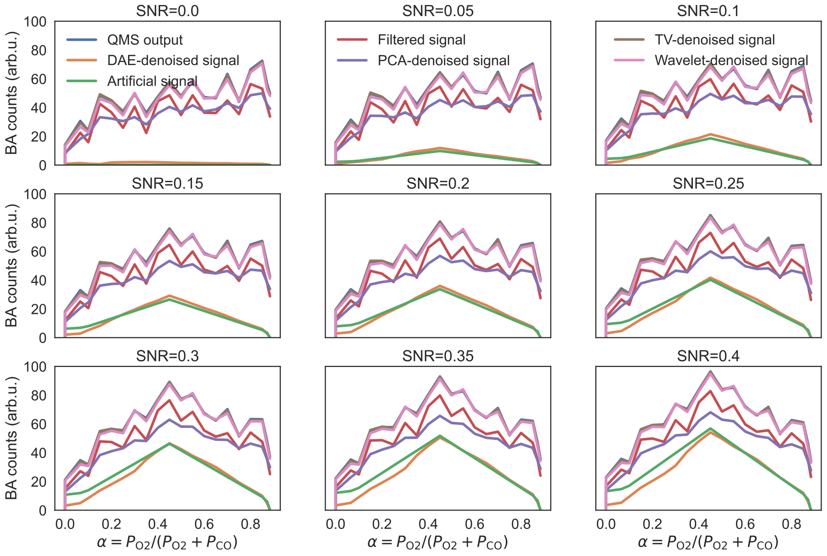

Supplementary Fig. 8 Comparative analysis of signal denoising techniques as function of systematically varying signal magnitude. This figure illustrates the output of the denoising methods we set out to compare, after denoising an artificial QMS output (blue) consisting of true experimental measurement noise acquired from a typical CO oxidation sequence but executed at $T = 40\ ^\circ$C where essentially no reaction occurs, superimposed with an artificial signal pulse (green) with magnitudes defined by signal-to-noise ratios SNR= $[0, 0.05, ..., 0.4]$. The denoising methods encompass the DAE, Fourier transform (Filtered Signal), Principal Component Analysis (PCA), Total Variation denoising (TV), and Wavelet denoising. Notably, the DAE (in orange) consistently reconstructs a signal that closely matches the artificial, noise-free benchmark, demonstrating its superior ability to discern and restore the true signal under diverse and challenging noise environments. For each SNR tested, the only method reconstructing both quantitatively and qualitatively correct (artificial) signal is the DAE, i.e. it is the only method closely following the green line.

Butterworth Filter: A signal processing filter commonly used to filter in the Fourier domain when the frequency response characteristics of the underlying signal is known. For our QMS data, a low-pass Butterworth filter was applied to allow low-frequency components (representing the true signal, i.e., here the artificial CO_2 pulses) to pass through while attenuating higher-frequency noise. The filter's cutoff frequency was determined based on the frequency spectrum of the QMS data to ensure minimal distortion of the original signal [7].

The signal, a series of (here artificial) CO_2 pulses, inherently contains a broad spectrum of frequency components. The primary components are the low-frequency elements that represent the step changes, and these are accompanied by higher-frequency components introduced by the noise inherent to the system. Given that the noise is distributed normally, it is reasonable to assume that it is white noise evenly distributed across all frequencies. This type of noise contains high-frequency components that are

not present in the original signal, introducing a form of distortion that we aim to mitigate through filtering.

A low-pass filter is appropriate in this scenario because it allows the low-frequency components of the signal to pass while attenuating the high-frequency noise. The critical parameters to consider are the cutoff frequency and the order of the filter. The cutoff frequency should be chosen to be slightly higher than the highest frequency component of the signal that we wish to retain, ensuring that the essential characteristics of the signal are preserved while filtering out the high-frequency noise components [11].

The filter order influences the roll-off characteristics at the cutoff frequency. A lower order typically provides a gentler transition at the cutoff, potentially preserving more of the signal characteristics but possibly being less effective in removing noise. A higher order will offer a steeper roll-off, more effectively removing noise but potentially distorting the underlying signal more [11].

Therefore, an initial approach typically involves selecting a lower filter order, such as a second-order filter, to maintain a substantial portion of the signal characteristics, and iteratively testing higher-order filters. The sample rate should be determined based on the data acquisition system, and the cutoff frequency should start at a value that is determined based on the frequency content of the signal with adjustments also made iteratively based on the results [11]. Doing so, we found a butterworth filter with order $O = 2$ and cutoff frequency $f = 500$ Hz.

The output of the filter, when applied to benchmark QMS output, is visible as the red line in Supplementary Figures 7 and 8. Here, it is clear that whilst the filter successfully suppresses the impact of higher-frequency components on the underlying signal, it is incapable of reconstructing the correct structure or the quantitatively correct CO_2 output of the artificial superimposed signal. Its performance is further elaborated upon and discussed in the section below and in Supplementary Figure 9.

Another approach for denoising is by means of Principal Component Analysis (PCA), where the original data variables are transformed into a new set of orthogonal variables known as principal components. These components encapsulate the maximum data variance. For the QMS CO_2 output, PCA was applied by first determining the number of principal components that captured a significant portion of the data variance. Subsequent components, which were deemed to represent noise, were discarded, and the signal was then reconstructed using only the retained principal components. In practice, we first transformed the data into a Hankel matrix, a process mathematically represented as [8]:

$$H = (o[0 : L], o[L - 1 : N]) \tag{1}$$

where H is the Hankel matrix, o is the original signal, L is the number of rows in the Hankel matrix, and N is the length of the signal. In our specific case, we chose $L = 200$, which means that each column in the Hankel matrix is a delayed version of a 200-point segment of the original measurement. Subsequently, we applied PCA to the

Hankel matrix to obtain the principal components of the data. The transformation is given by:

$$H_{\mathrm{PCA}} = \mathrm{PCA}(H) \tag{2}$$

where H_{PCA} is the matrix of principal components.

To denoise the data, we retained only the first principal component, effectively reducing the noise by discarding the other components which are assumed to contain noise and less important information about the true signal dynamics. This process can be mathematically represented as:

$$H_{\mathrm{d}} = H_{\mathrm{PCA}}[:, :1] \tag{3}$$

where H_{d} is the denoised version of the PCA-transformed Hankel matrix, retaining only the first column (principal component). Following this line, we reconstructed the Hankel matrix using the inverse PCA transformation:

$$H_{\mathrm{r}} = \mathrm{PCA}^{-1}(H_{\mathrm{d}}) \tag{4}$$

where H_{r} is the reconstructed Hankel matrix from the denoised principal components. Finally, we reconstructed the denoised signal from the reconstructed Hankel matrix. Given the overlapping entries of the Hankel matrix, the signal was obtained by averaging over the anti-diagonals, a process described as:

$$s[k] = \frac{1}{\min(k+1, L, N-k)} \sum_{i=\max(0,k-N+L)}^{\min(k,L-1)} H_{\mathrm{r}}[i, k-i] \tag{5}$$

where s is the reconstructed denoised signal.

This method leverages the PCA technique to effectively reduce noise while retaining the essential characteristics of the signal. The output of the filter, when applied to benchmark QMS output, is visible as the purple line in Supplementary Figures 7 and 8. Here, it is clear that reducing the dimensionality of the feature space and reconstructing it from a low-dimensional representation is inducive to both suppressing higher-order frequency contributions to the noise and retaining the overall periodic nature of the underlying signal. However, the structure of the signal is only reconstructed correctly on average and with overall low fidelity (as is expected from a direct dimensionality reduction), leading to a reconstructed underlying signal which is roughly correct on average everywhere and exactly correct essentially nowhere. Note, in particular, how the denoised QMS output starts to increase in anticipation of CO_2 pulses which have not occurred yet (in time). Its performance is further elaborated upon and discussed in the section below and in Supplementary Figure 9.

Total Variation Denoising: This technique focuses on reducing the total variation of the denoised signal. For our QMS data, the total variation denoising algorithm was applied

iteratively, with a regularization parameter optimized to strike a balance between noise reduction and preservation of sharp features in the data. The QMS output was subjected to a two-stage denoising process.

In the initial stage, we applied total variation denoising, a method formulated to minimize the following objective function [9]:

$$J(x) = \frac{1}{2}\sum_{i=1}^{N}(s_i - o_i)^2 + \lambda \sum_{i=1}^{N-1} |s_{i+1} - s_i| \tag{6}$$

where s is the denoised signal, o is the observed noisy signal, N is the number of data points, and λ is a regularization parameter that controls the trade-off between data fidelity and noise suppression. In our specific implementation, we use $\lambda = 1$. The output of the filter, when applied to benchmark QMS output, is visible as the brown line in Supplementary Figures 7 and 8.

The iterative nature of the algorithm in princple allows for a more specialized approach to signal refinement. By iteratively adjusting the regularization parameter and reassessing the denoised signal, the algorithm finely balances the dual objectives of reducing noise and preserving key signal features. This iterative process is particularly crucial for QMS output, where the signal's sharp transitions—indicative of real-time chemical reactions—must be maintained for accurate analysis. However, despite its strengths in preserving sharp signal features, total variation denoising tends to only smooth out the noise, resulting in an inaccurate reproduction of quantitatively correct signal characteristics that are crucial in interpreting QMS data. The method's performance is further elaborated upon and discussed in the section below and in Supplementary Figure 9.

Wavelet-based denoising: In our analysis, we adopted an iterative approach to implement an edge-enhanced and smoothed denoising technique, which was built upon the wavelet thresholding method [10]. This approach was chosen to preserve the step-function characteristics of the original signal, again retaining sharp transitions while reducing noise.

Initially, the noisy signal was denoised using the Daubechies wavelet with one level of decomposition and soft thresholding. The optimal threshold value was determined through a process of minimization of the Mean Absolute Error (MAE) between the denoised and the original signals, defined as:

$$\text{MAE} = \frac{1}{N}\sum_{i=1}^{N} |o_i - \hat{s}_i| \tag{7}$$

where N is the number of samples, o_i is the original signal, and $\hat{s}_i$ is the denoised signal.

Following this line, we enforced step-function characteristics on the denoised signal by segmenting it into different regions, each corresponding to a step in the step-function,

and assigning a constant value to all points within each region. The constant value was derived from the reconstructed signal, using the mean of the values within the respective region.

To further enhance the denoised signal, we applied an edge-enhancement technique to create more distinct transitions between steps. Lastly, to smooth out minor fluctuations and further approximate a step function, a Gaussian smoothing filter with a standard deviation (σ) of 1 was applied to the edge-enhanced signal. The output of the filter, when applied to benchmark QMS output, is visible as the pink line in Supplementary Figures 7 and 8. Note in particular the consistency of this method to reconstruct the underlying structure of the signal, as we enforce through our prior in the edge-enhanced smoothing. However, the quantitative values of the reconstructed signal in each pulse are still incorrect and approache the mean of the noisy QMS output. Its performance is further elaborated upon and discussed in the section below and in Supplementary Figure 9.

Discussion and comparison of different denoising methods

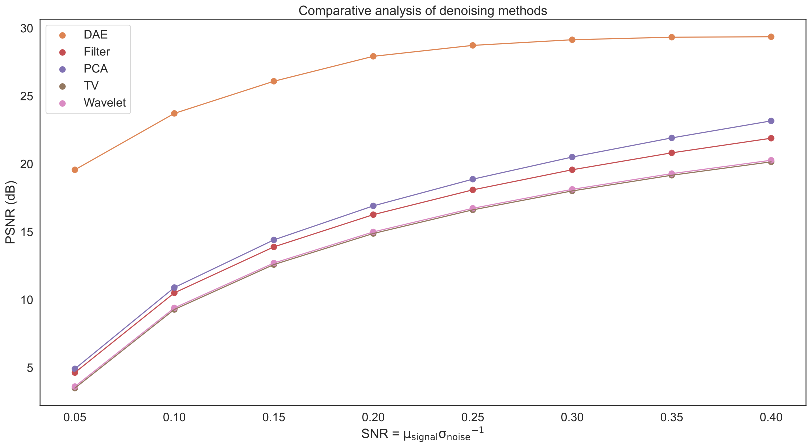

Supplementary Fig. 9 Comparative Performance of Denoising Techniques using Peak Signal-to-Noise Ratio (PSNR) as descriptor. This figure illustrates the PSNR in decibels (dB) for the investigated denoising techniques DAE, Low-Pass Butterworth Filtering, Principal Component Analysis (PCA), Total Variation (TV) Denoising, and Wavelet Denoising, applied to artificial QMS data representing artificial CO_2 pulses superimposed to experimentally measured noise. Each technique is evaluated against the unprocessed baseline to assess improvements in signal clarity and noise reduction. The PSNR values provide a quantitative measure of the extent to which each denoising method preserves the true signal while mitigating the influence of noise inherent in the system. As the key result, we see that the DAE significantly outperforms traditional denoising methods at all SNRs tested, particularly for lower SNR values, in terms of PSNR.

We utilized the Peak Signal-to-Noise Ratio (PSNR) as a metric to evaluate the effectiveness of our different denoising techniques and compare them to the DAE used in the main text. PSNR is traditionally utilized in the field of image and video processing to quantify the quality of reconstructed or compressed images [12] because it offers a clear and quantifiable way to assess the quality of a denoised signal. In essence, PSNR measures how much the denoised signal deviates from the original, noise-free signal. A high PSNR indicates that the denoised signal is very close to the original signal, while a low PSNR suggests that the denoising process may have introduced distortions or failed to adequately reduce noise. In the field of image and video processing, PSNR is commonly used because it is directly related to the mean squared error between the original and the reconstructed images or signals. It's a standard measure that provides a straightforward comparison between the original and processed signals across different methods or algorithms [13].

The metric is defined mathematically as:

$$\text{PSNR} = 20 \cdot \log_{10} \left(\frac{\text{MAX}_I}{\text{RMSE}} \right) \tag{8}$$

where MAX_I is the maximum possible value of the signal, and RMSE is the Root Mean Square Error calculated between the original and the denoised signals. The RMSE is defined as:

$$\text{RMSE} = \sqrt{\frac{1}{N} \sum_{i=1}^{N} (o_i - s_i)^2} \tag{9}$$

where N is the total number of samples, and o_i and s_i represent the original and the denoised signal values at the i-th sample, respectively.

MAX_I is defined as the maximum value in the original signal, establishing the peak potential value in the dataset and serving as a normalization factor in the PSNR calculation. A higher PSNR value indicates that the denoised signal is of higher quality, with a lesser amount of noise or error introduced during the denoising process. Conversely, a lower PSNR value would imply a greater discrepancy between the original and denoised signals, highlighting a higher level of noise or error.

While a higher PSNR generally indicates a closer approximation to the original signal, it does not necessarily correlate with perceptual quality, particularly in fields such as image and video processing [14]. However, given that our study focuses on the output of a QMS, where the objective is to maintain the quantitative integrity of the signals rather than perceptual quality, the PSNR serves as an apt metric for our evaluations. In the broader perspective, PSNR serves as a comparative metric, aiding in the discernment of the most effective denoising technique when several methods are juxtaposed.

In this present work, our goal is to apply a denoising technique that not only preserves the essential characteristics of the QMS signal but also ensures a high PSNR value,

indicating a lower level of noise and a higher fidelity to the original signal. Comparing the PSNR values for all denoising methods tested, the DAE demonstrates a distinct advantage in preserving the integrity of the original signal while effectively reducing noise compared to all tested traditional denoising methods (Supplementary Figure 9). This comparative study thus reinforces our decision to adopt the DAE as the primary denoising technique in this work, and underscores the superiority of the DAE over the traditional denoising techniques of Fourier transform-based filtering, Principal Component Analysis (PCA), Total Variation (TV) denoising, and Wavelet denoising when aiming at denoising pulse-like signals. Supplementary Figures 7, 8, and 9 collectively demonstrate the DAE's robust performance across varying levels of signal-to-noise ratios.

With this result at hand, it is now interesting to briefly address the underlying reasons. In brief, the DAE's fundamental advantage lies in its ability to learn and model the underlying structure of the noise-free signal through a training process and directly incorporate the real measurement noise. Unlike the other methods, which rely on predetermined transformations or decompositions, the DAE dynamically adjusts its parameters to minimize reconstruction error, allowing for a more flexible and adaptive approach to noise reduction.

Traditional filtering and decomposition methods, while effective in certain scenarios, often operate under rigid assumptions about the signal and noise characteristics. For instance, Fourier and Wavelet denoising assume that noise can be separated in the frequency domain, which may not always align with the complex nature of real-world data. PCA attempts to reduce noise by considering variance, which can inadvertently remove important signal components if the noise also varies significantly. TV denoising presumes that the signal has a piecewise constant nature, which may not be suitable for signals with more nuanced variations.

In contrast, the DAE does not require explicit assumptions about the signal or noise. It learns an optimal set of filters from the data itself, enabling it to capture and reconstruct the true signal more accurately, as evidenced by the consistently higher Peak Signal-to-Noise Ratio (PSNR) values shown in Supplementary Figure 9. This adaptability is crucial in processing the complex QMS data obtained from the CO oxidation sequences in this work, where the signal exhibits non-trivial patterns that are not readily isolated using traditional methods. Moreover, the DAE's proficiency in handling lower SNR (higher noise) conditions without compromising signal integrity suggests its potential for applications beyond the scope of this study. It could be particularly beneficial in fields where the signal is weak and deeply embedded in noise, wherein the field of single particle catalysis is a clear case, necessitating a sensitive and nuanced approach to denoising. In conclusion, the DAE emerges as the most competent and versatile tool for denoising the QMS data in this study due to its unmatched ability to maintain signal fidelity while effectively suppressing noise.

6 Supplementary Note 6. Complete n=1 and n=0 data set of Fig. 5 in main text

Supplementary Figure 10 contains additional data obtained from measurements either at 450 °C for standard analysis of CO_2 counts (a) or at additional intermediate temperatures for the DAE analysis (b). These data were not included in Figure 5 in the main text for clarity.

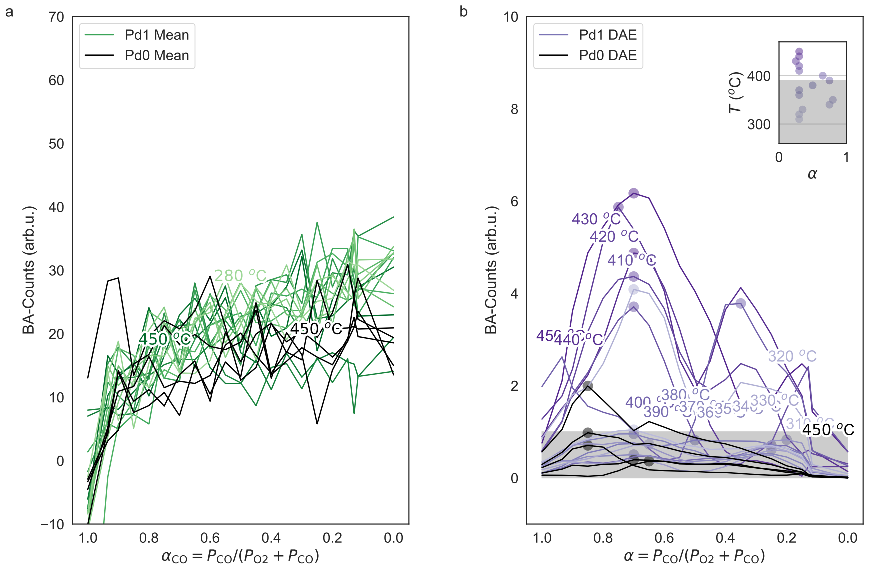

Supplementary Fig. 10 Mass spectrometry from a single Pd nanoparticle and an empty nanochannel control. (a) Mean BA-CO_2 counts extracted for each α_{CO} pulse based on the standard analysis across the entire α_{CO} range, $\alpha_{CO} \in (0, 1)$, and at 450 °C, for both $n = 0$ (black lines) and $n = 1$ (green lines). For $n = 1$ we executed 15 consecutive α_{CO} sweeps and for $n = 0$ we executed 5 consecutive α_{CO} sweeps. (b) Same as (a) but for DAE-denoised data and for a range of temperatures indicated in the figure, where distinct reaction rate maxima are resolved for $n = 1$ (purple curves).

7 Supplementary Note 7. Synthetic Data Generation & Deep Learning Training

Synthetic data generation is a two-fold process that involves creating a realistic simulation of the expected signal and the various noise components that are typically encountered in QMS data.

The noise model comprises two distinct generative components. The first component simulates intrinsic noise sources inherent to QMS devices, including thermal noise, environmental fluctuations, flicker noise, shot noise, and ion feedback noise. We employ a Gaussian noise model with a wide standard deviation (σ_{noise}) range, defined in the code through a specified signal-to-noise ratio $SNR = \frac{\mu_{signal}}{\sigma_{noise}}$ to encapsulate the broad spectrum of white noise effects characteristic of these sources. This white noise serves as the base upon which other noise factors are superimposed. This noise component is shown in Supplementary Figure 11a.

The second component of the noise generation accounts for contamination noise, which arises from reagent reactions occurring outside the catalytically active surface area of the nanoparticles. This noise is modeled as a variable term added to each gas pulse. It is proportional to the concentration of input reagents, reflecting the stochastic nature of these reactions and their impact on the measured QMS output. This noise component is shown in Supplementary Figure 11b, and the corresponding combined simulated noise is shown in panel c.

The synthetic signal is constructed as a step function characterized equivalently by the signal-to-noise ratio SNR= $\frac{\mu_{\text{signal}}}{\sigma_{\text{noise}}}$. The SNR settings are calibrated to mirror the experimental data obtained from $n = 1$ and $n = 10$ reactor readouts, providing a realistic baseline for the signal amidst the generated noise. Within this range, the signal for each individual pulse is randomly generated, whilst ensuring that the mean value of all pulses together correspond to the defined SNR. The signal's step function follows a consistent on-off pulsing pattern, with the amplitude of each pulse randomly determined, as mentioned, to ensure a diverse set of signal magnitudes without introducing any inductive bias related to the sequence or relationship of pulses within the step function. These signals are represented in Supplementary Figure 12.

Supplementary Figure 13 shows a representative subset of the data used to train the DAE, consisting of artificial signal as shown in Supplementary Figure 12, superimposed on either artificial noise or experimentally measured noise, as shown in Supplementary Figure 11.

Training procedure

The training procedure is bifurcated into two principal stages, each designed to incrementally develop the network's ability to denoise the QMS signals effectively.

Initially, a curriculum learning approach is implemented, where the network is exposed to artificially generated signals superimposed on Gaussian-distributed noise with a pre-defined SNR. This SNR is selected from a uniform distribution with initial bounds

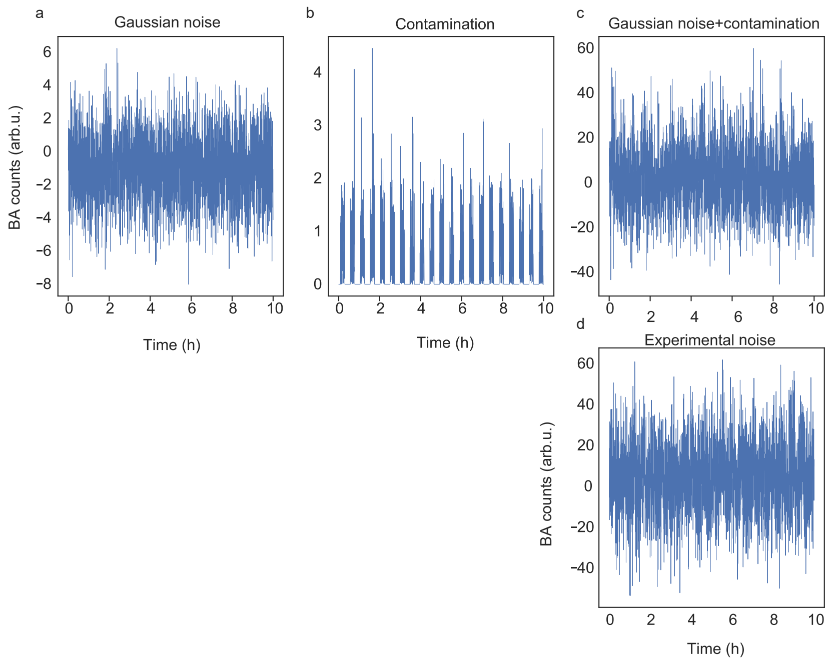

Supplementary Fig. 11 Illustration of noise components in synthetic data generation. a) displays the Gaussian-distributed white noise simulating intrinsic QMS noise sources, such as thermal and environmental fluctuations. b) depicts the contamination noise model, a superimposed noise based on contamination from gas lines, proportional to the input reagent concentrations. c) combines both noise components, representing the total simulated noise profile used for training the denoising algorithm. d) showcases an experimental noise sample from actual QMS measurements, providing a "real-world" reference for comparison with the simulated noise profiles. This noise is measured from a typical CO oxidation sequence that was executed at $T = 40$ °C, where (almost) no reaction occurs.

set at SNR$\in (0.95, 1)$, reflecting the high catalytic activity observed in the experimental data of the $n = 10$ Pd sample at $T = 450\ °C$. The curriculum learning strategy systematically lowers the SNR threshold by increments of 0.05 upon each convergence of the network, culminating at a minimum SNR of 0. This gradual reduction mitigates the risk of the network encountering vanishing gradients or mode collapse when faced with extremely low-SNR scenarios. The diverse noise and signal configurations presented during this phase bolster the model's resilience, ensuring it learns robust denoising functions that are broadly applicable across relevant signal and noise ranges.

In the subsequent fine-tuning phase, the network is trained on a range of artificial signal configurations with SNR$\in (0, 1)$, covering the entire spectrum of SNRs addressed in this study. These signals are overlaid on noise profiles derived from experimental measurements, specifically the QMS output from a CO oxidation sequence using an $n = 0$ Pd sample chip at $T = 40$ °C. This stage is critical for aligning the network's

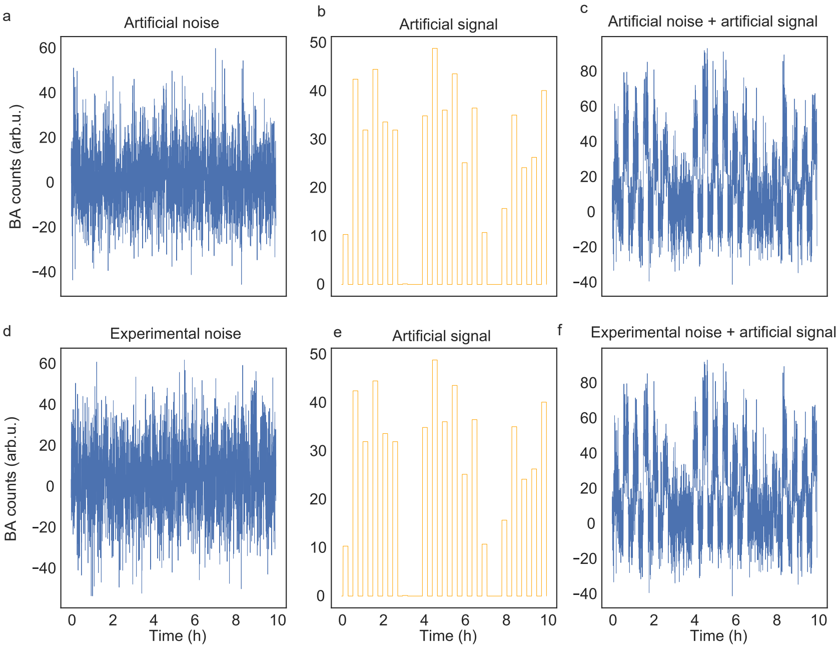

Supplementary Fig. 12 Synthetic and experimental signal and noise integration. a) displays the Gaussian-distributed white noise simulating intrinsic QMS noise sources, such as thermal and environmental fluctuations. b) presents an artificially generated step function signal, each step with a randomly assigned amplitude, designed to simulate the signal expected from the catalytic reaction. c) overlays this artificial signal onto the simulated noise, illustrating the combined signal-noise interaction as it would appear in the synthetic dataset. d-f) demonstrates the integration of experimentally measured noise with the artifical signal in an equivalent manner,representing the realistic scenario that the denoising algorithm is ultimately intended to address. Here, the noise measured from a typical CO oxidation sequence that was executed at $T = 40\ °\mathrm{C}$.

learned functions with the actual noise distributions it will encounter during practical application, ensuring that the denoising performance is optimized for real-world conditions.

Loss Curve

The loss function in a machine learning model quantifies the difference between the predicted and true values. Firstly, to follow the recipe of a generalized denoising autoencoder outlined in [1] and Table 1, our main loss function should be negative log-likelihood (NLL).

For regression tasks, the equivalent of the NLL loss often arises when we make certain probabilistic assumptions about the noise or the errors in our predictions. The choice of the NLL for regression fundamentally depends on the assumed distribution of the

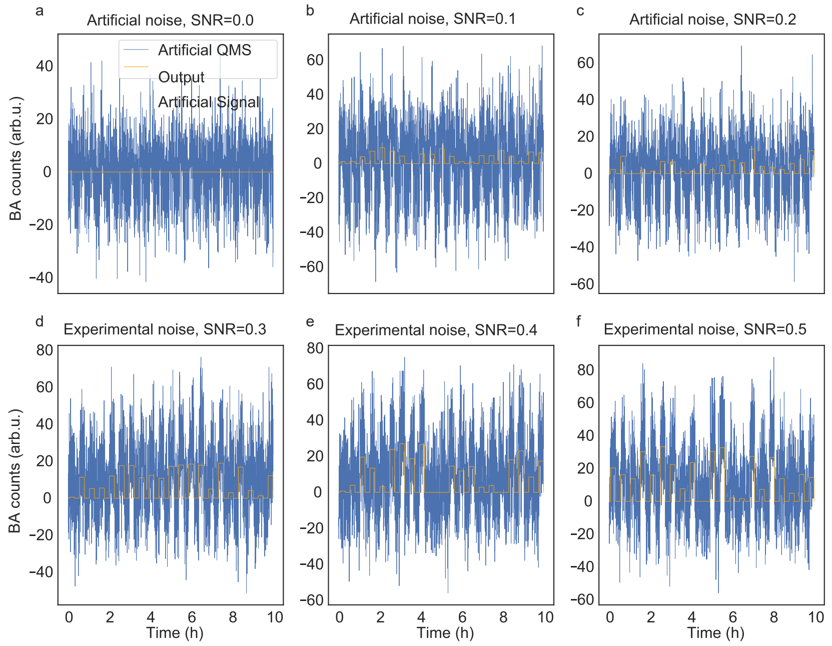

Supplementary Fig. 13 Representative data during training. This figure illustrates a small representative subset of the training data, where panels (á-c) show artificial QMS output defined by SNR $\in$ $[0, 0.1, 0.2]$. It was constructed with artificially generated signal superimposed on artificially generated noise. Panels (D-F), similarly, show artificial QMS output defined by SNR $\in$ $[0.3, 0.4, 0.5]$. It was constructed with artificially generated signal superimposed on experimentally measured noise defined as a typical CO oxidation sequence but executed at $T = 40$ °C, where (almost) no reaction occurs. Note that the SNR, noise and signal characteristics are randomly sampled and varied during training.

residuals (the differences between predicted values and true values). The most common assumption for regression tasks is that the residuals follow a Gaussian (or normal) distribution. This assumption leads to the Mean Squared Error (MSE) loss:

$$\text{MSE}(\theta) = \frac{1}{N} \sum_{i=1}^{N} (y_i - f_{D\theta}(x_i; \theta))^2 \tag{10}$$

where y_i is the true value for the i-th data point. $f_{D\theta}(x_i; \theta)$ is the decoder's output for the i-th data point, with parameters θ. N is the number of data points.

Under the Gaussian noise assumption, the MSE arises naturally as the NLL. To see this, consider the likelihood for a single data point given by the Gaussian probability density function:

$$\mathcal{L}(y_i|x_i;\theta) = \frac{1}{\sqrt{2\pi\sigma^2}} \exp\left(-\frac{(y_i - f_{D\theta}(x_i;\theta))^2}{2\sigma^2}\right) \tag{11}$$

Taking the negative logarithm and ignoring terms that don't depend on θ (since they won't affect the optimization), we arrive at a cost proportional to the MSE [15]. This is also typically referred to as an L2, or L^2 norm.

Further, to form the consistency loss in the latent space of the autoencoder, we implement an MAE (equivalently L1 norm) directly between a (squeezed) vector representation of the latent space and the true values of each CO_2 pulse of the underlying (artificial) signal.

$$\text{MAE}(\theta) = \frac{1}{n}\sum_{i=1}^{n}(y_i - f_{B\theta}(x_i;\theta)), \tag{12}$$

where y_i is the true value for the i-th data point, $f_{B\theta}(x_i;\theta)$ is the latent space of the auto-encoder for the i-th data point, with parameters θ and n is the number of CO_2 pulses of the signal.

Following this, we compute the L1 norm between these predictions and the mean predicted output across each CO_2 pulse, enforcing a consistency between the decoder's final predicted signal and its equivalent representation in the latent space of the full auto-encoder.

Mathematically, this can be represented as:

$$C_{\text{loss}}(\theta) = \text{MAE}\left(f_{B\theta}(x_i;\theta), \mu\left(f_{D\theta}(x_i;\theta)_{\text{pulse}[i]}\right)\right) \tag{13}$$

Here, $\mu\left(f_{D\theta}(x_i;\theta)_{\text{pulse}[i]}\right)$ represents the mean output of the decoder across pulse i.

A graphical representation of the loss curve throughout this training regimen is presented in Supplementary Figure 14, illustrating the network's progression and convergence across different SNR levels. The loss, represented in blue, is a measure of how well the model's predictions match the actual signals. It's calculated using a sum of losses Eq. 10, Eq. 12 and Eq. 13, which aggregates the errors across all predictions made for a given set of training data.

As the epochs (training iterations) increase, we observe a steady decline in the total loss, indicating the model's learning and adaptation. This is a typical behavior of a well-tuned model, where the error decreases as it learns from the data. However, we incorporate a curriculum learning approach where the complexity of the training data is progressively increased. This is visually represented by the red-orange lines, which show the minimum SNR of the synthetic data that the model is exposed to at each epoch.

Curriculum learning is designed to mimic a natural learning progression, starting with simpler, less noisy data and gradually introducing more complexity and noise. This

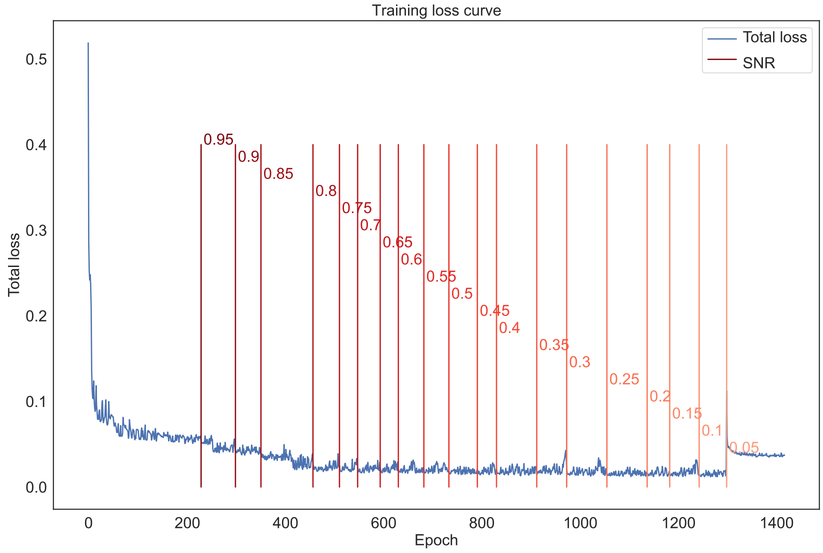

Supplementary Fig. 14 Loss curve during training. The graph presents the trajectory of the total loss during the training of the DAE (blue). This loss curve indicates the optimization process, with a clear downward trend as the model improves with each epoch. Overlaying this trend is the progression of the signal-to-noise ratio (SNR) used in the curriculum learning strategy for generating synthetic data (red). The SNR values start high, signifying easier learning tasks, and gradually decrease, presenting more challenging scenarios for the model to learn from. The inverse correlation between the decreasing SNR and the total loss suggests that the model is effectively adapting to more difficult tasks over time.

technique has been shown to improve the convergence speed and the final performance of deep learning models, especially in scenarios where the data is inherently noisy or complex [16], as is the case in our study. In the early training phase, the model is presented with high SNR data, which is easier to learn from. As the model's capacity to discern patterns and make predictions improves, the SNR is lowered, thus increasing the difficulty of the training samples. This is done to ensure that the model does not overfit on the 'easy' data and can generalize well to more realistic, noisier data. The interleaved spikes in the red SNR curve correspond to the planned reduction in SNR at specific epochs, challenging the model to maintain performance despite the increased noise level.

The results shown in Supplementary Figure 14 demonstrate the effectiveness of our training regimen. Despite the SNR's gradual decrease, the model's total loss continues to decrease, albeit with some variability, indicative of the model learning to cope with the increasingly difficult data. This variability is expected and is a sign that the model is not merely memorizing the training data but is learning robust features that generalize across varying levels of noise.

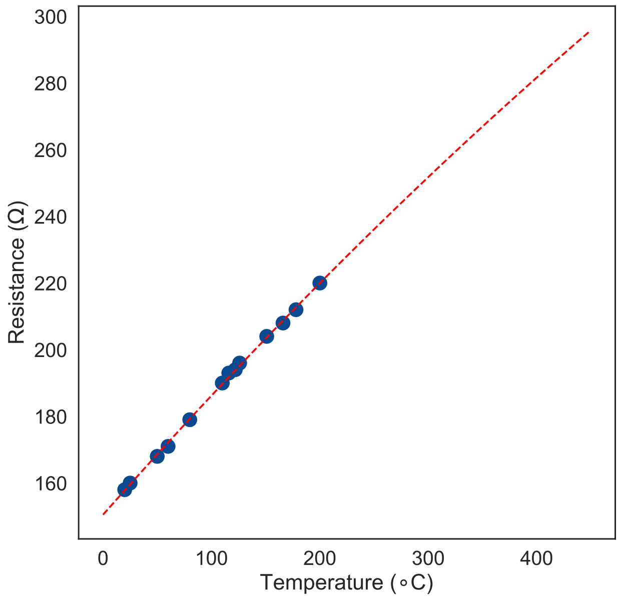

Supplementary Fig. 15 Calibration curve for the on-chip heater. Blue circles represent the measured resistance at specific temperatures, and the red line corresponds to the fitted function based on the Callendar-Van Dusen polynomial, used for extrapolating the response at elevated temperatures.

8 Supplementary Note 8. Calibration of Resistive Nanofluidic Chip Heaters

Calibration of the nanofluidic chips' resistive Pt heaters is essential for precise temperature control during catalytic reaction experiments. To calibrate the heater, the nanofluidic chip is placed in an oil bath and a thermocouple was connected to the chip to monitor its temperature. The temperature of the oil bath was then increased incrementally from 20 °C to 200 °C. At each step, a 10-minute equilibration period ensured temperature stabilization within the nanochannels. Thermocouple readings were recorded to generate a temperature profile as shown in Supplementary Figure 15.

The heater's operation is managed by supplying current to the Pt film via an external power supply. The temperature is read using the four-point probe method [17], where the resistance of the Pt film is monitored. This resistance varies in a predictable manner with temperature [18].

The collected data was fitted to a Callendar-Van Dusen polynomial [19] of the form:

$$R(T) = R_0(1 + \alpha T + \beta T^2) \tag{14}$$

Here, $R(T)$ is the resistance at temperature 0°C, and $R_0, \alpha,$ and β are the coefficients. The fitted data and the curve based on this equation are shown in Supplementary Figure 15, yielding $R_0 = 150.5\Omega, \alpha = 2.4 \cdot 10^{-3}\text{K}^{-1}\text{and}\beta = -6.5 \cdot 10^{-7}\ \text{K}^{-2}$.

Both the heater and the four-point probe thermometer are linked to a Lakeshore temperature control unit that functions as a proportional–integral–derivative (PID) regulator. This unit can set and maintain a specific temperature with an error of less than 0.1 °C. The current design allows for a working temperature range of $20-450$ °C. The time required to change the chip's temperature is typically less than 1 s. Due to the chip's small dimensions, a substantial temperature shift from room temperature to 450 °C takes less than 10s. Cooling is achieved passively, and a temperature drop from 450 to 20 °C takes $\approx$ 20 s.

9 Supplementary Note 9. Ethylene hydrogenation on 1000 Pd nanoparticles

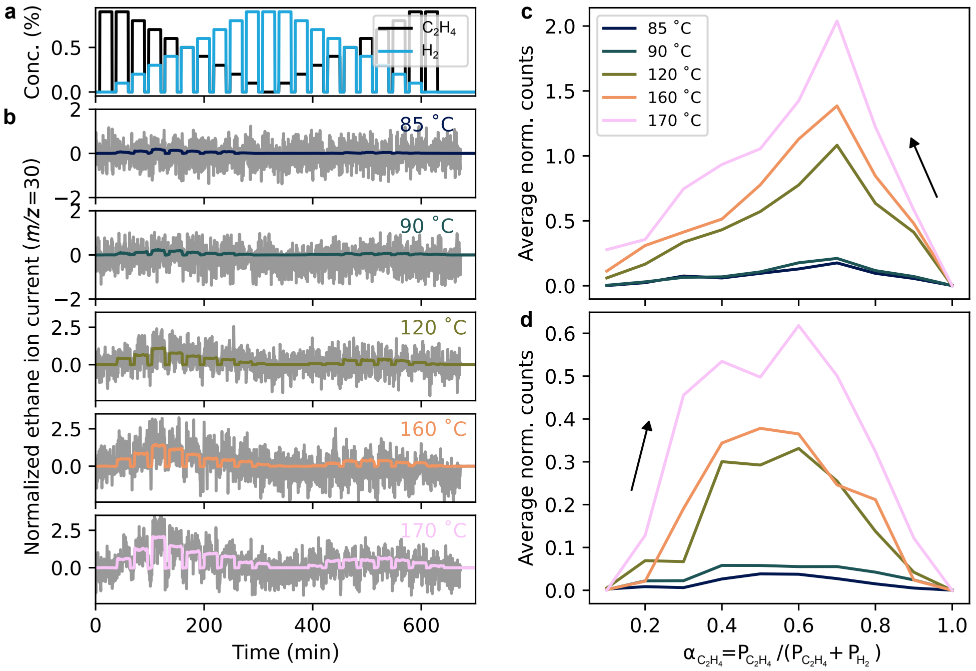

Supplementary Fig. 16 Ethylene hydrogenation experiments at different temperatures on 1000 Pd nanoparticles. (a) Gas concentration of C_2H_4 and H_2 applied in pulsed sequence mode. (b) Baseline-adjusted (BA – see Methods for explanation) normalized ethane counts measured by QMS at $m/z = 30$ (grey lines), together with the signal denoised by the DAE (colored lines) for 85°C, 90 °C, 120 °C, 160 °C, and 170 °C. The colors refer to the corresponding temperatures shown in panel (c). (c, d) BA normalized counts as a function of the mixing parameter $\alpha_{C_2H_4} = P_{C_2H_4}/(P_{C_2H_4} + P_{H_2})$, calculated for every step of the pulse sequence shown in (a). The arrows indicate the direction of the sequence: in (c) $\alpha_{C_2H_4}$ goes from 1 to 0, in (d) from 0 to 1. The colors in (d) match the temperatures reported in (c).

10 Supplementary Note 10. Control Experiment: Detection of Pulsed CO_2 in the Absence of Pd Nanoparticles

To validate that the CO_2 signal detected during catalysis arises solely from surface reactions on the Pd nanoparticles and not from extraneous sources (e.g., gas system artifacts, adsorption/desorption from chip walls or background contributions), we performed a control experiment using a nanofluidic chip intentionally fabricated without

any catalytically active material. The chip design matched the geometry and internal volume of the chip used for catalytic experiments with 1000 Pd nanoparticles.

Pulses of CO_2 diluted in Ar were introduced at a total pressure of 2 bar and a flow rate of 20 ml/min, while maintaining the system at 350 °C. The pulse sequence was designed to span a range of CO_2 concentrations, ultimately reaching levels where the raw QMS signal faded into the noise. This enabled us to test the effectiveness of the DAE in retrieving low-intensity CO_2 signals under non-catalytic conditions.

Figure 17A shows the controlled pulsing of CO_2 into an Ar background. In Figure 17B, the background-subtracted raw signal (grey) and the DAE-denoised output (purple) are presented. Even at low signal levels, the DAE reconstruction clearly resolves the temporal structure of the CO_2 pulses, demonstrating the capacity of the algorithm to extract meaningful signal from noisy measurements in the absence of catalytic activity.

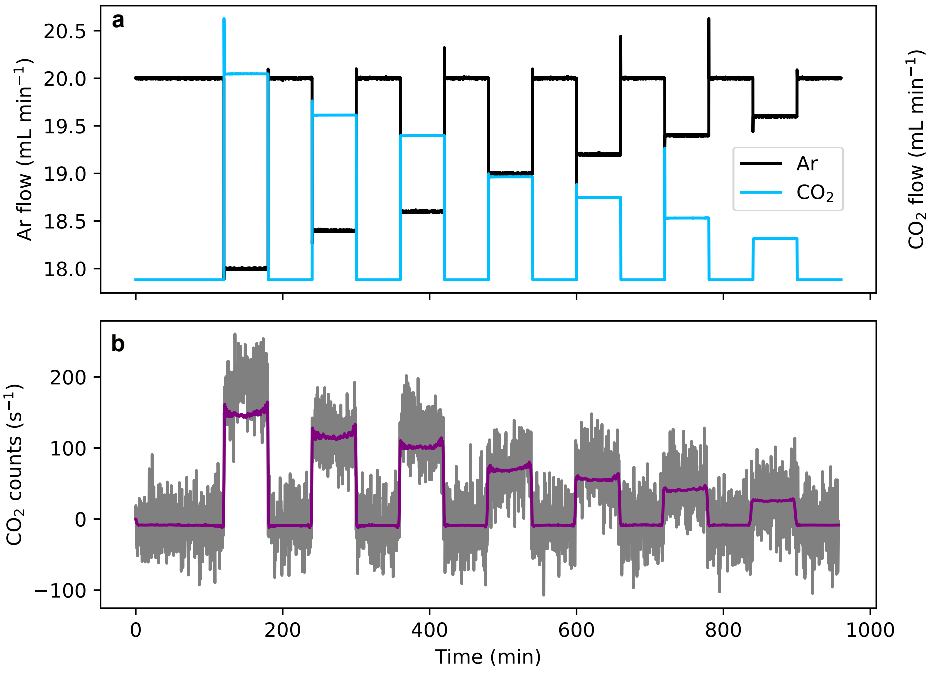

Supplementary Fig. 17 Validation of CO_2 Signal Origin and DAE Performance in a Non-Catalytic Nanofluidic Chip (a) Flow profile showing the introduction of discrete CO_2 pulses (diluted in Ar) through a nanofluidic chip with no Pd or catalytically active material. The pulse sequence spans a range of CO_2 concentrations, progressively decreasing to test the detection limit. (b) QMS signal at $m/z = 44$ after background subtraction (grey), overlaid with the output of the DAE-denoising algorithm (purple). Despite the low intensity of the raw signal at lower concentrations, the denoised trace successfully reconstructs the timing and shape of the CO_2 pulses. This demonstrates the robustness of the DAE approach for signal recovery in high-noise regimes.

Supplementary References

References

[1] Bengio, Y., Yao, L., Alain, G. & Vincent, P. Generalized denoising auto-encoders as generative models. *arXiv* (2013).

[2] Feng, X., Zhang, Y. & Glass, J. Speech feature denoising and dereverberation via deep autoencoders for noisy reverberant speech recognition. *2014 IEEE international conference on acoustics, speech and signal processing (ICASSP)* 1759–1763 (2014).

[3] Zhou, C. & Paffenroth, R. C. Anomaly detection with robust deep autoencoders. *Proceedings of the 23rd ACM SIGKDD international conference on knowledge discovery and data mining* 665–674 (2017).

[4] Valdes, A., Macwan, R. & Backes, M. Anomaly detection in electrical substation circuits via unsupervised machine learning. *2016 IEEE 17th International Conference on Information Reuse and Integration (IRI)* 500–505 (2016).

[5] Castellini, J., Poggioni, V. & Sorbi, G. Fake twitter followers detection by denoising autoencoder. *Proceedings of the International Conference on Web Intelligence* 195–202 (2017).

[6] Shen, H., George, D., Huerta, E. A. & Zhao, Z. Denoising gravitational waves with enhanced deep recurrent denoising auto-encoders. *ICASSP 2019 - 2019 IEEE International Conference on Acoustics, Speech and Signal Processing (ICASSP)* 3237–3241 (2019).

[7] Shouran, M. & Elgamli, E. Design and implementation of butterworth filter. *Int. J. Innov. Res. Sci. Eng. Technol.* **9**, 7975 (2020).

[8] Fahmy, M. & Hasan, Y. Signal denoising using hankel matrix rank reduction. *Proceedings of the Twenty-First National Radio Science Conference, 2004.* C36–1 (2004).

[9] Condat, L. A direct algorithm for 1-D total variation denoising. *IEEE Signal Processing Letters* **20**, 1054–1057 (2013).

[10] Li, N., Zhang, J. & Deng, Z. Optimization of wavelet threshold denoising based on edge detection . *Ninth International Conference on Digital Image Processing (ICDIP 2017)* **10420**, 104200O (2017).

[11] Zhang, X. & Jiang, S. Application of fourier transform and butterworth filter in signal denoising. *Biomedical Optics Express* 1277–1281 (2021).

[12] Tawfik, M. S. *et al.* Comparative study of traditional and deep-learning denoising approaches for image-based petrophysical characterization of porous media.

Front. Water **3** (2022).

[13] Proakis, J. G. & Manolakis, D. G. *Digital signal processing - principles, algorithms and applications (2. ed.).* (1992).

[14] Korhonen, J. & You, J. Peak signal-to-noise ratio revisited: is simple beautiful? *2012 Fourth International Workshop on Quality of Multimedia Experience* 37–38 (2012).

[15] Bishop, C. M. *Pattern recognition and machine learning.*, Ch. 3, Linear Models for Regression, 137–211 (Springer, 2006).

[16] Bengio, Y., Louradour, J., Collobert, R. & Weston, J. *Curriculum learning.*, ICML '09, 41–48 (Association for Computing Machinery, New York, NY, USA, 2009).

[17] Ida, N. *Sensors, actuators, and their interfaces: a multidisciplinary introduction.* (2014).

[18] Siemens, C. W. The bakerian lecture: on the increase of electrical resistance in conductors with rise of temperature, and its application to the measure of ordinary and furnace temperatures; also on a simple method of measuring electrical resistances. [abstract]. *Proceedings of the Royal Society of London* **19**, 443–445 (1870).

[19] Dusen, M. S. V. Platinum-resistance thermometry at low temperatures. *J. Am. Chem. Soc.* **47**, 326–332 (1925).
