# Supplementary figures and images for: Deep-learning-enabled online mass spectrometry of the reaction product of a single catalyst nanoparticle
Source: Nat Commun. 2025 Aug 5;16:7203. doi: 10.1038/s41467-025-62602-3 (PMC12325981; doi:10.1038/s41467-025-62602-3)

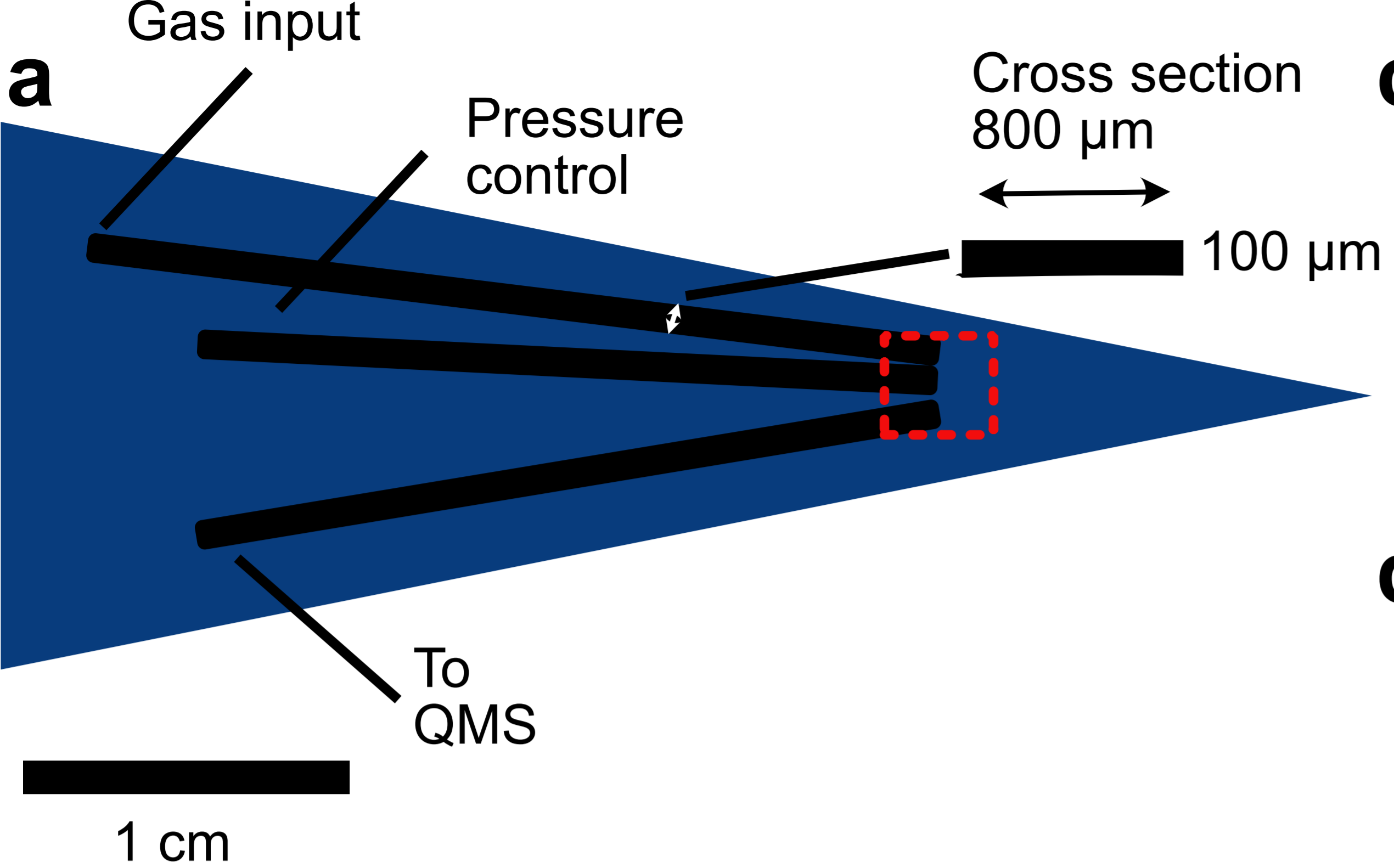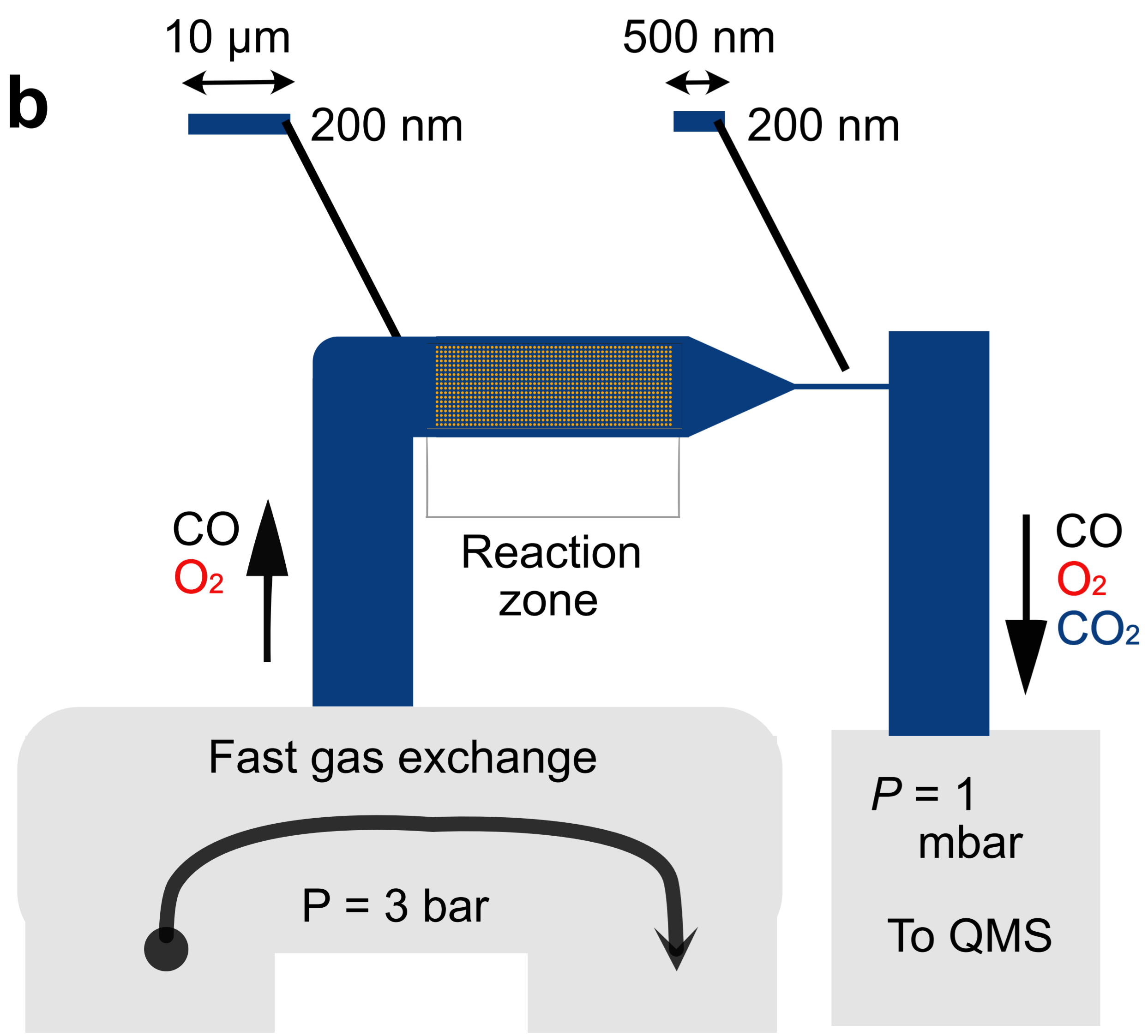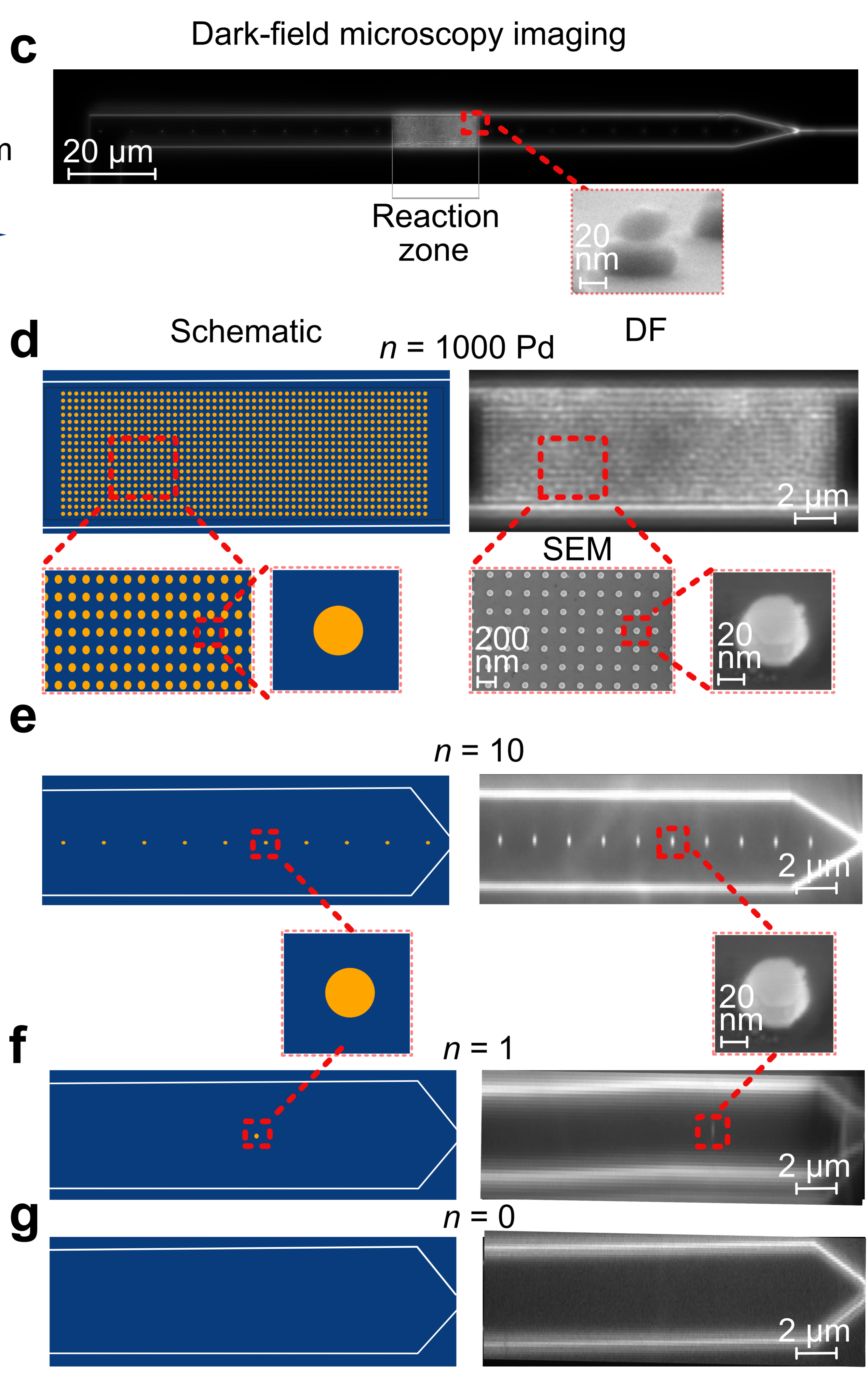

Supplement: Supplementary file 2 — LaTeX Supplementary File [file 41467_2025_62602_MOESM2_ESM.zip › Figures/Article_Fig1.pdf]

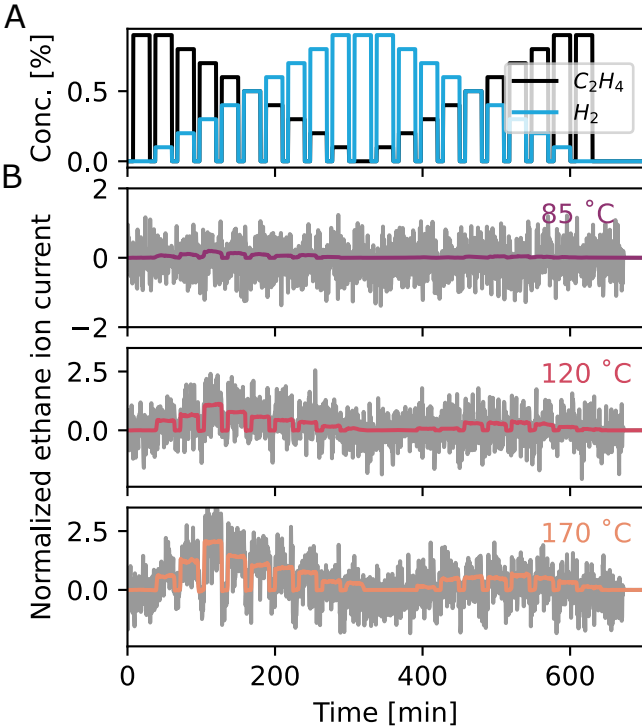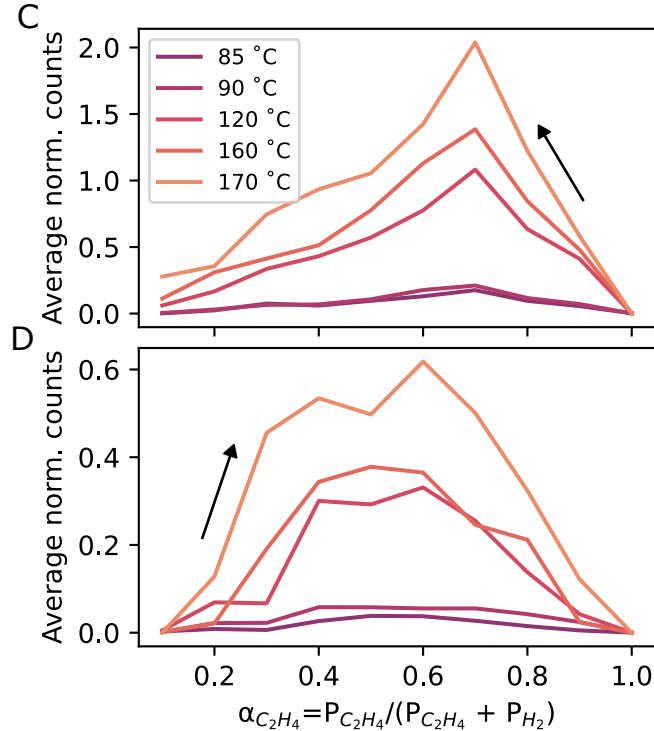

Supplement: Supplementary file 2 — LaTeX Supplementary File [file 41467_2025_62602_MOESM2_ESM.zip › Figures/Article_Fig6.pdf]

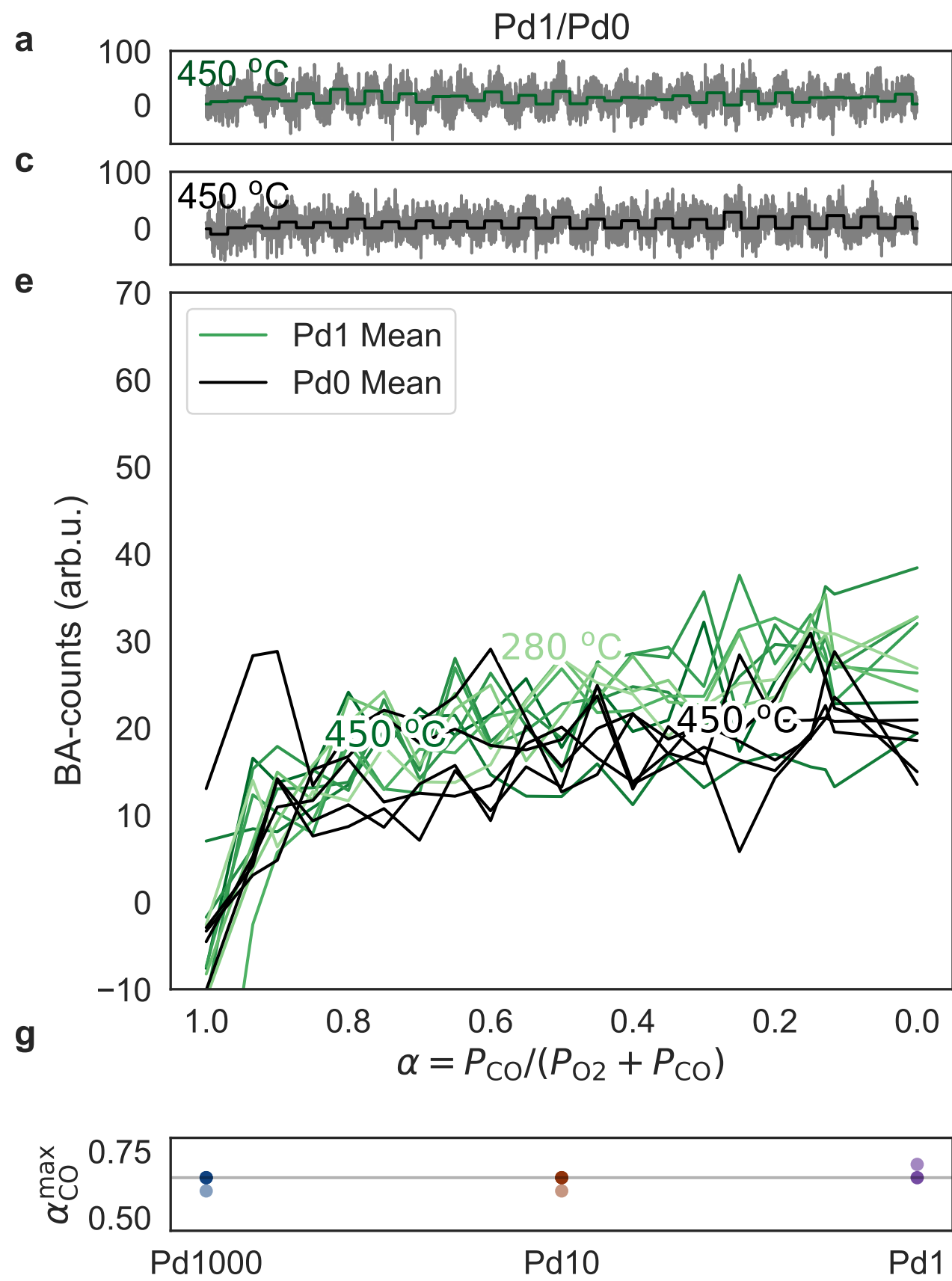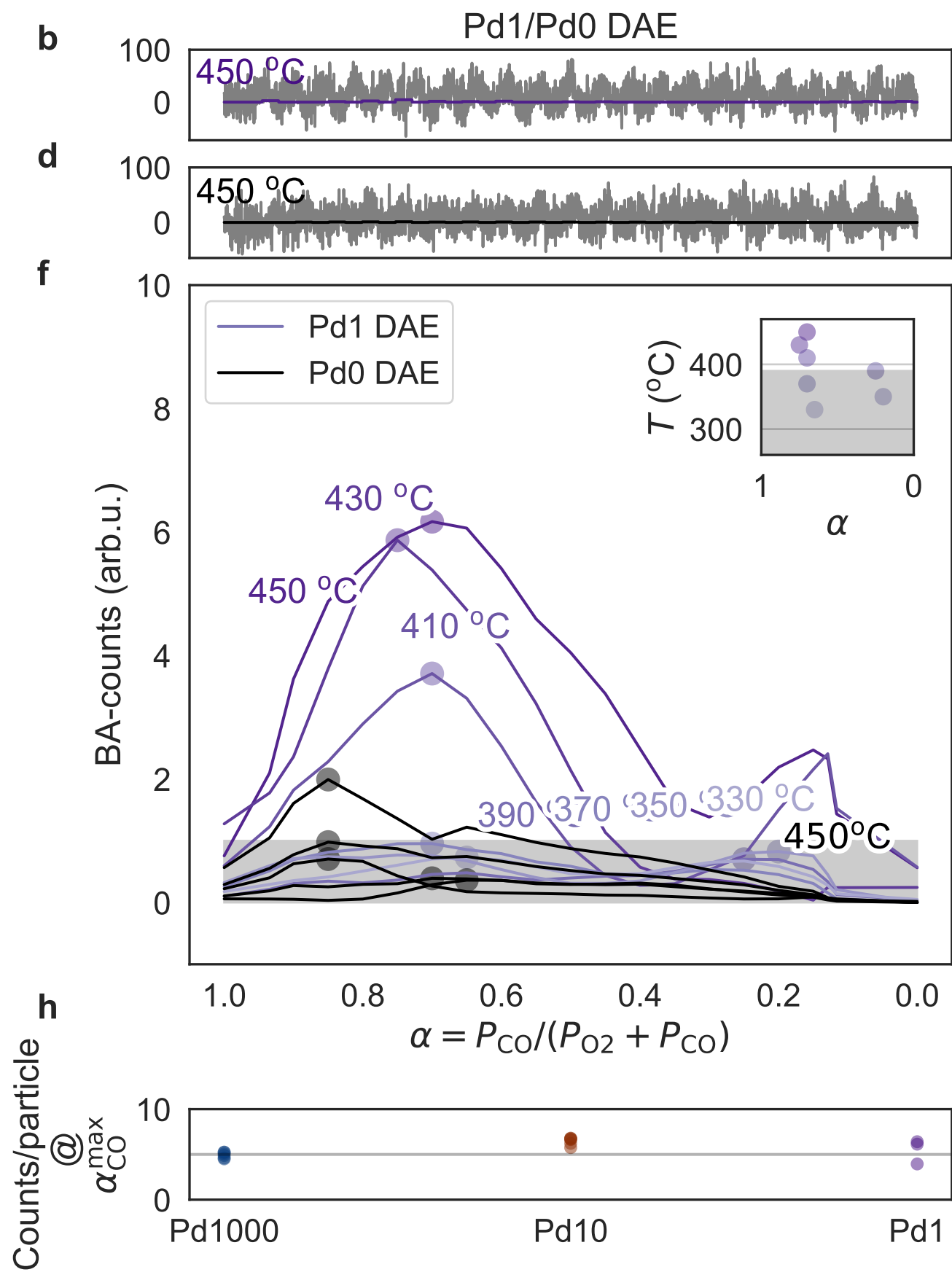

Supplement: Supplementary file 2 — LaTeX Supplementary File [file 41467_2025_62602_MOESM2_ESM.zip › Figures/Article_Fig5.pdf]

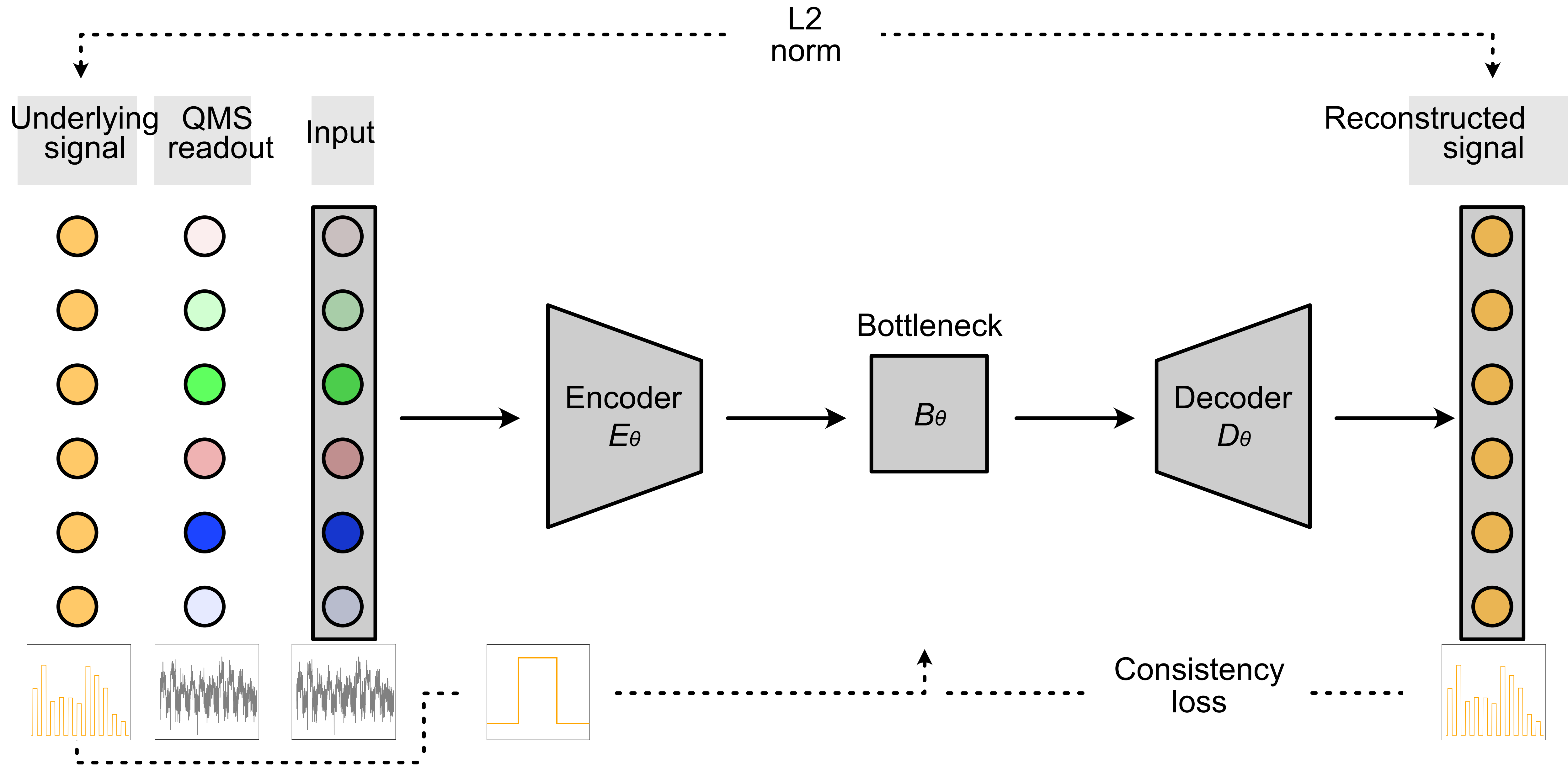

Supplement: Supplementary file 2 — LaTeX Supplementary File [file 41467_2025_62602_MOESM2_ESM.zip › Figures/Article_Fig3.pdf]

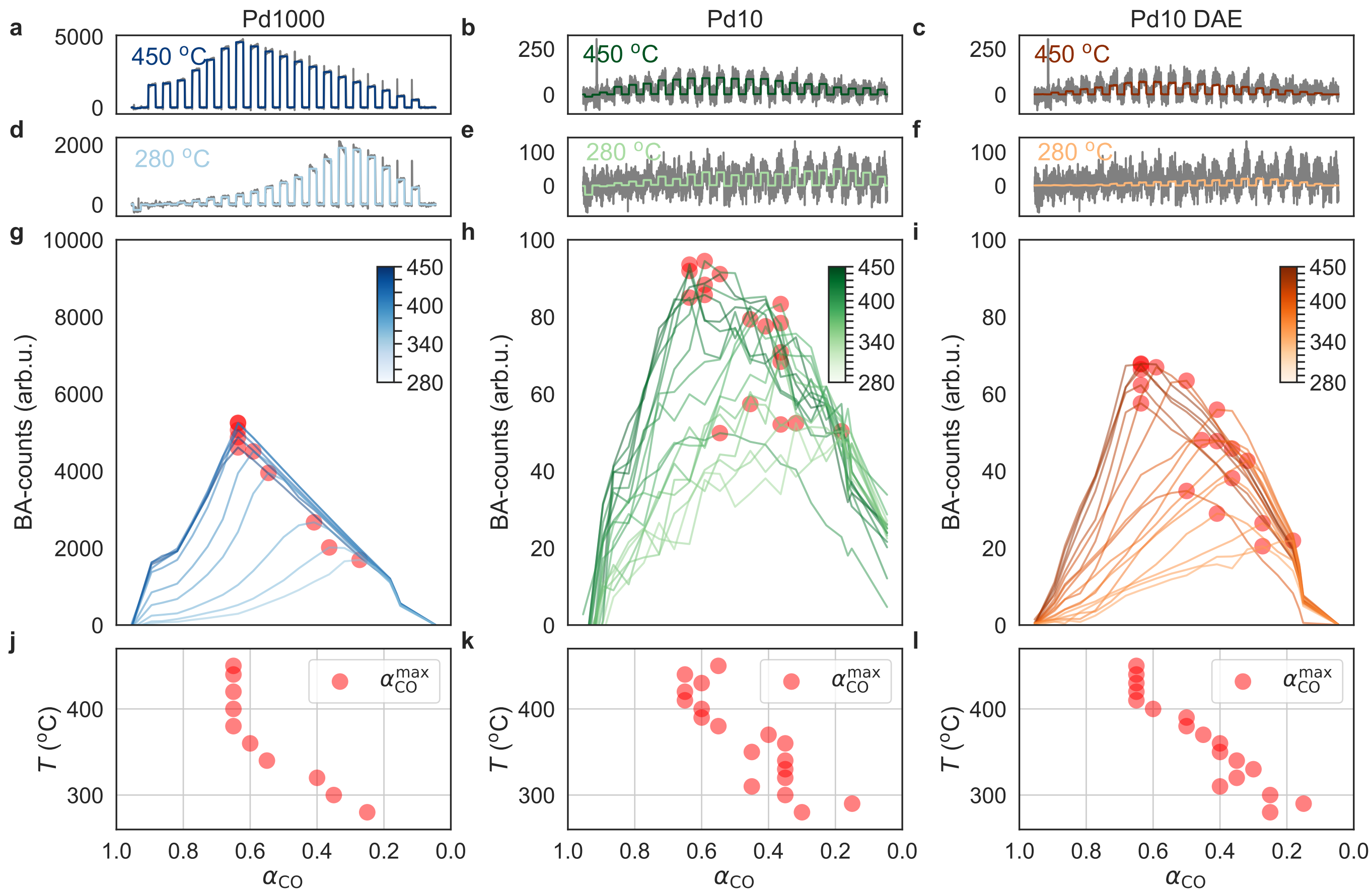

Supplement: Supplementary file 2 — LaTeX Supplementary File [file 41467_2025_62602_MOESM2_ESM.zip › Figures/Article_Fig4.pdf]

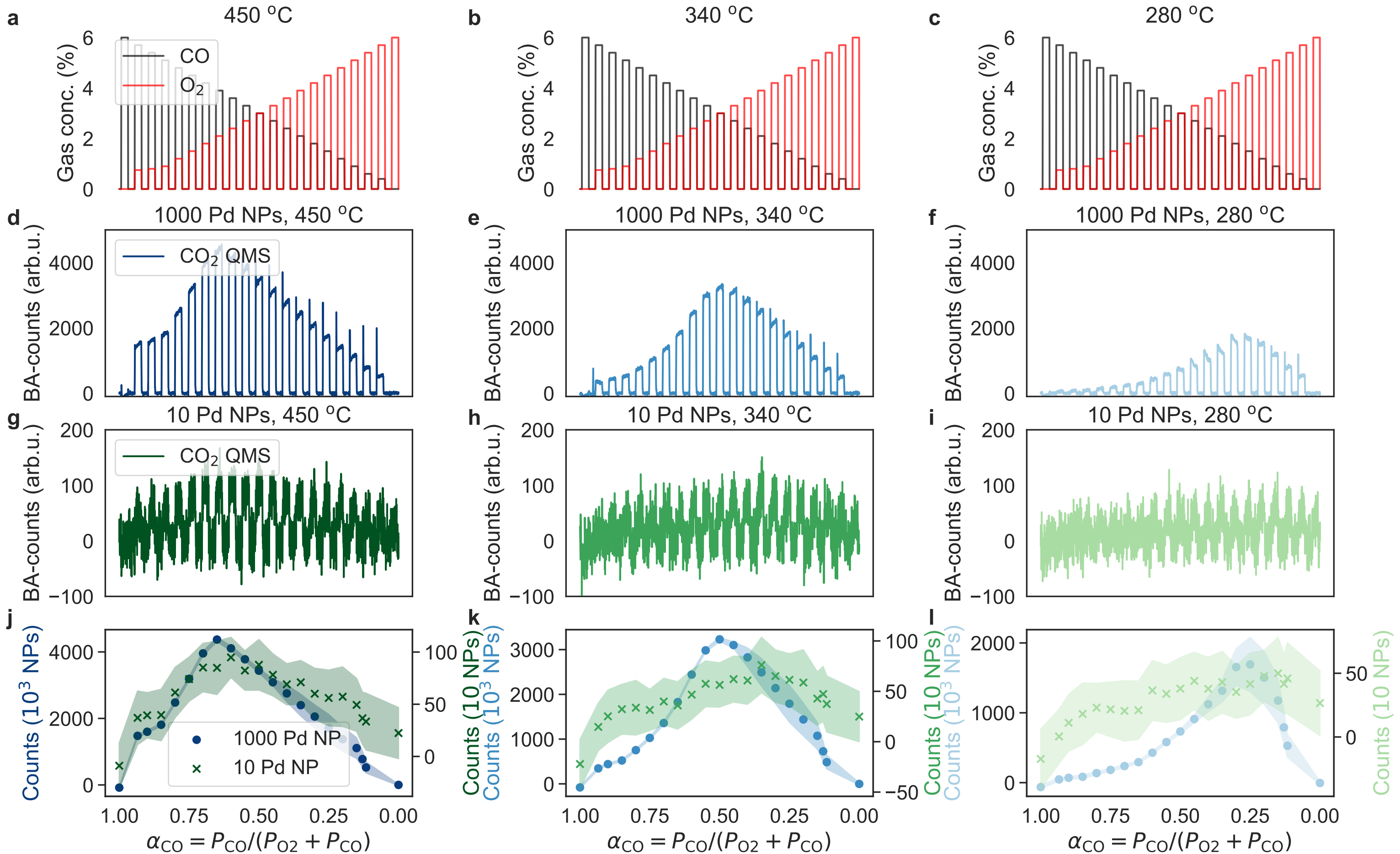

Supplement: Supplementary file 2 — LaTeX Supplementary File [file 41467_2025_62602_MOESM2_ESM.zip › Figures/Article_Fig2.pdf]

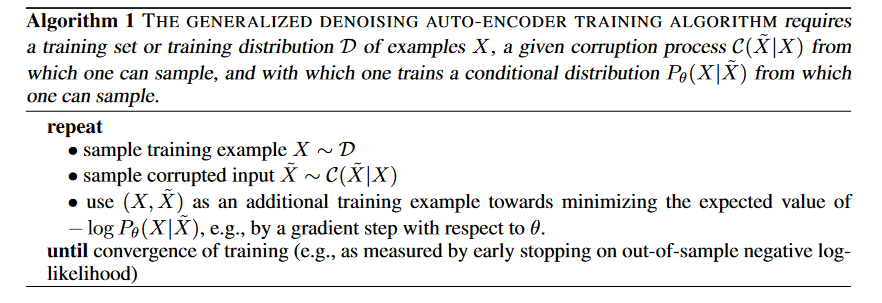

Supplement: Supplementary file 2 — LaTeX Supplementary File [file 41467_2025_62602_MOESM2_ESM.zip › SI_Figures/DAEAlgorithm.PNG]

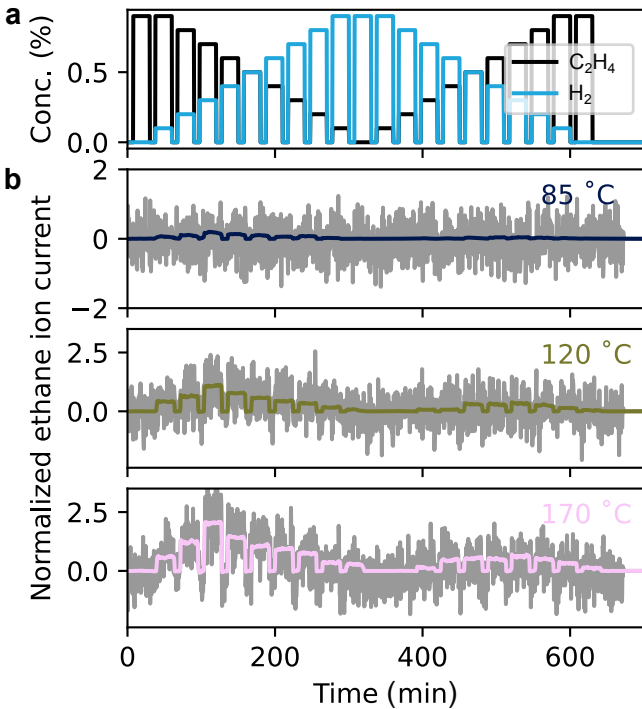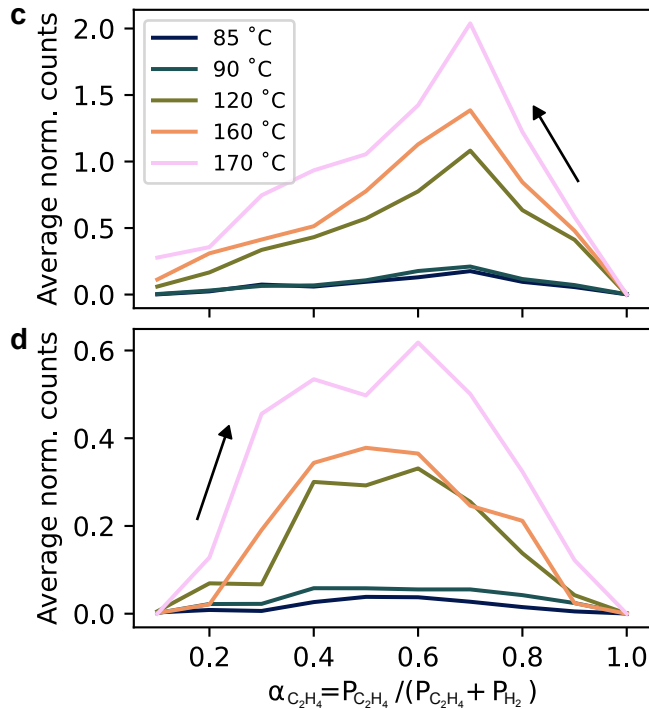

Supplement: Supplementary file 2 — LaTeX Supplementary File [file 41467_2025_62602_MOESM2_ESM.zip › Figures/Article_Fig6_revised.pdf]

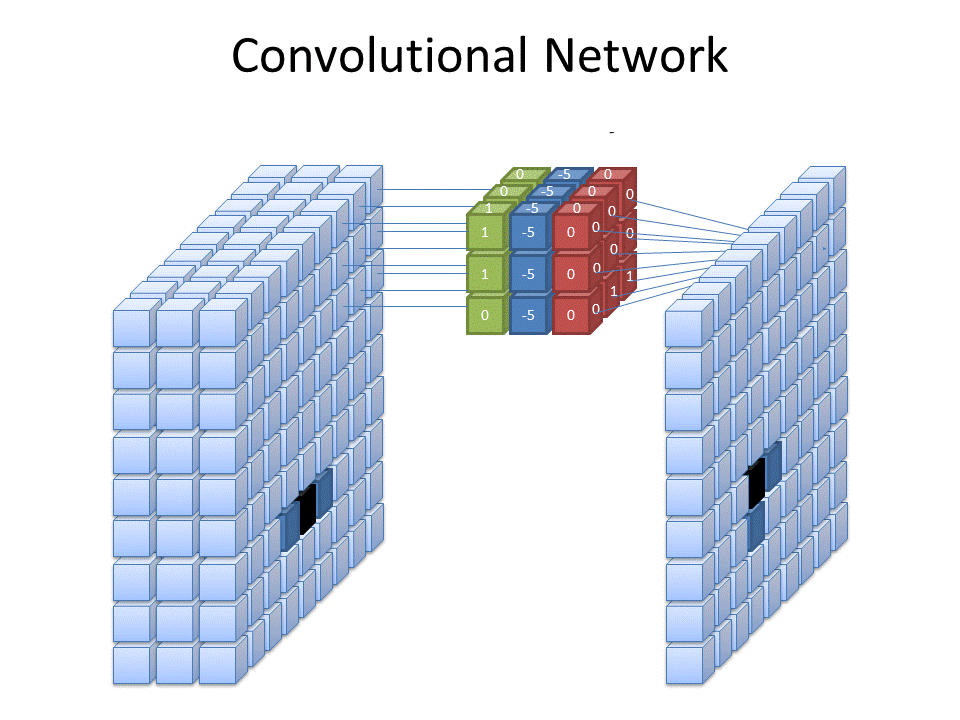

Supplement: Supplementary file 2 — LaTeX Supplementary File [file 41467_2025_62602_MOESM2_ESM.zip › SI_Figures/fig_07_01.png]

**Input**

**Output**

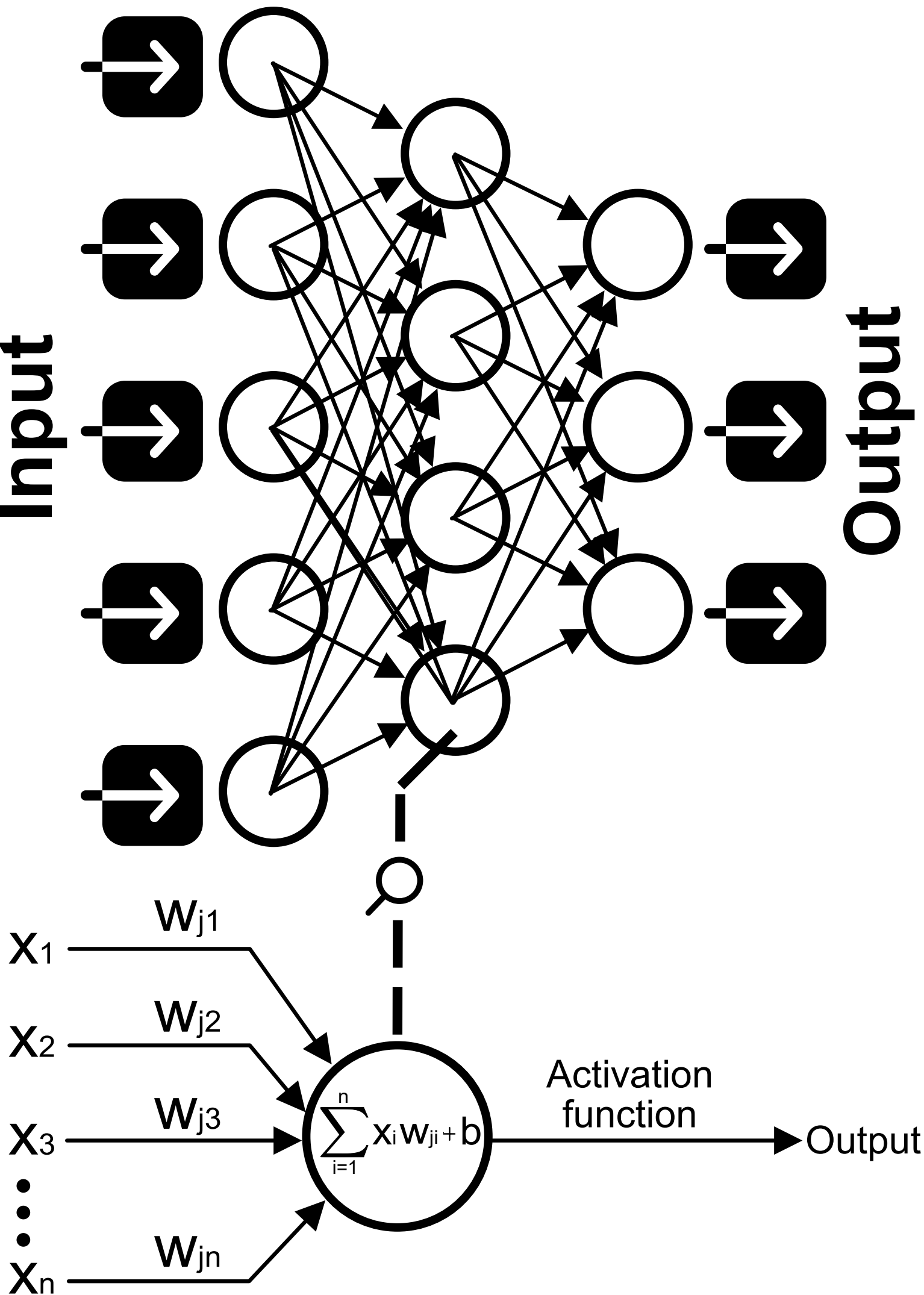

Supplement: Supplementary file 2 — LaTeX Supplementary File [file 41467_2025_62602_MOESM2_ESM.zip › SI_Figures/fig_07_02.pdf]

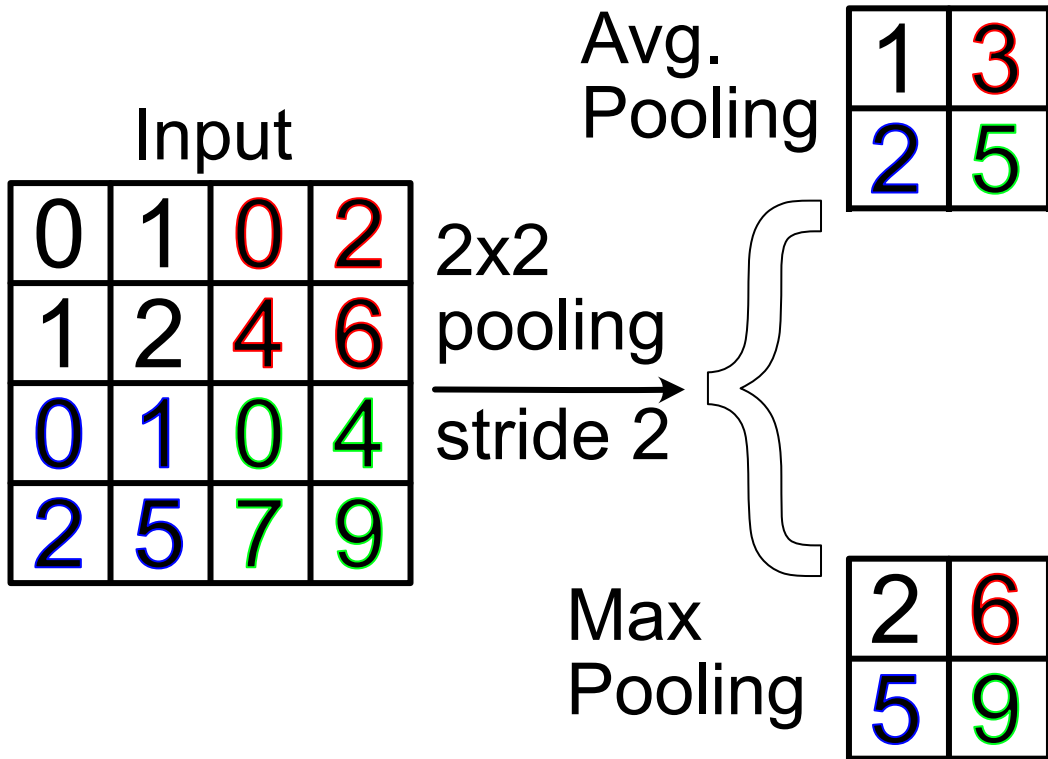

Supplement: Supplementary file 2 — LaTeX Supplementary File [file 41467_2025_62602_MOESM2_ESM.zip › SI_Figures/fig_07_04.pdf]

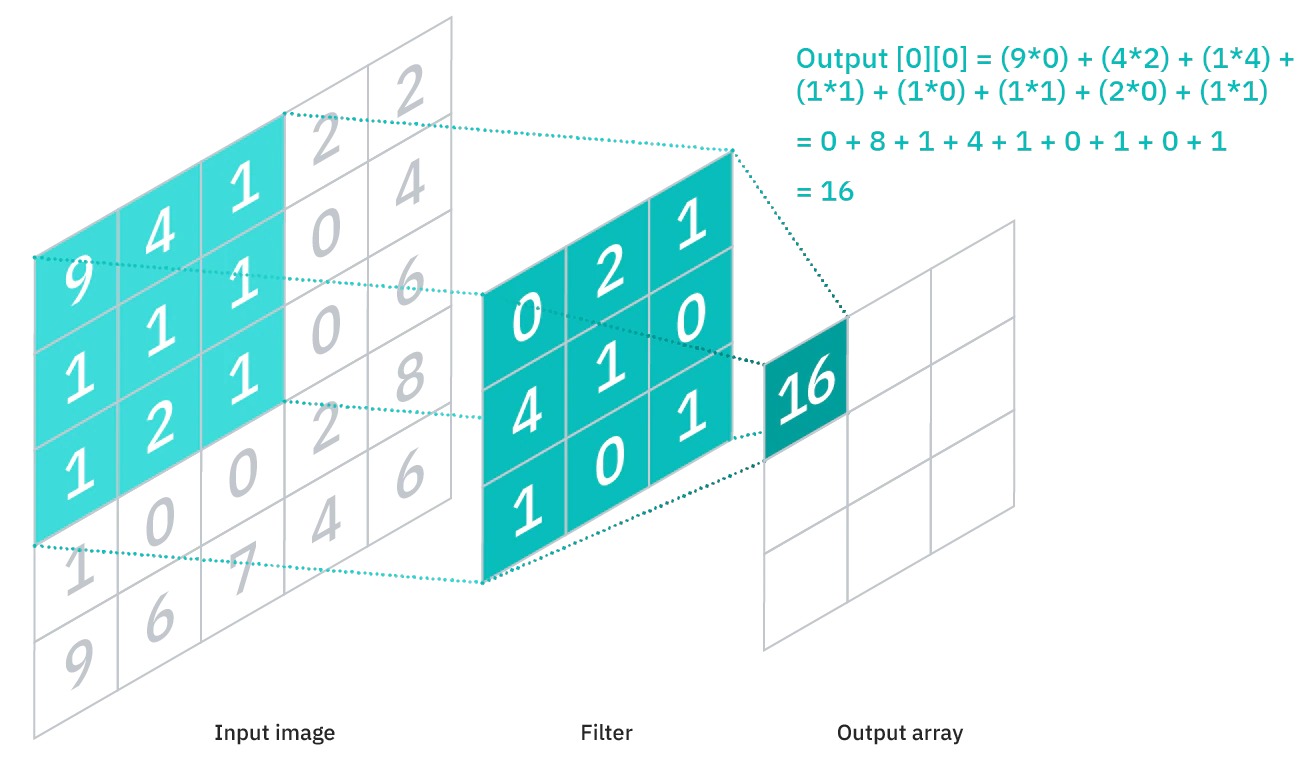

Supplement: Supplementary file 2 — LaTeX Supplementary File [file 41467_2025_62602_MOESM2_ESM.zip › SI_Figures/fig_07_03.png]

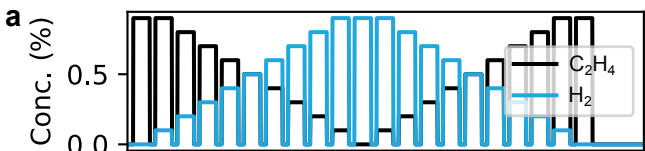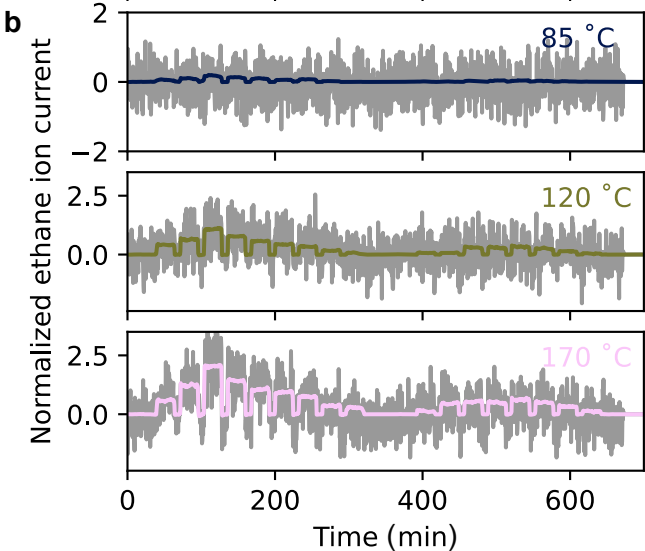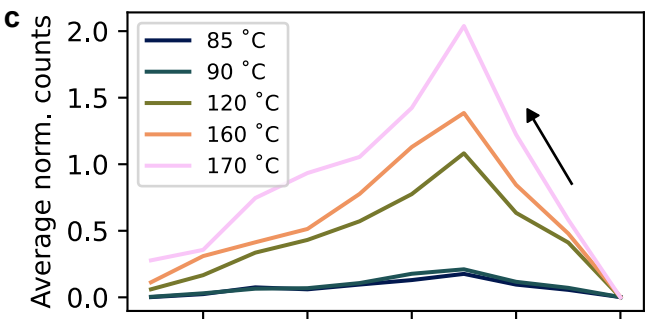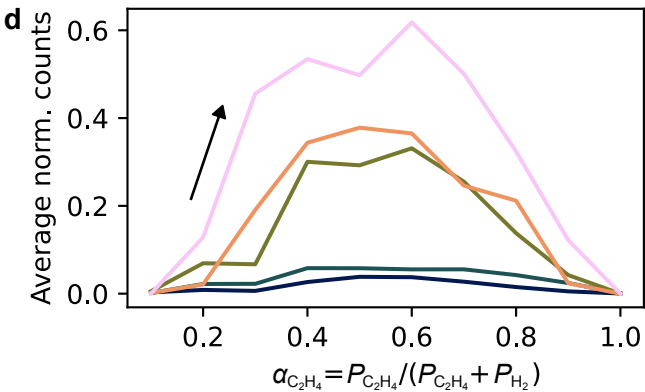

Supplement: Supplementary file 2 — LaTeX Supplementary File [file 41467_2025_62602_MOESM2_ESM.zip › Figures/Article_Fig6_reshaped.pdf]

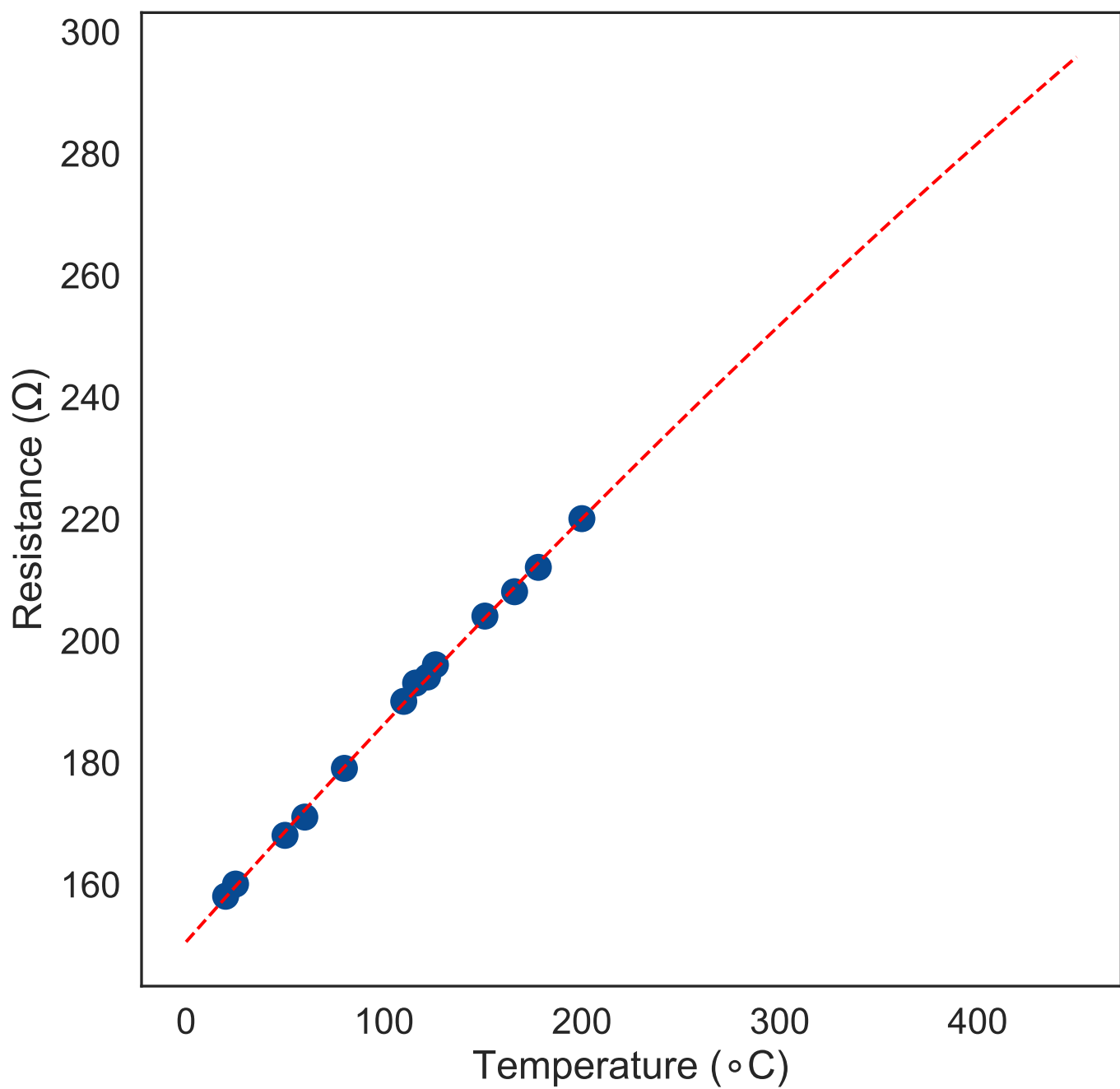

Supplement: Supplementary file 2 — LaTeX Supplementary File [file 41467_2025_62602_MOESM2_ESM.zip › SI_Figures/HeatFit.pdf]

$n = 10$

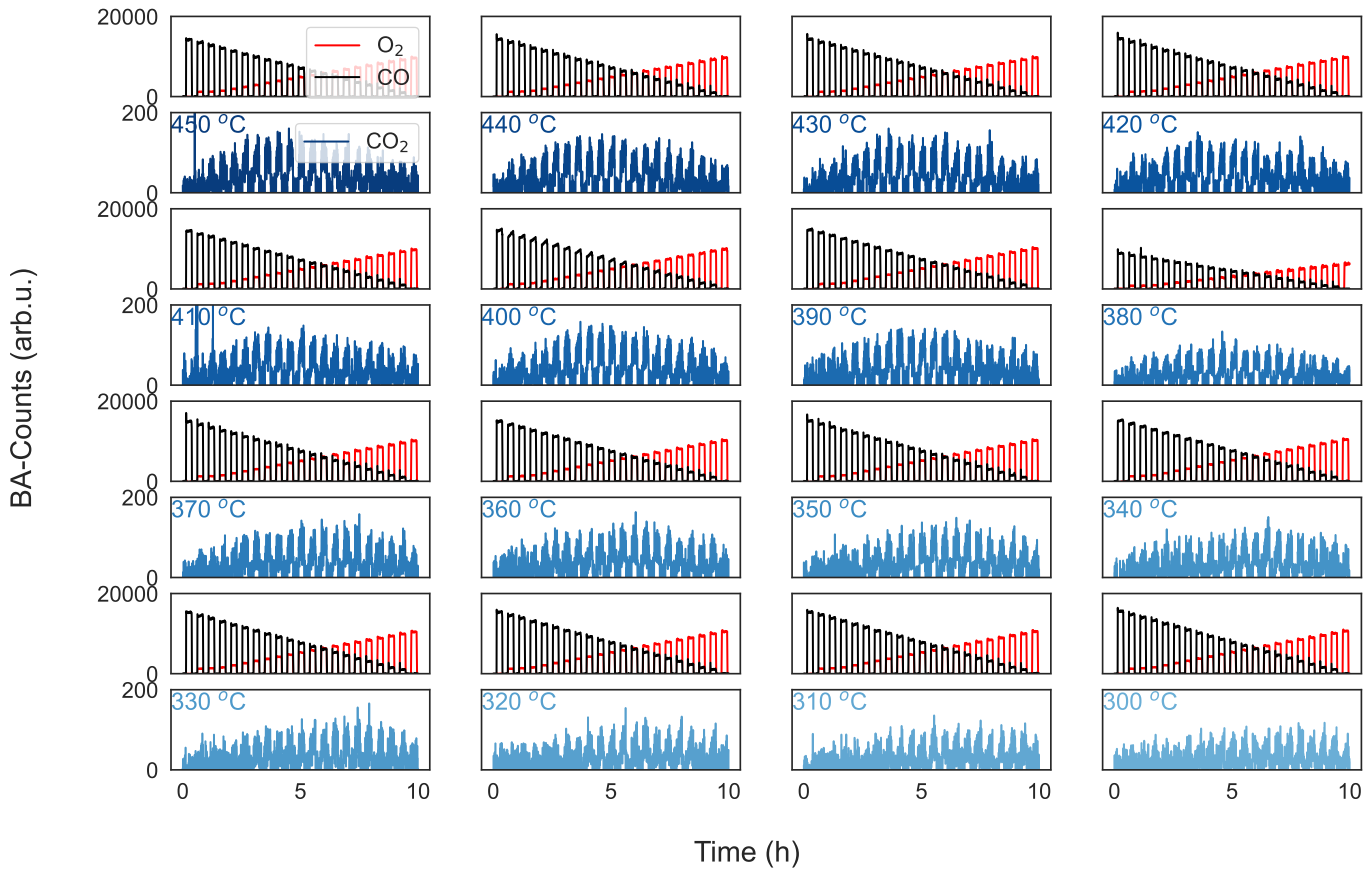

Supplement: Supplementary file 2 — LaTeX Supplementary File [file 41467_2025_62602_MOESM2_ESM.zip › SI_Figures/QMSCombined10.pdf]

$n = 1000$

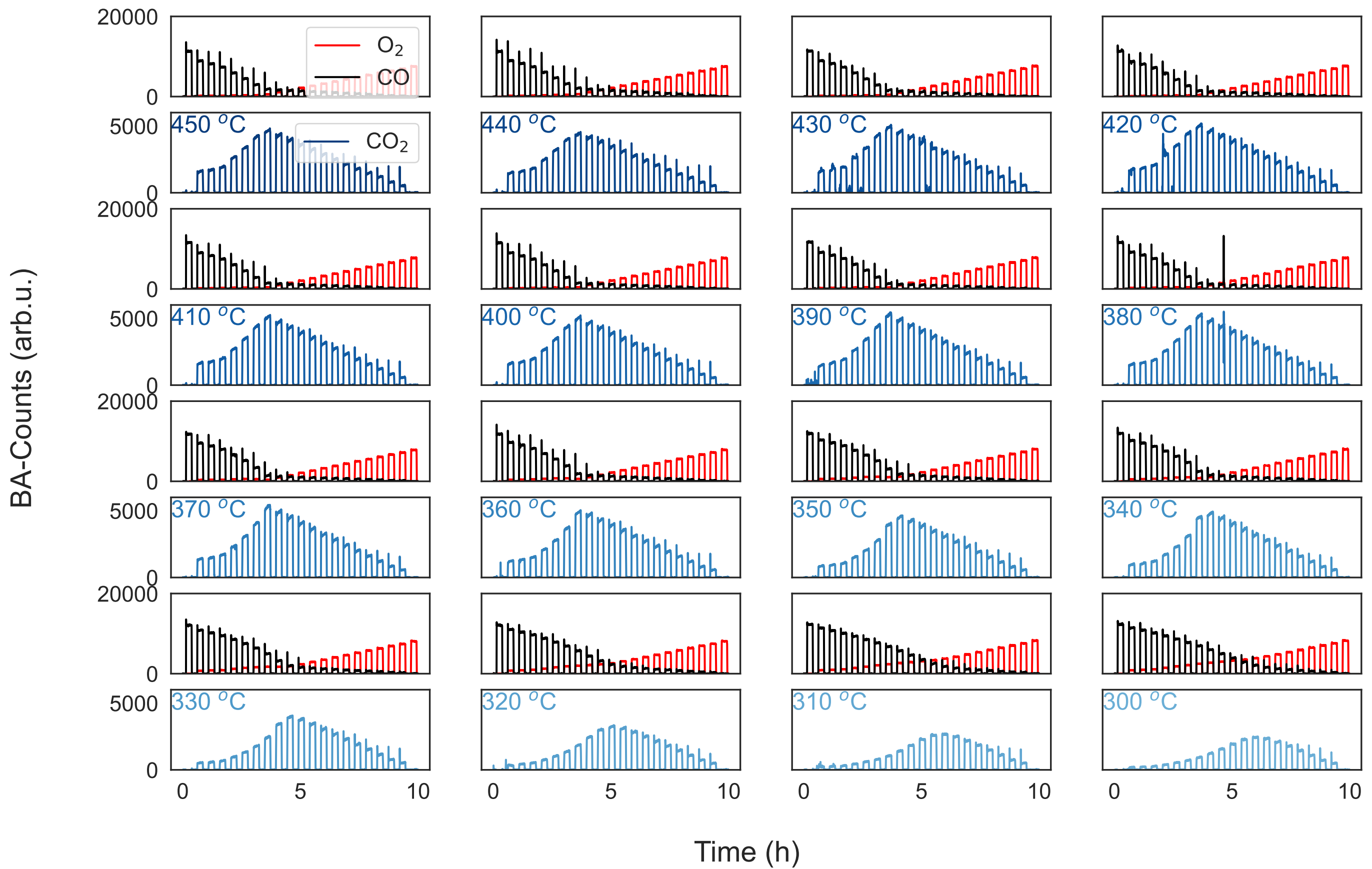

Supplement: Supplementary file 2 — LaTeX Supplementary File [file 41467_2025_62602_MOESM2_ESM.zip › SI_Figures/QMSCombined1000.pdf]

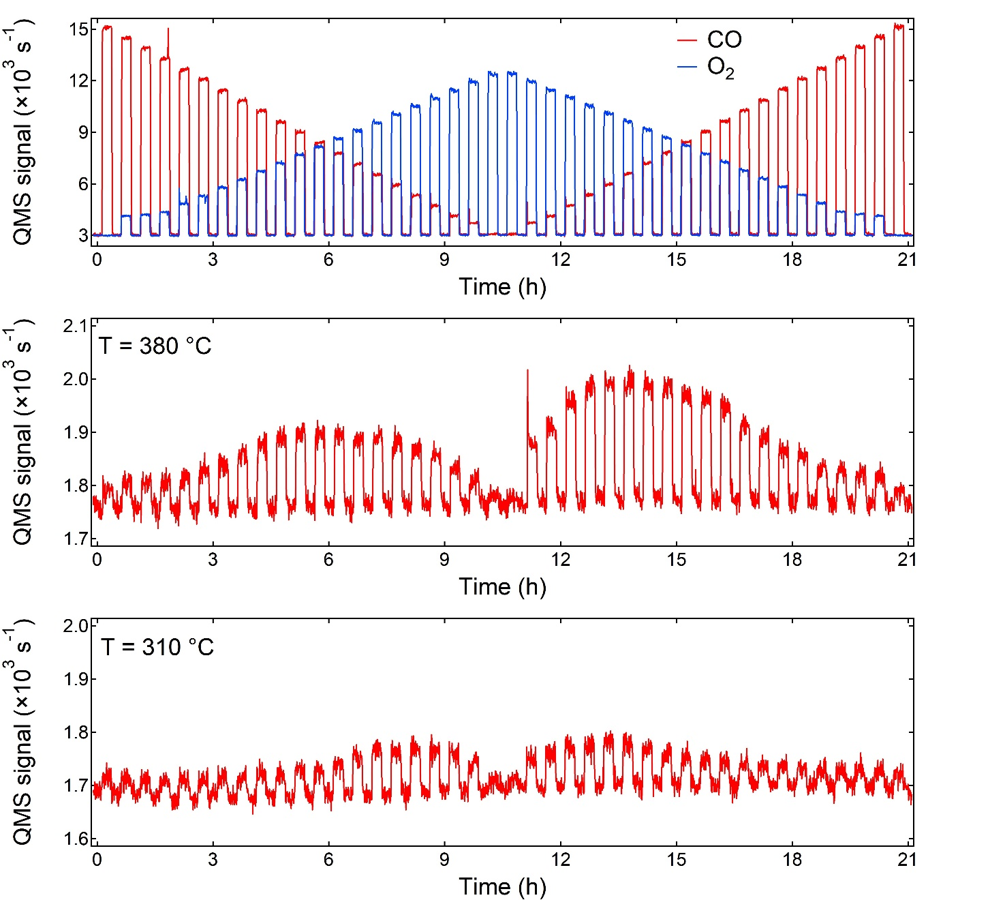

Supplement: Supplementary file 2 — LaTeX Supplementary File [file 41467_2025_62602_MOESM2_ESM.zip › SI_Figures/QMSCombined.png]

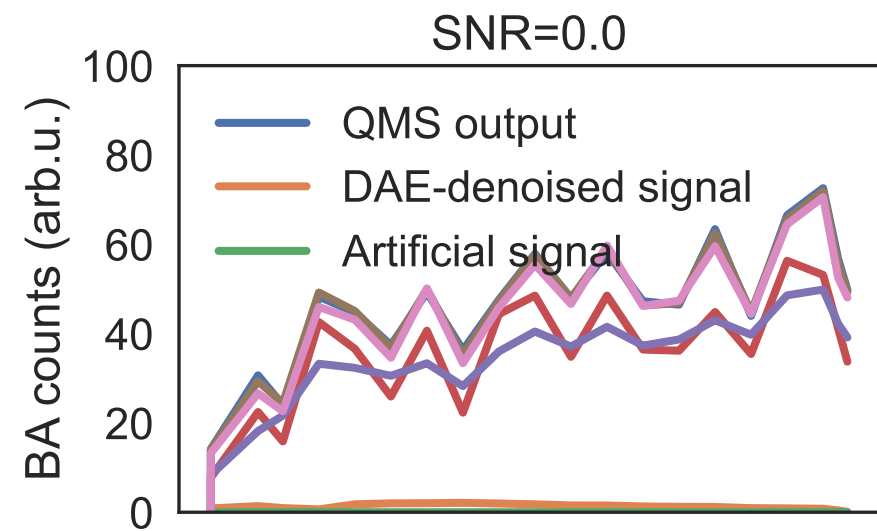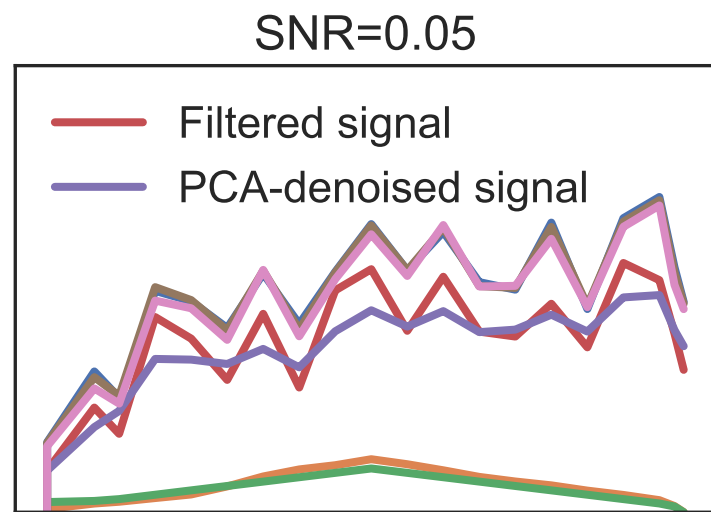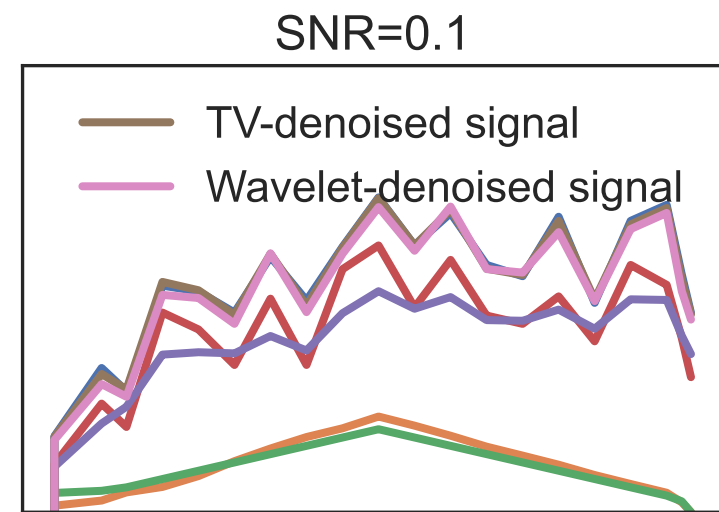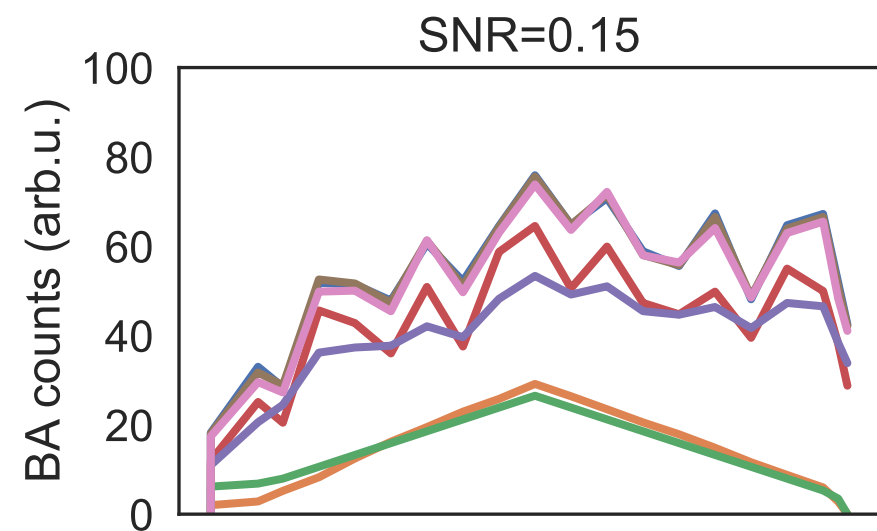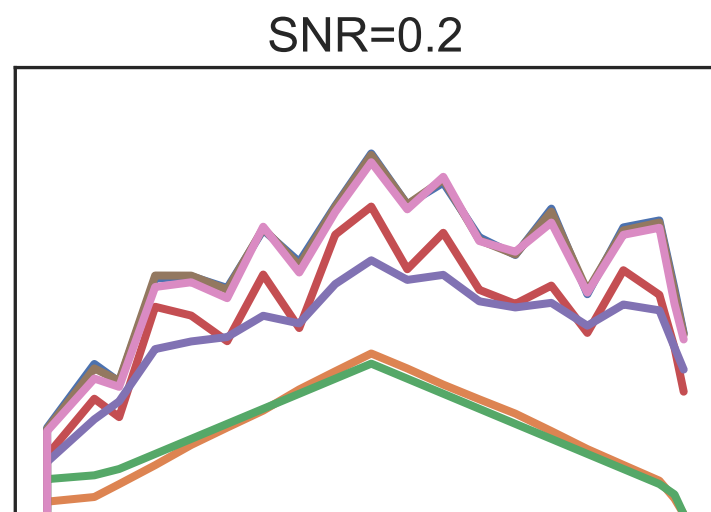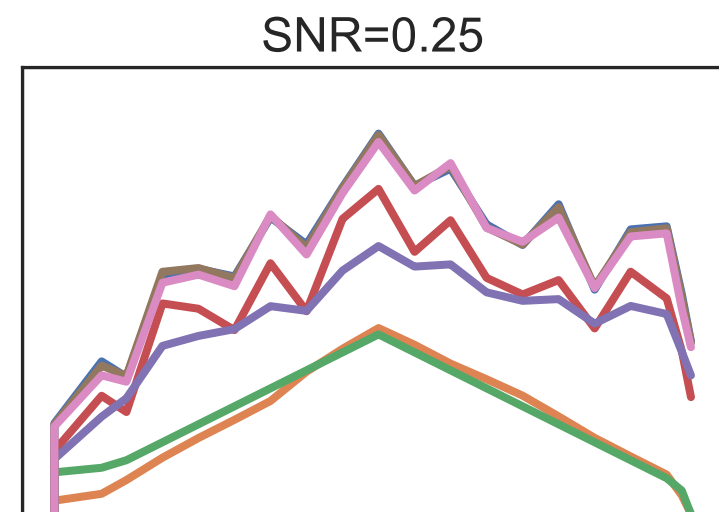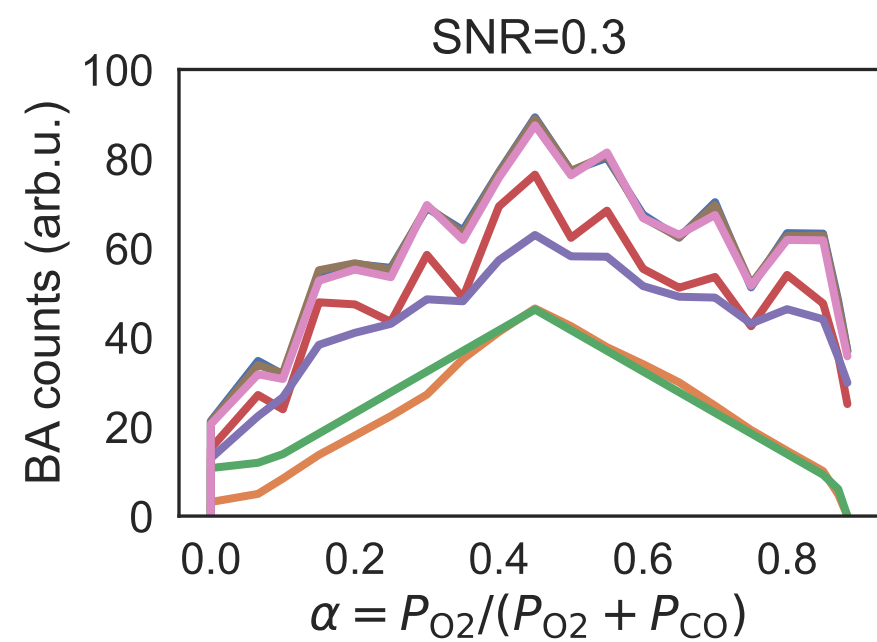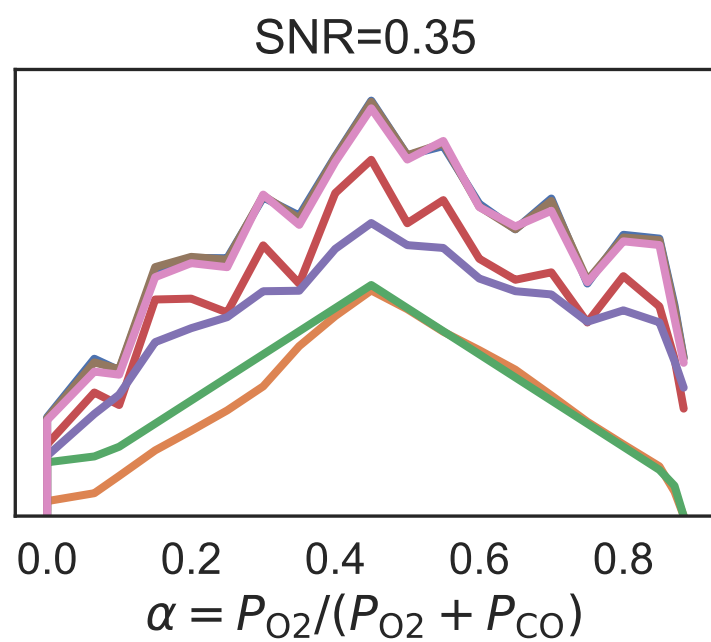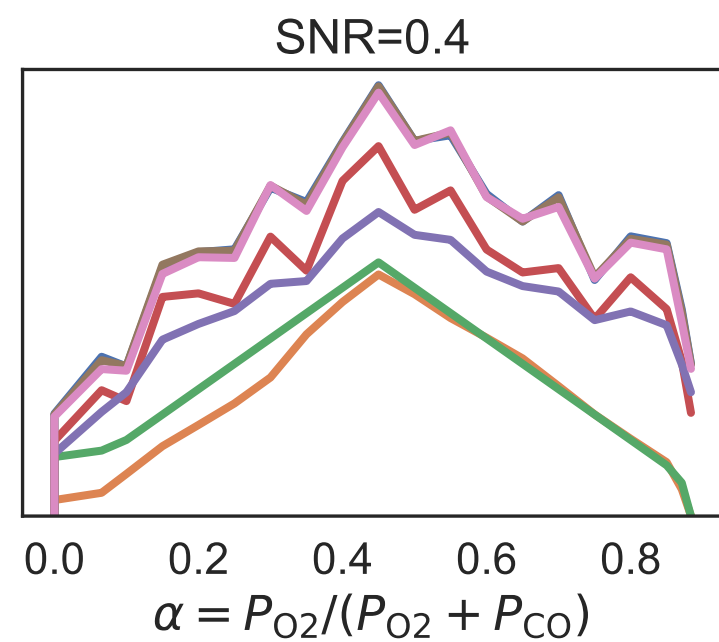

Supplement: Supplementary file 2 — LaTeX Supplementary File [file 41467_2025_62602_MOESM2_ESM.zip › SI_Figures/SI_Comp2.pdf]

SNR=0.1

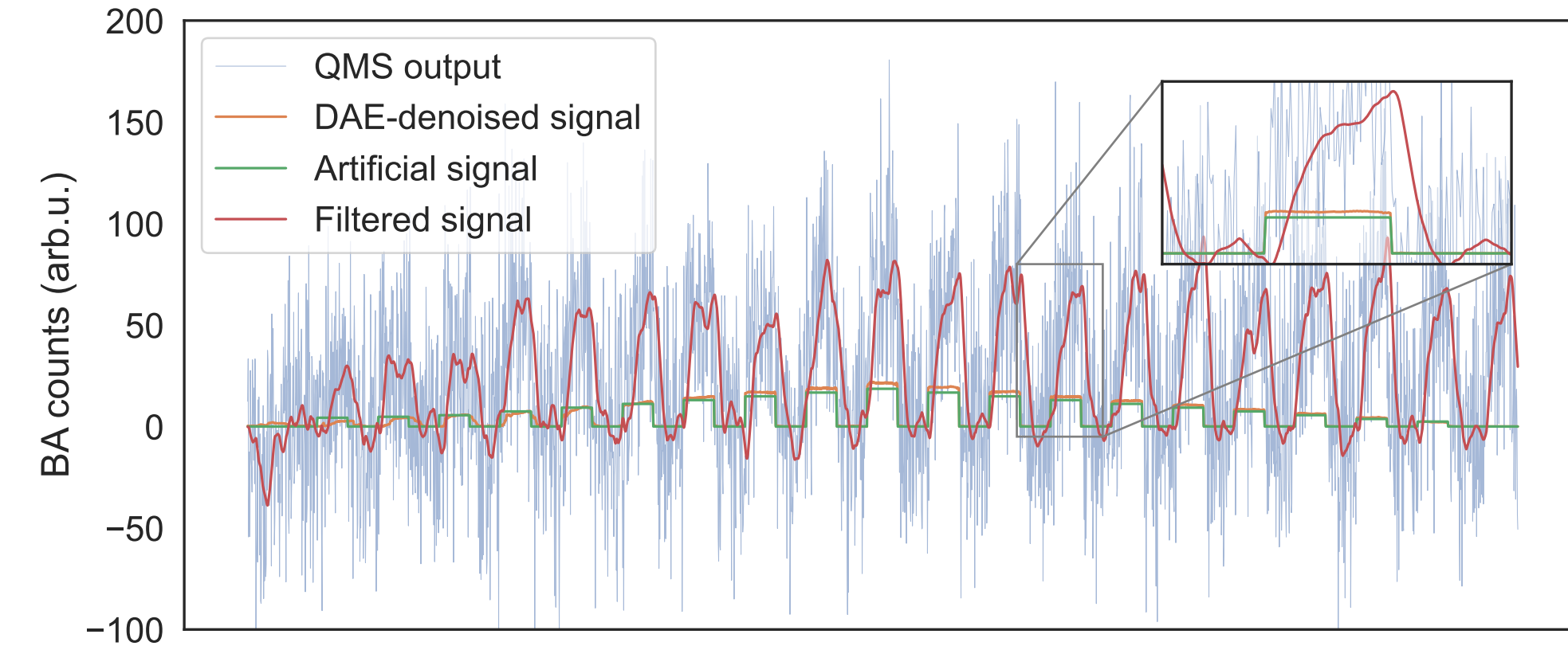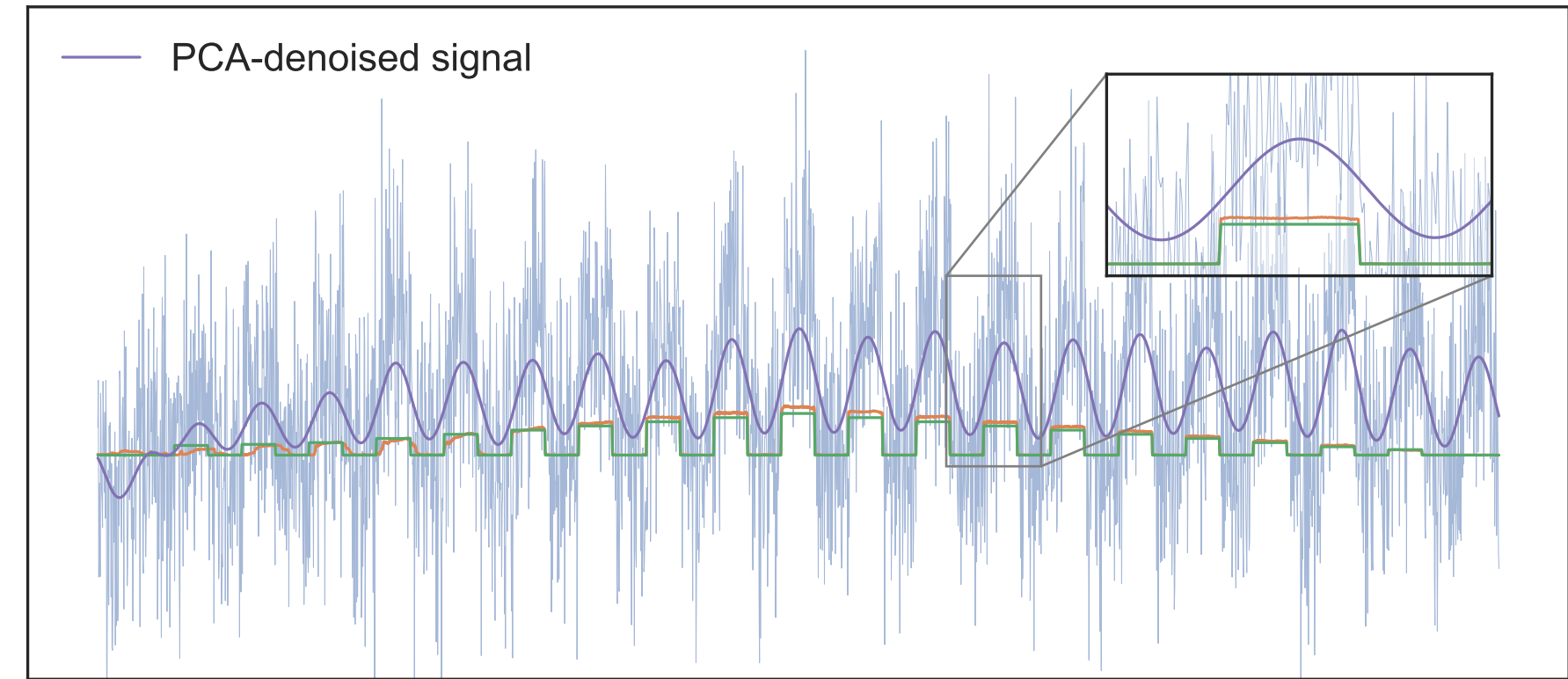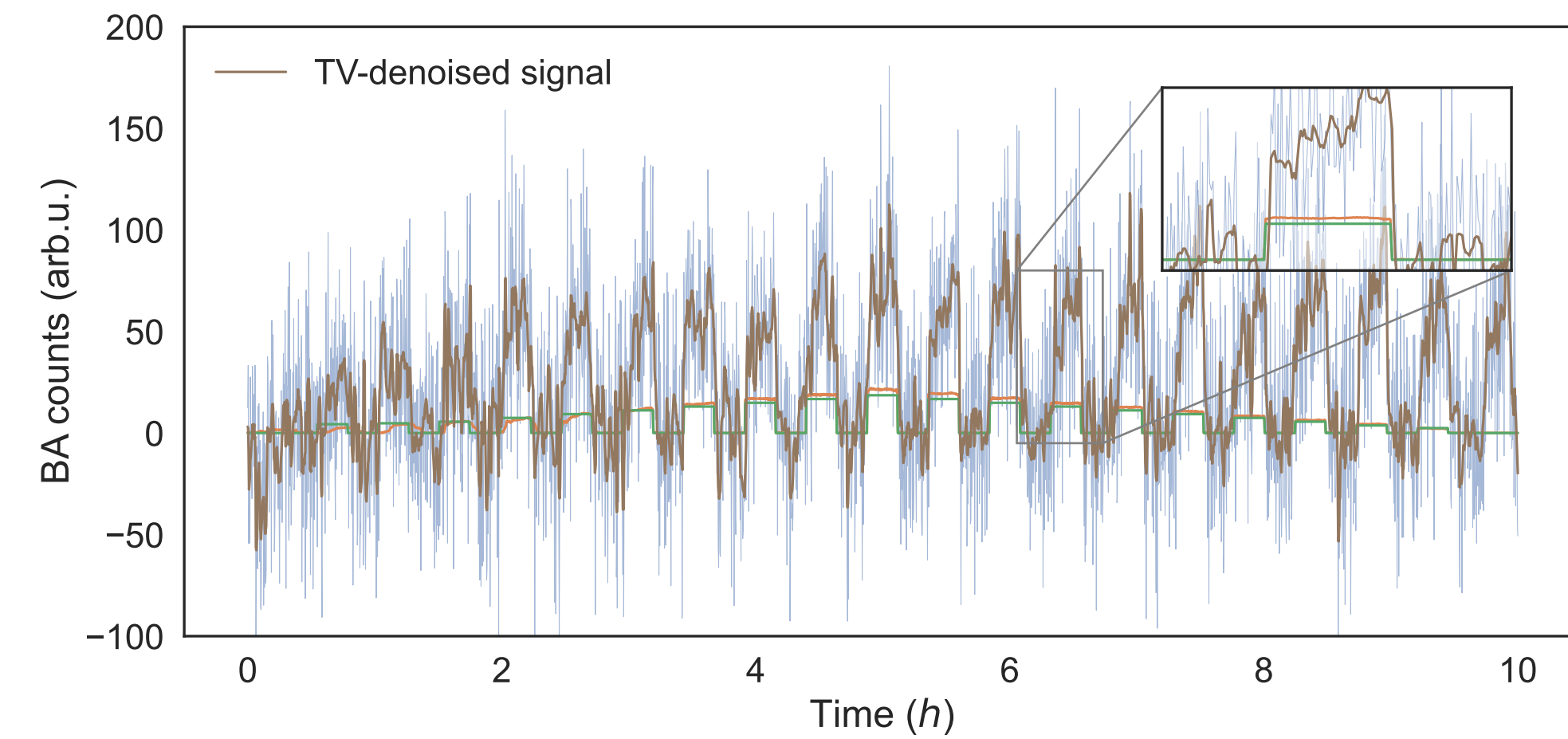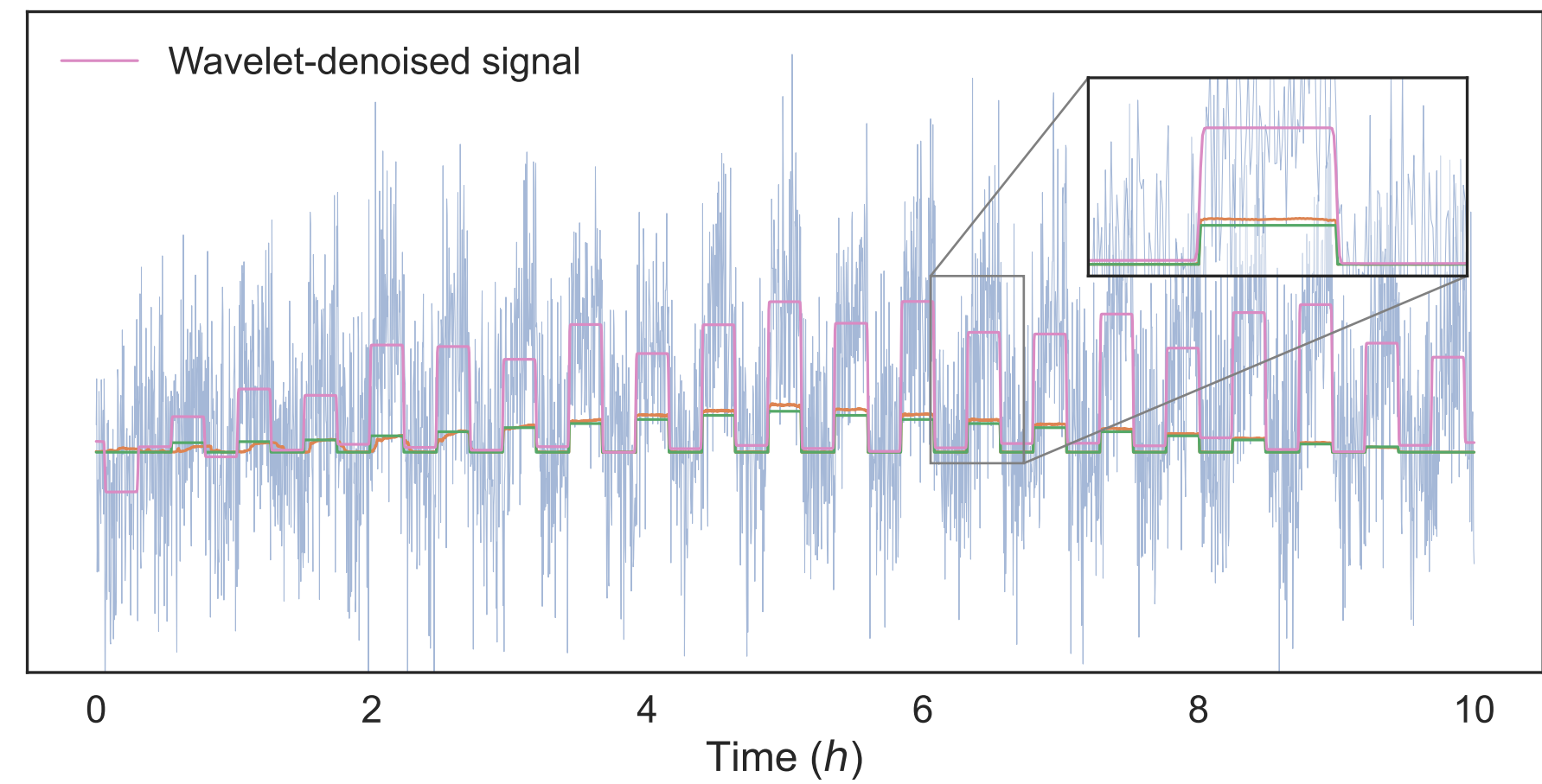

Supplement: Supplementary file 2 — LaTeX Supplementary File [file 41467_2025_62602_MOESM2_ESM.zip › SI_Figures/SI_Comp1.pdf]

$n = 1$

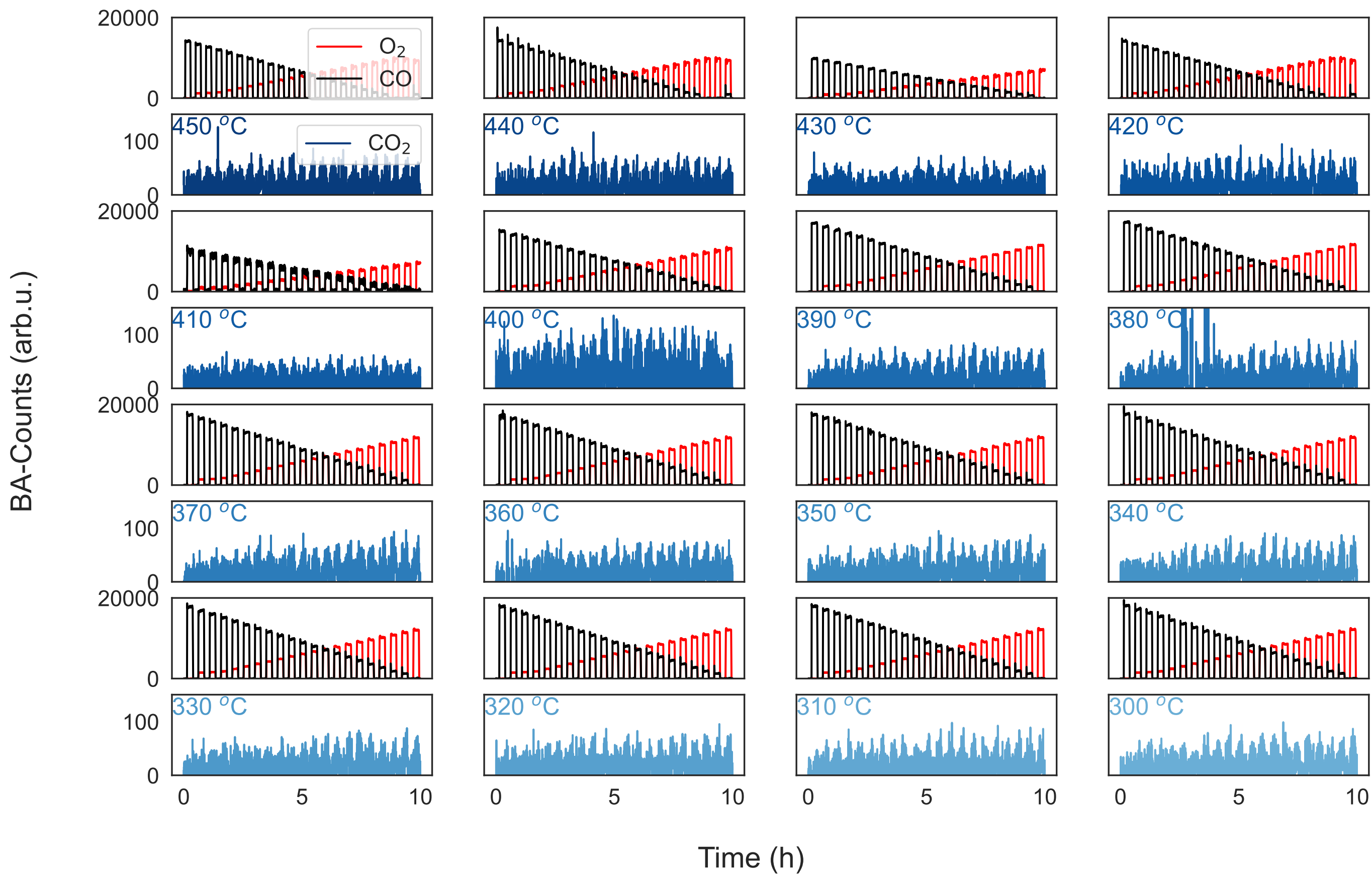

Supplement: Supplementary file 2 — LaTeX Supplementary File [file 41467_2025_62602_MOESM2_ESM.zip › SI_Figures/QMSCombined1.pdf]

Comparative analysis of denoising methods

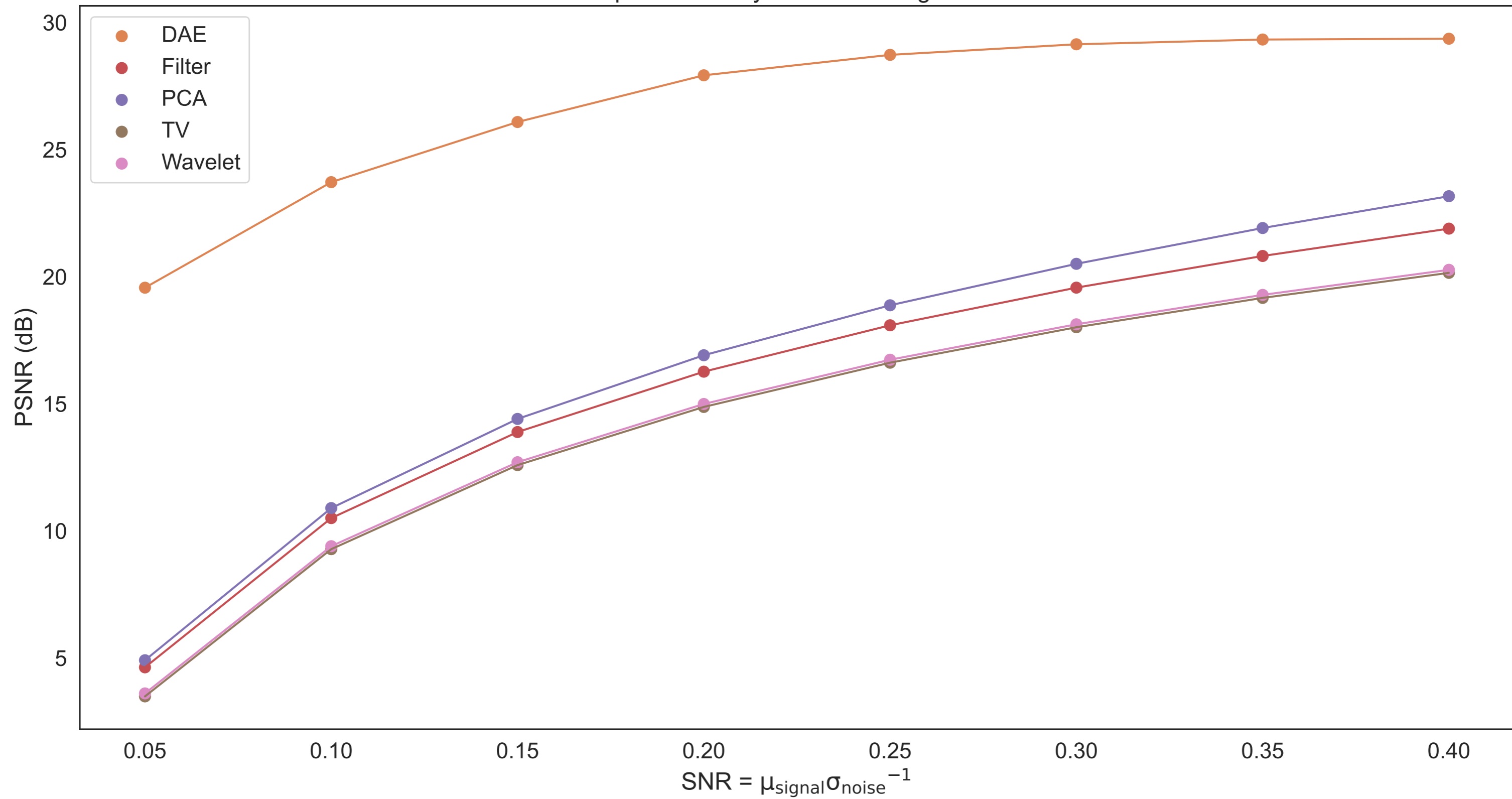

Supplement: Supplementary file 2 — LaTeX Supplementary File [file 41467_2025_62602_MOESM2_ESM.zip › SI_Figures/SI_Comp3.pdf]

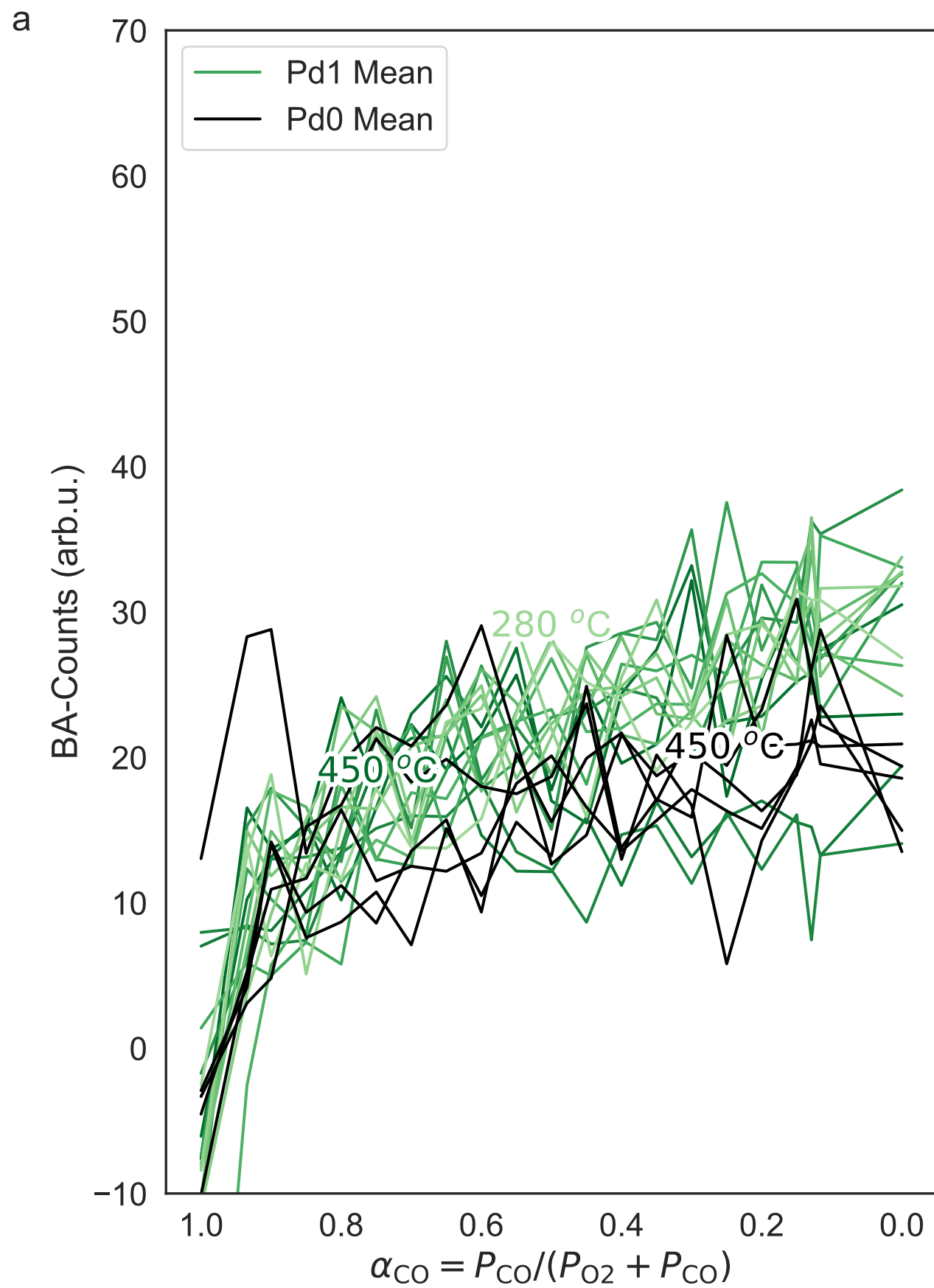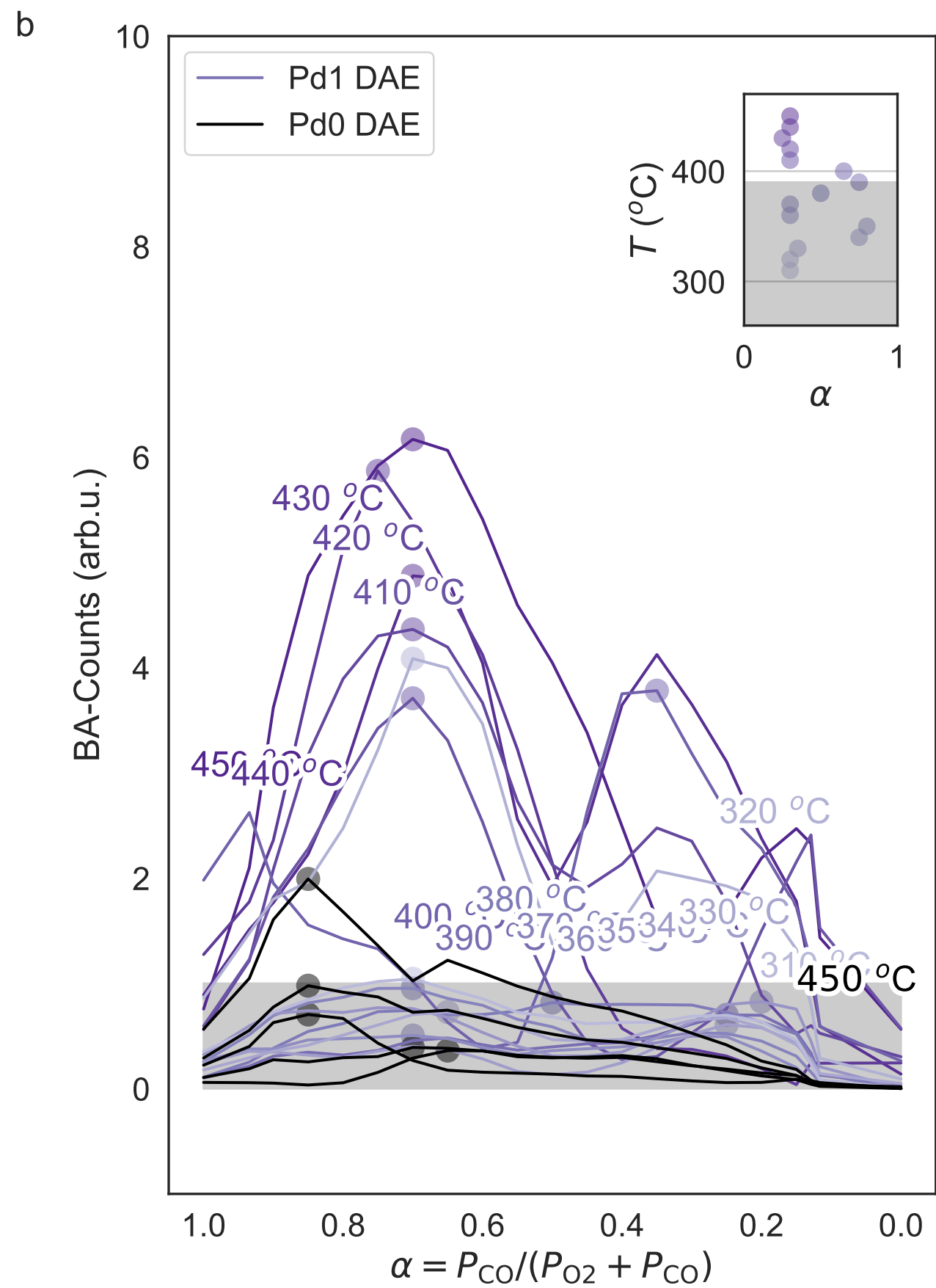

Supplement: Supplementary file 2 — LaTeX Supplementary File [file 41467_2025_62602_MOESM2_ESM.zip › SI_Figures/SI_FullPd1.pdf]

# Pd1000 Chip B QMS Output

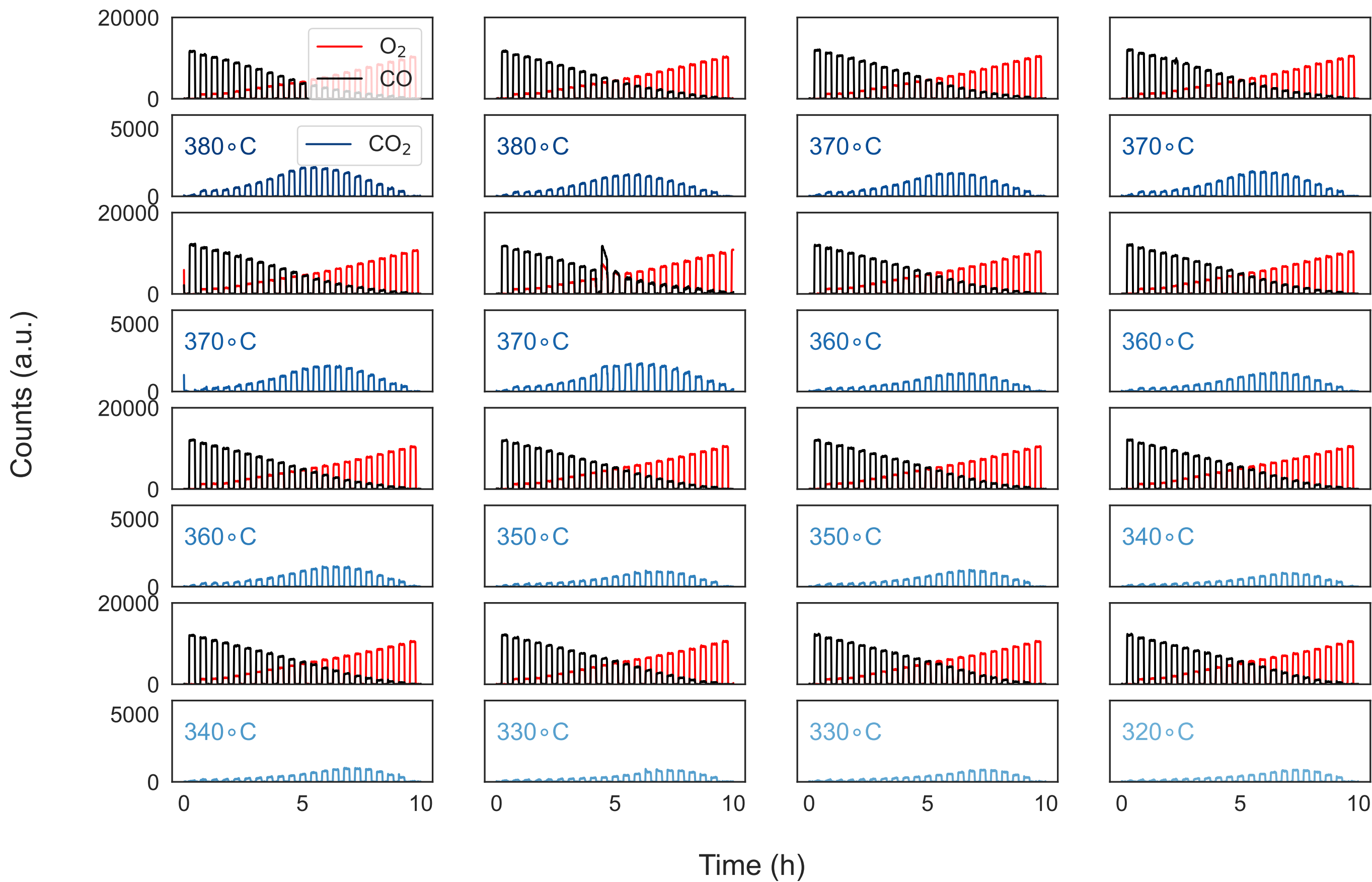

Supplement: Supplementary file 2 — LaTeX Supplementary File [file 41467_2025_62602_MOESM2_ESM.zip › SI_Figures/QMSCombined1000_ChipB.pdf]

Training loss curve

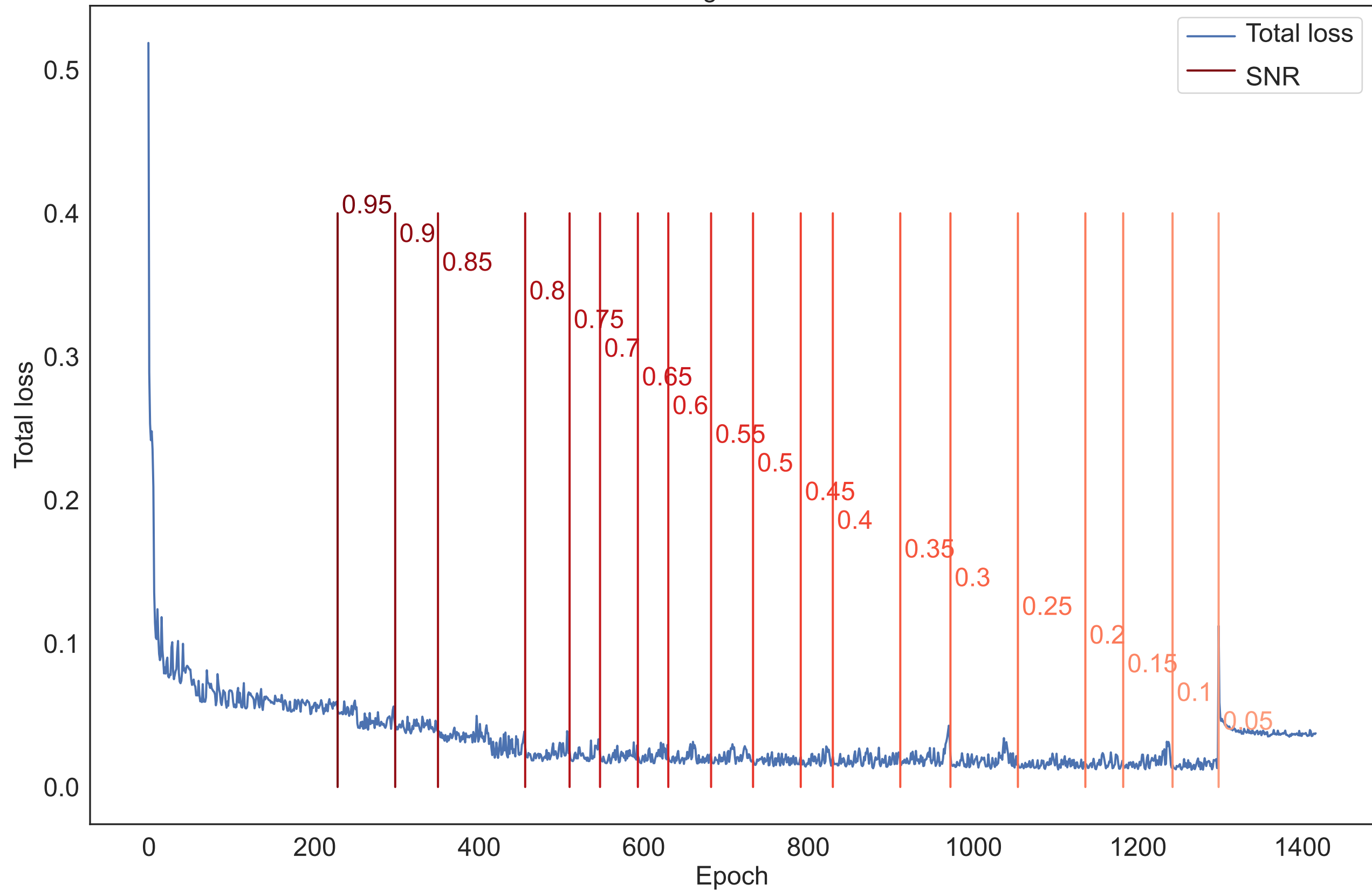

Supplement: Supplementary file 2 — LaTeX Supplementary File [file 41467_2025_62602_MOESM2_ESM.zip › SI_Figures/SI_LossCurve.pdf]

Step 1

Pump to  $P_{\text{out}} = 10^{-10}$  mbar

Heat to  $T = 280^\circ\text{C}$  mbar

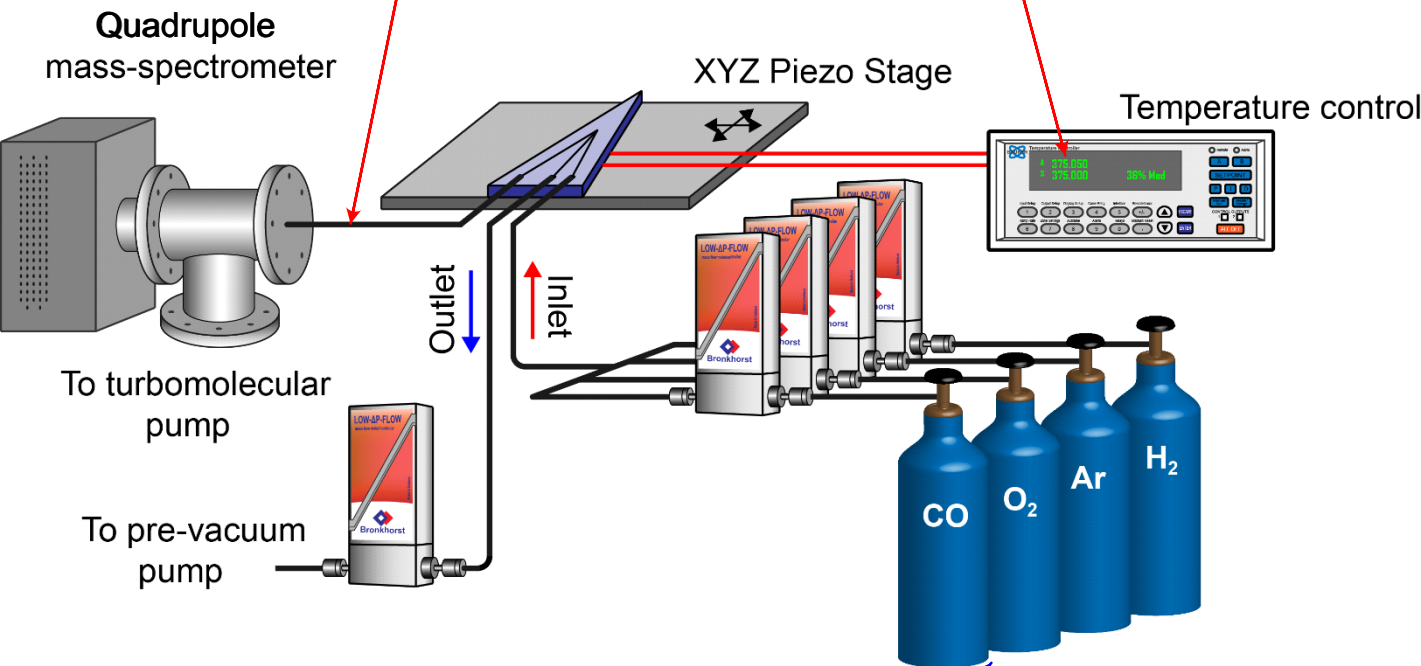

Step 2

20 Cycles of CO (10%)  
and O<sub>2</sub> (15%)

Supplement: Supplementary file 2 — LaTeX Supplementary File [file 41467_2025_62602_MOESM2_ESM.zip › SI_Figures/SI_pretreatment.pdf]

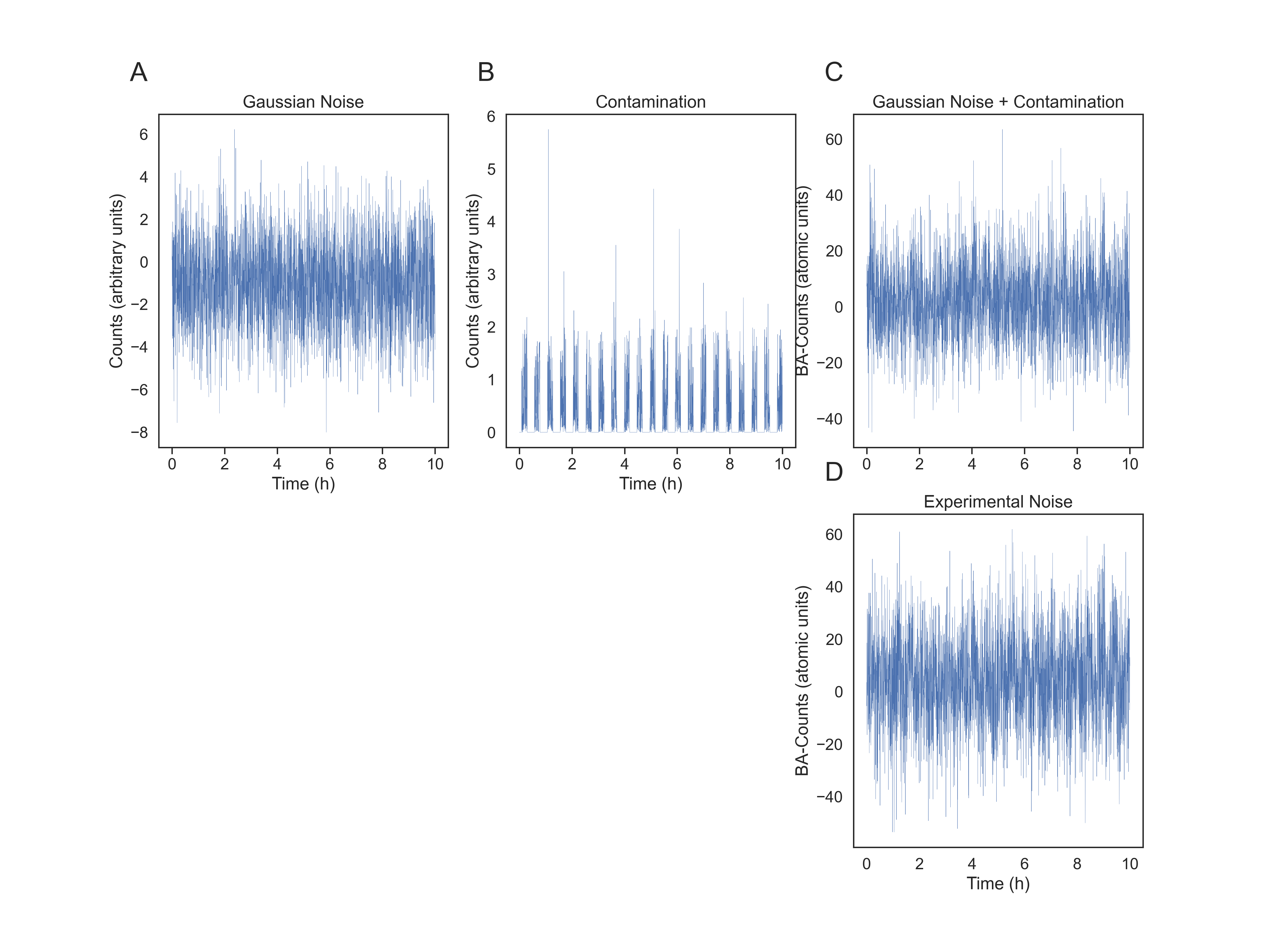

Supplement: Supplementary file 2 — LaTeX Supplementary File [file 41467_2025_62602_MOESM2_ESM.zip › SI_Figures/SI_noise_components.png]

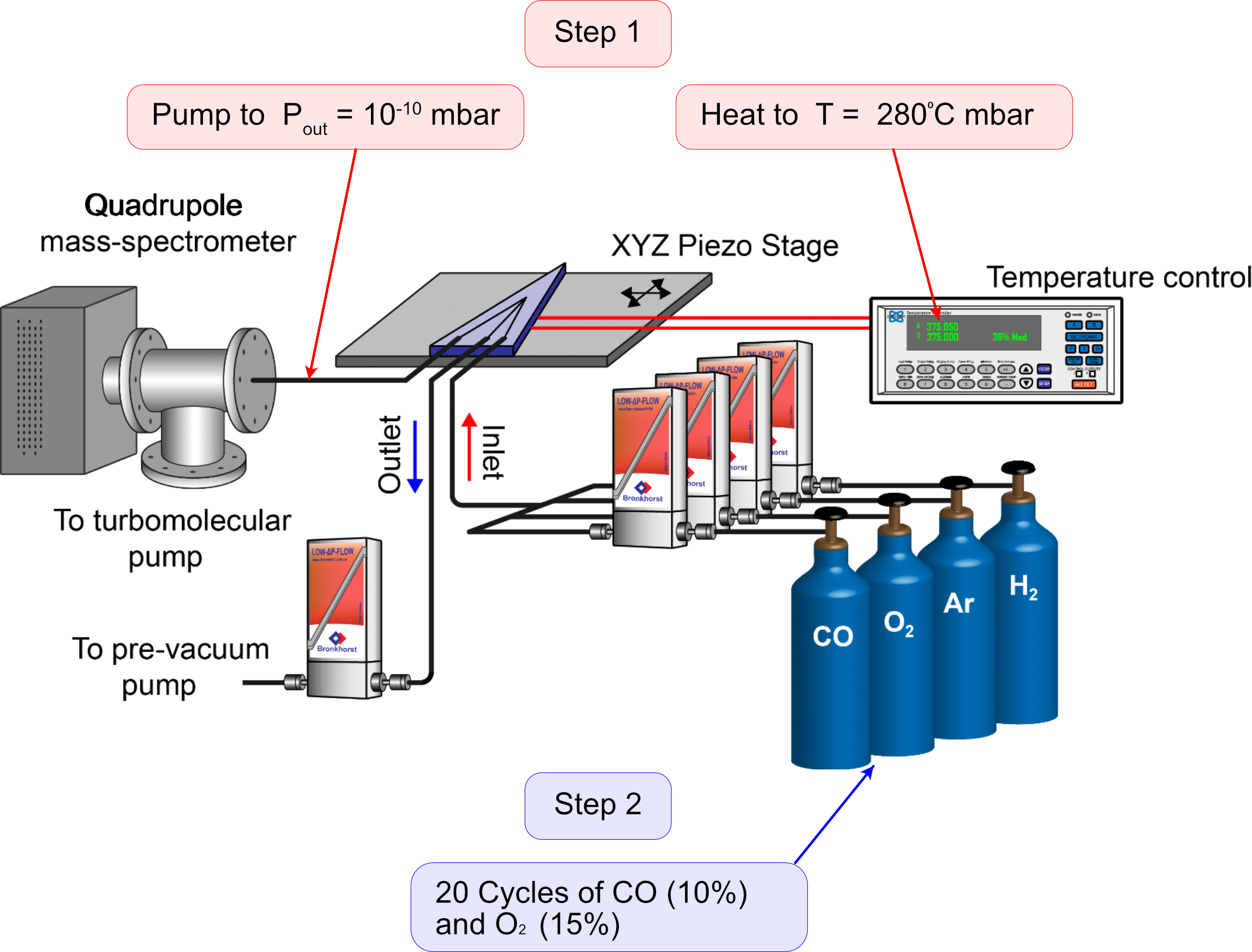

Supplement: Supplementary file 2 — LaTeX Supplementary File [file 41467_2025_62602_MOESM2_ESM.zip › SI_Figures/SI_pretreatment.png]

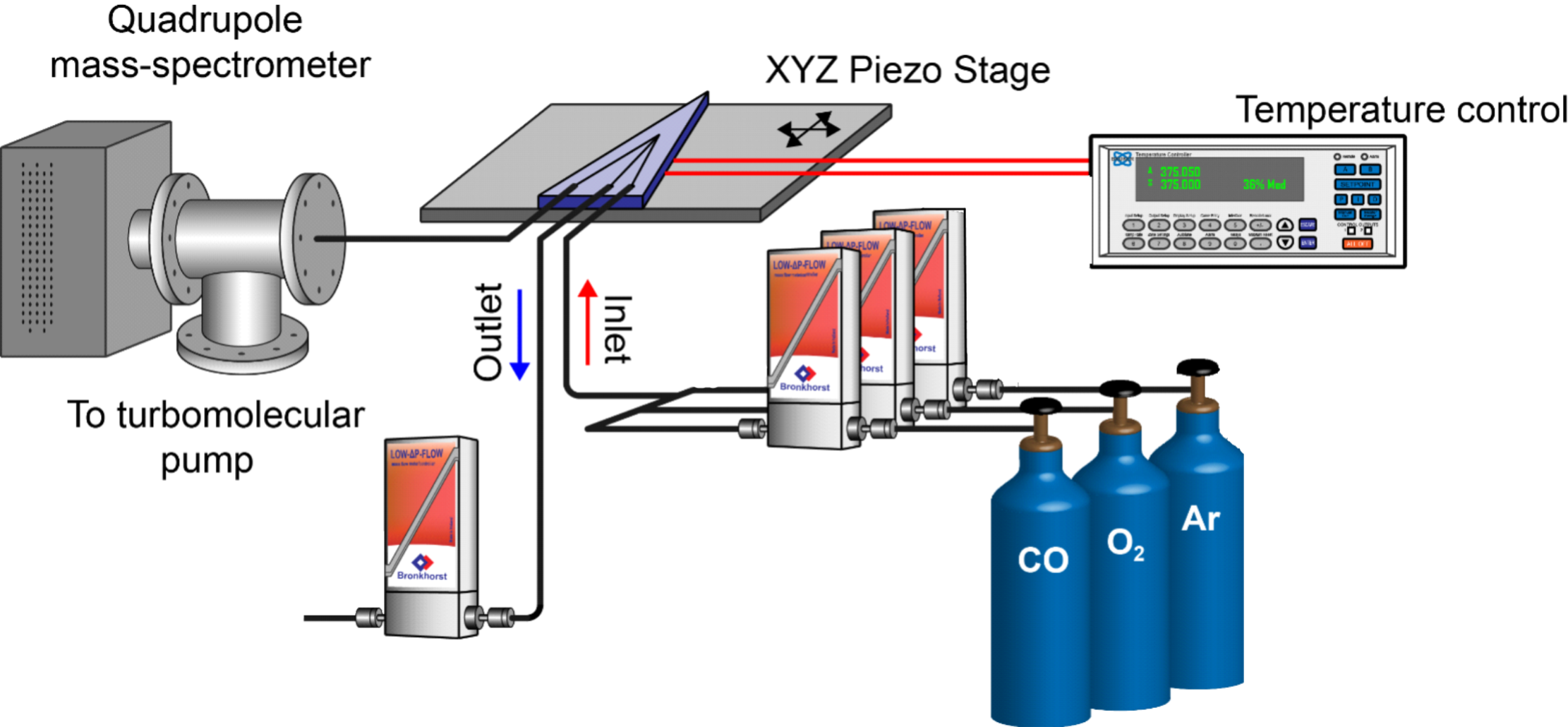

Supplement: Supplementary file 2 — LaTeX Supplementary File [file 41467_2025_62602_MOESM2_ESM.zip › SI_Figures/SI_pretreatment_v3.png]

Step 1

Pump to  $P_{\text{out}} = 10^{-10}$  mbar

Heat to  $T = 280^\circ\text{C}$  mbar

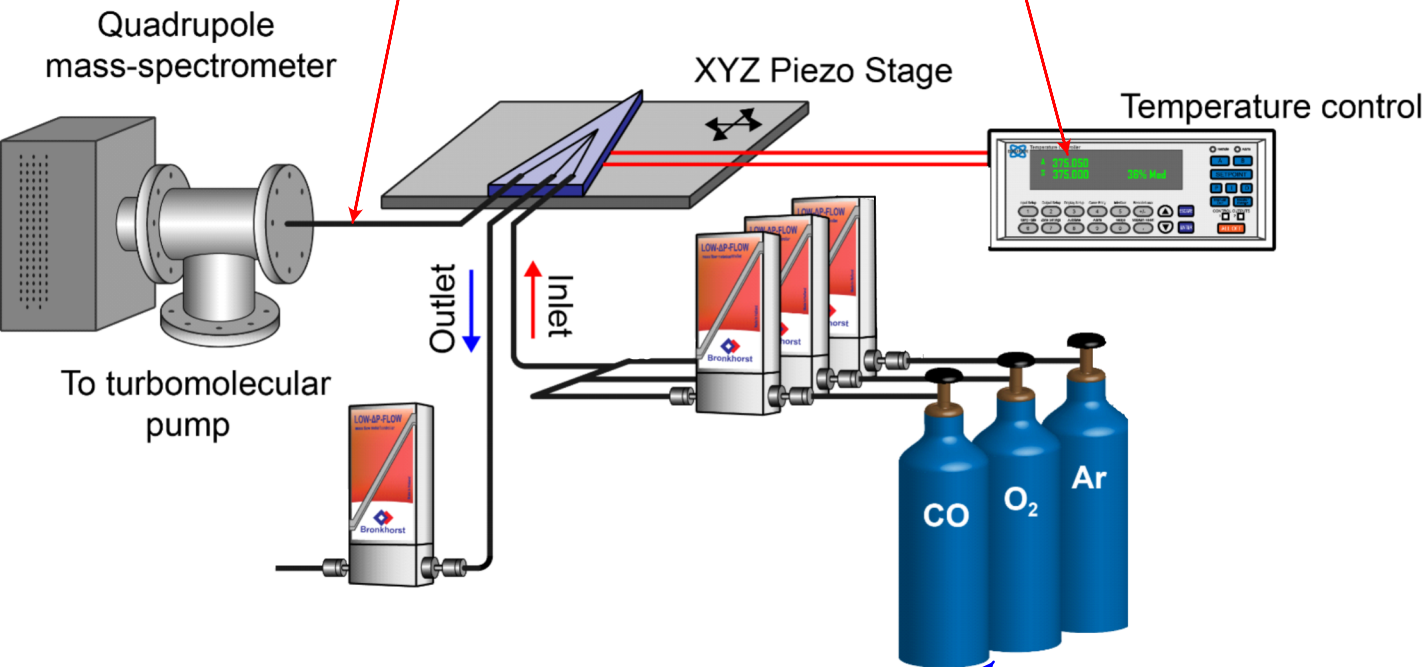

Step 2

20 Cycles of CO (10%)  
and O<sub>2</sub> (15%)

Supplement: Supplementary file 2 — LaTeX Supplementary File [file 41467_2025_62602_MOESM2_ESM.zip › SI_Figures/SI_pretreatment_v2.pdf]

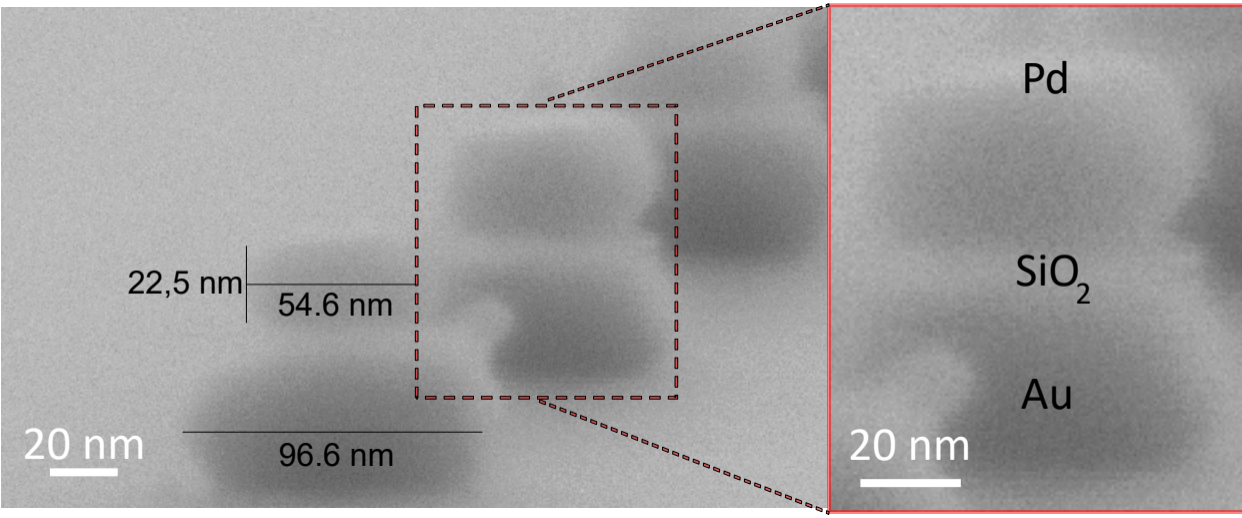

Supplement: Supplementary file 2 — LaTeX Supplementary File [file 41467_2025_62602_MOESM2_ESM.zip › SI_Figures/SI_SEMNP.pdf]

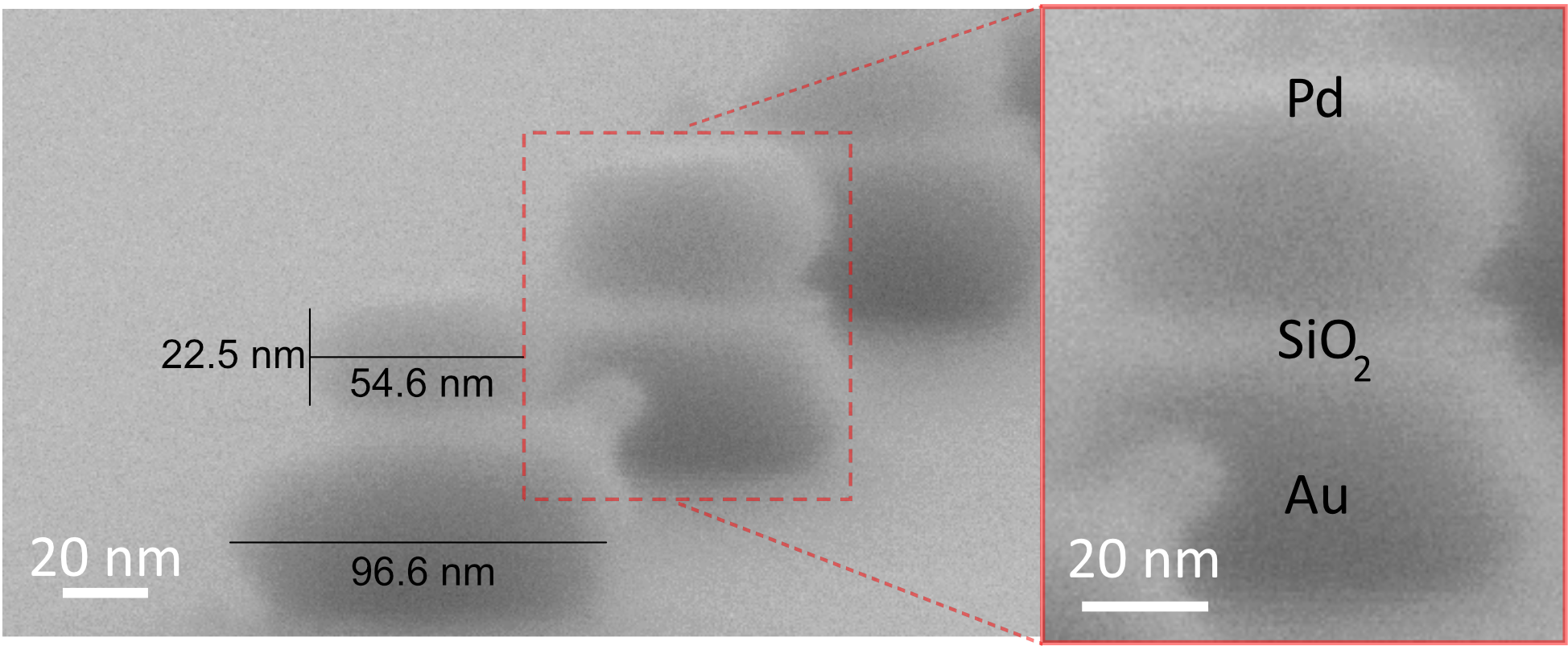

Supplement: Supplementary file 2 — LaTeX Supplementary File [file 41467_2025_62602_MOESM2_ESM.zip › SI_Figures/SI_SEMNP.png]

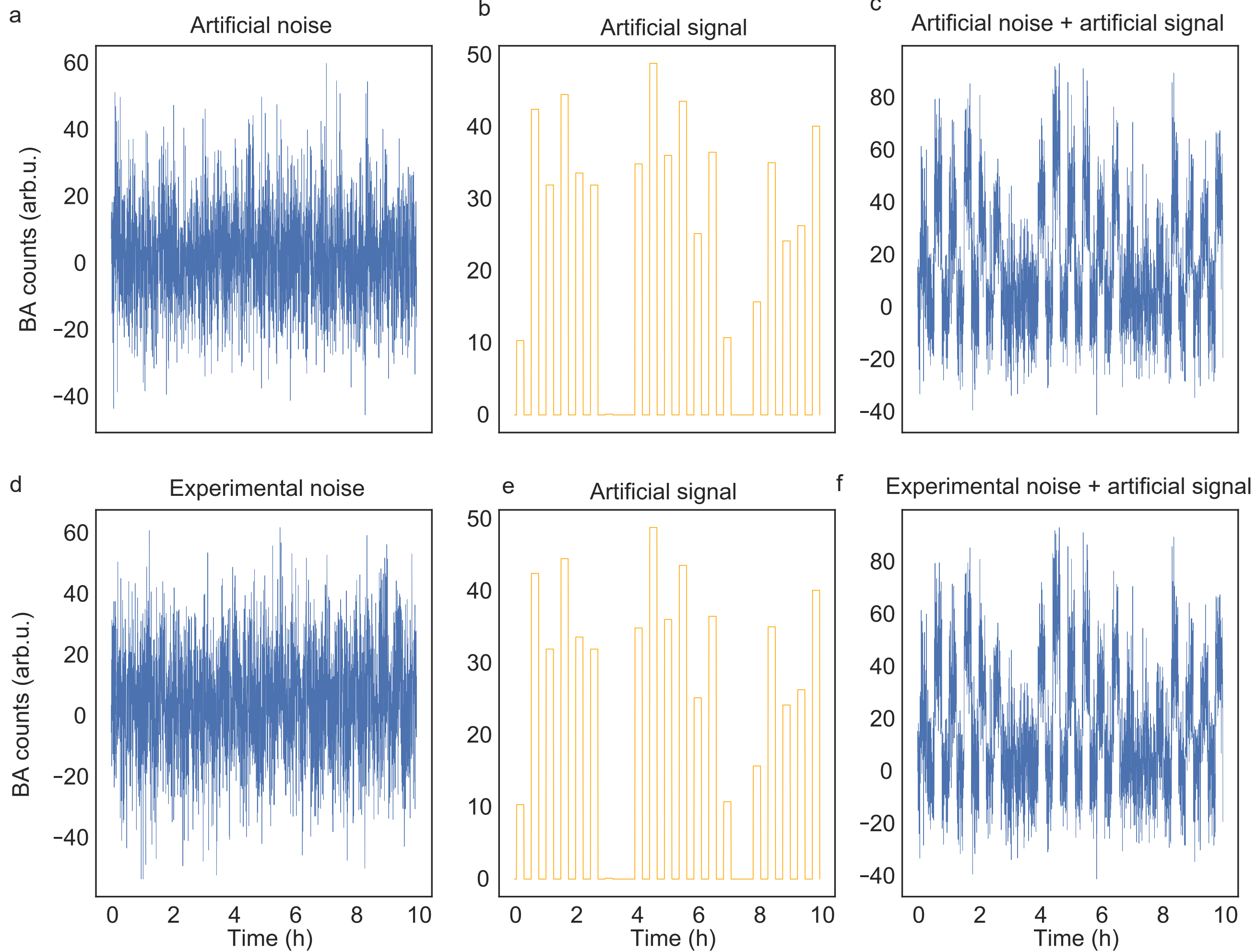

Supplement: Supplementary file 2 — LaTeX Supplementary File [file 41467_2025_62602_MOESM2_ESM.zip › SI_Figures/SI_signal_components.pdf]

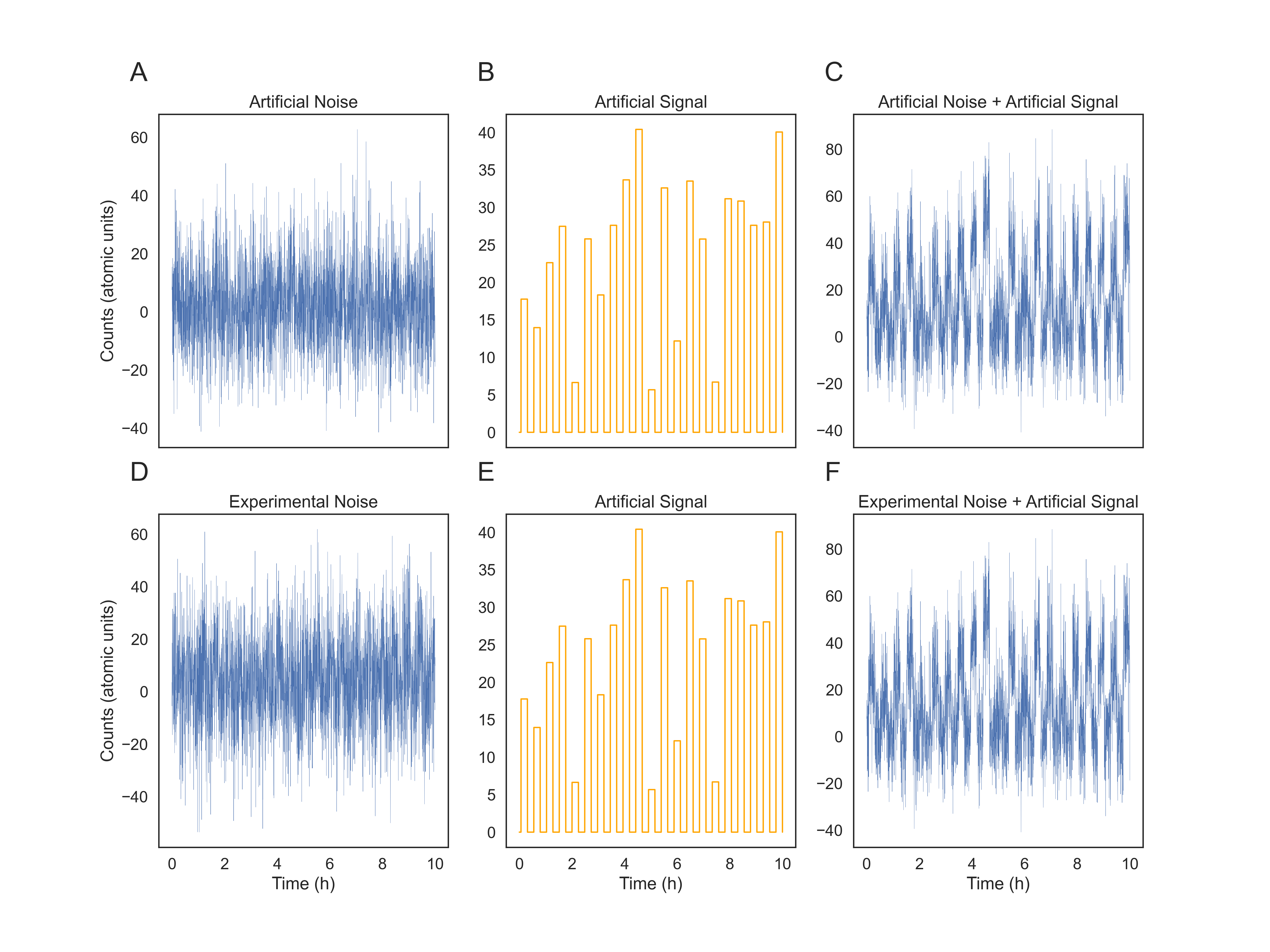

Supplement: Supplementary file 2 — LaTeX Supplementary File [file 41467_2025_62602_MOESM2_ESM.zip › SI_Figures/SI_signal_components.png]

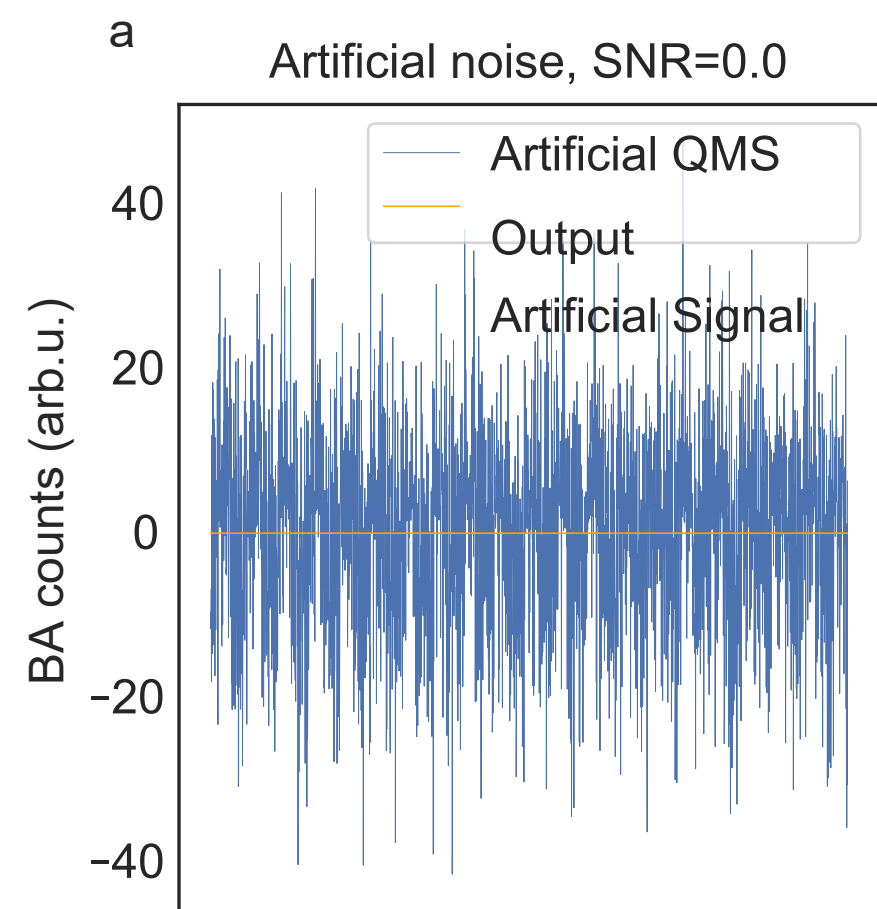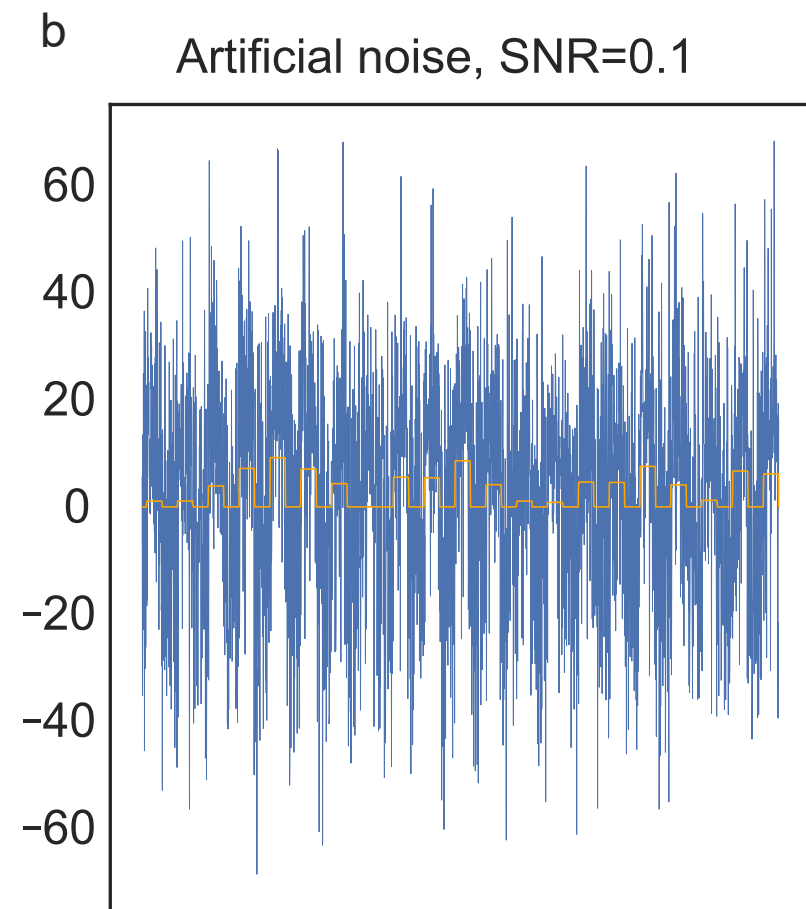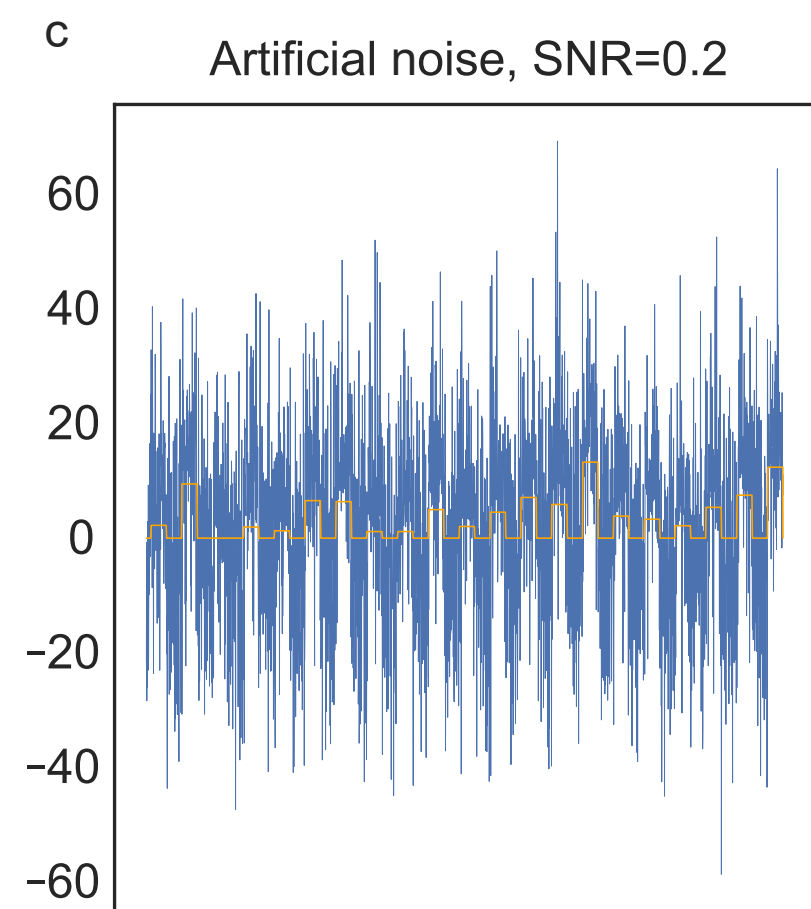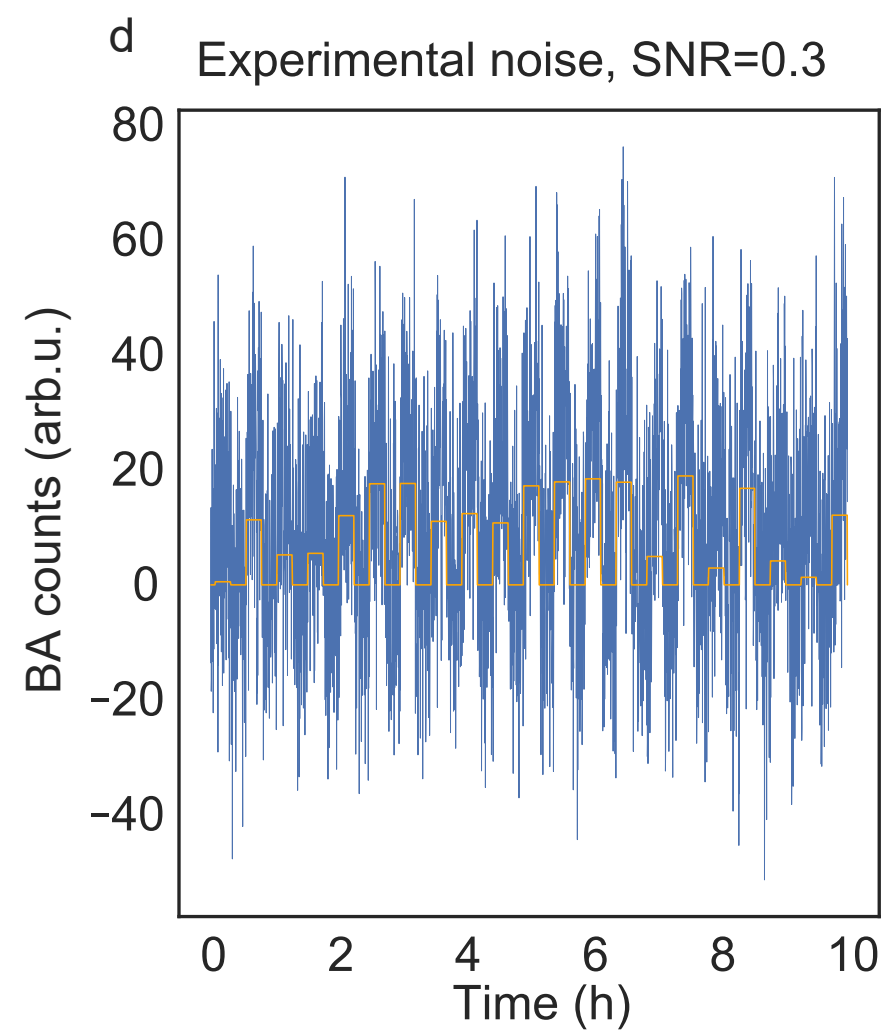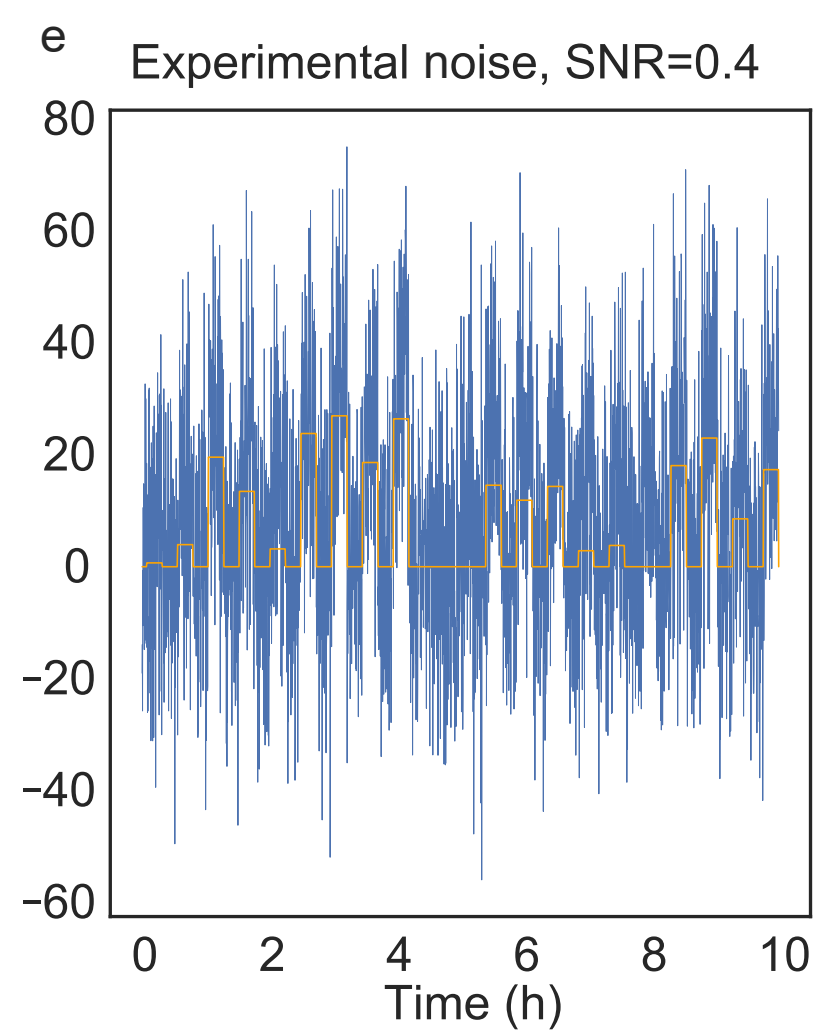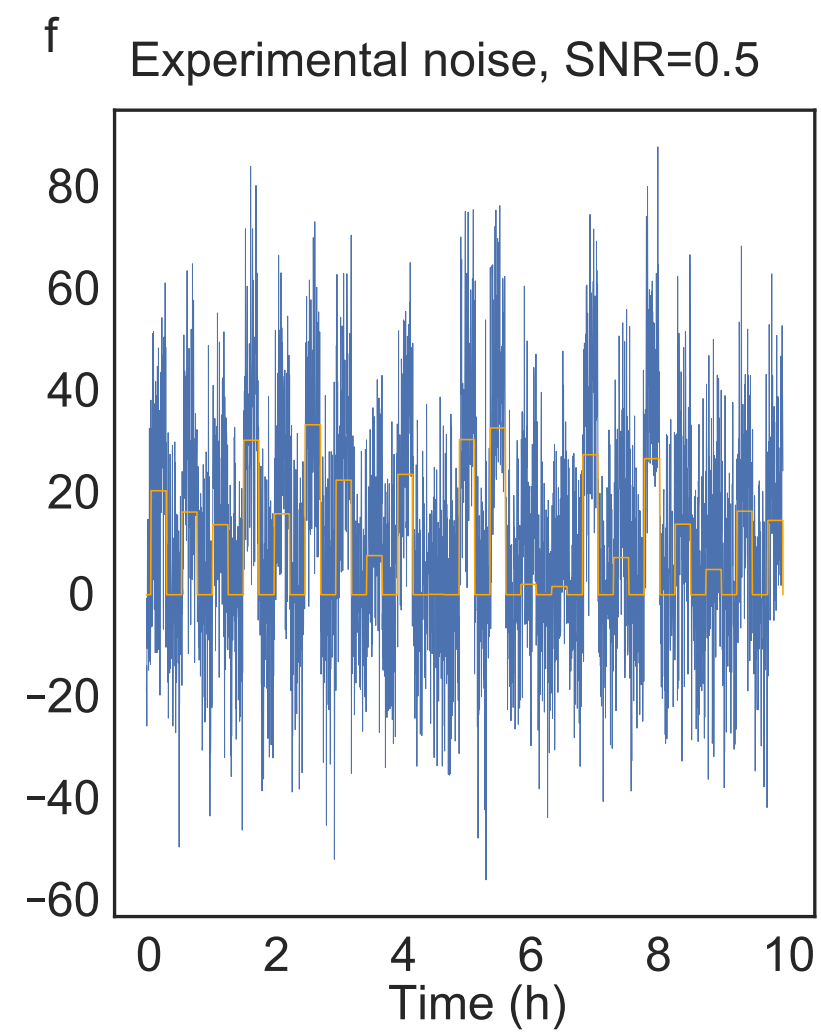

Supplement: Supplementary file 2 — LaTeX Supplementary File [file 41467_2025_62602_MOESM2_ESM.zip › SI_Figures/SI_training_data.pdf]

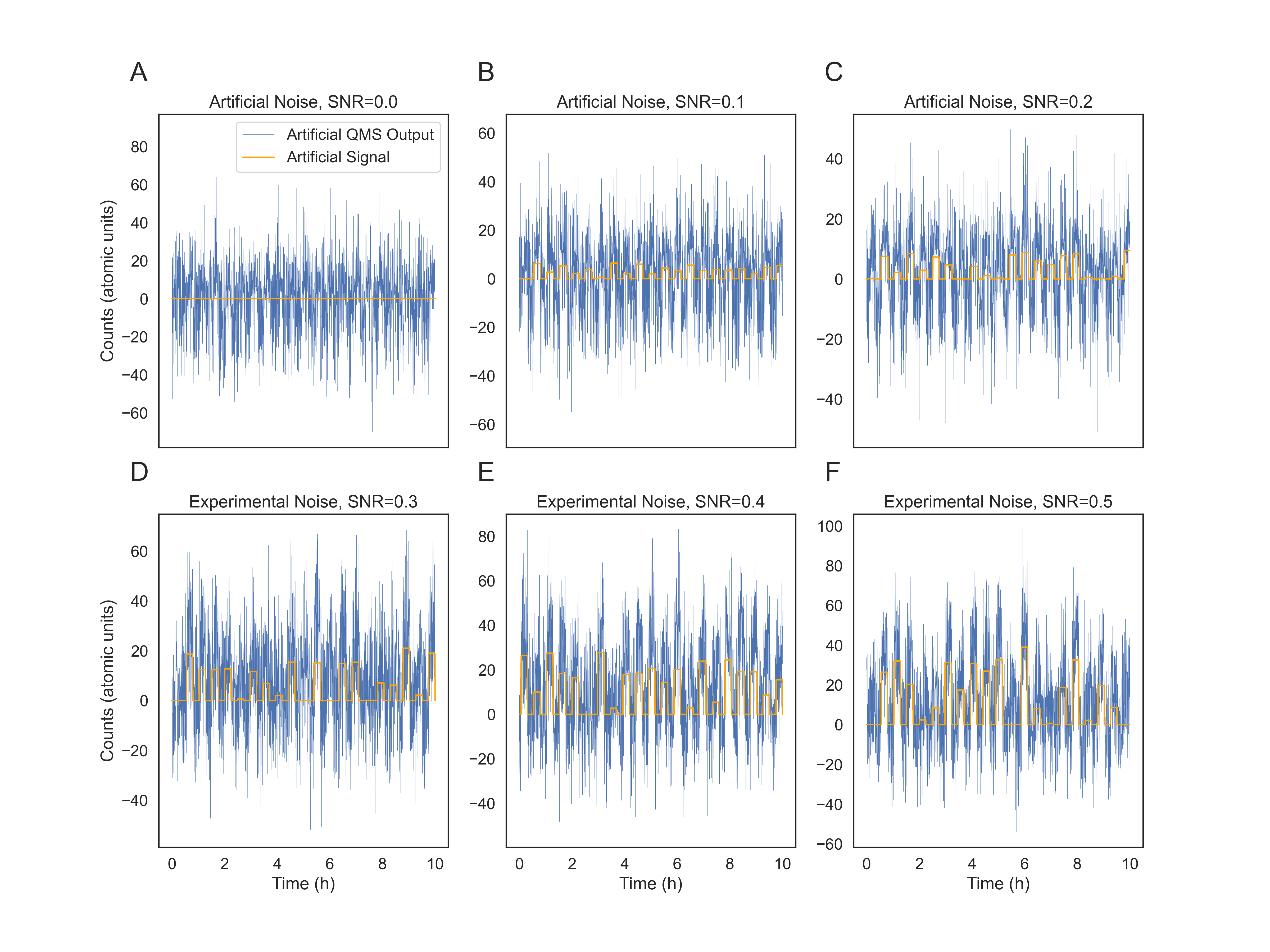

Supplement: Supplementary file 2 — LaTeX Supplementary File [file 41467_2025_62602_MOESM2_ESM.zip › SI_Figures/SI_training_data.png]

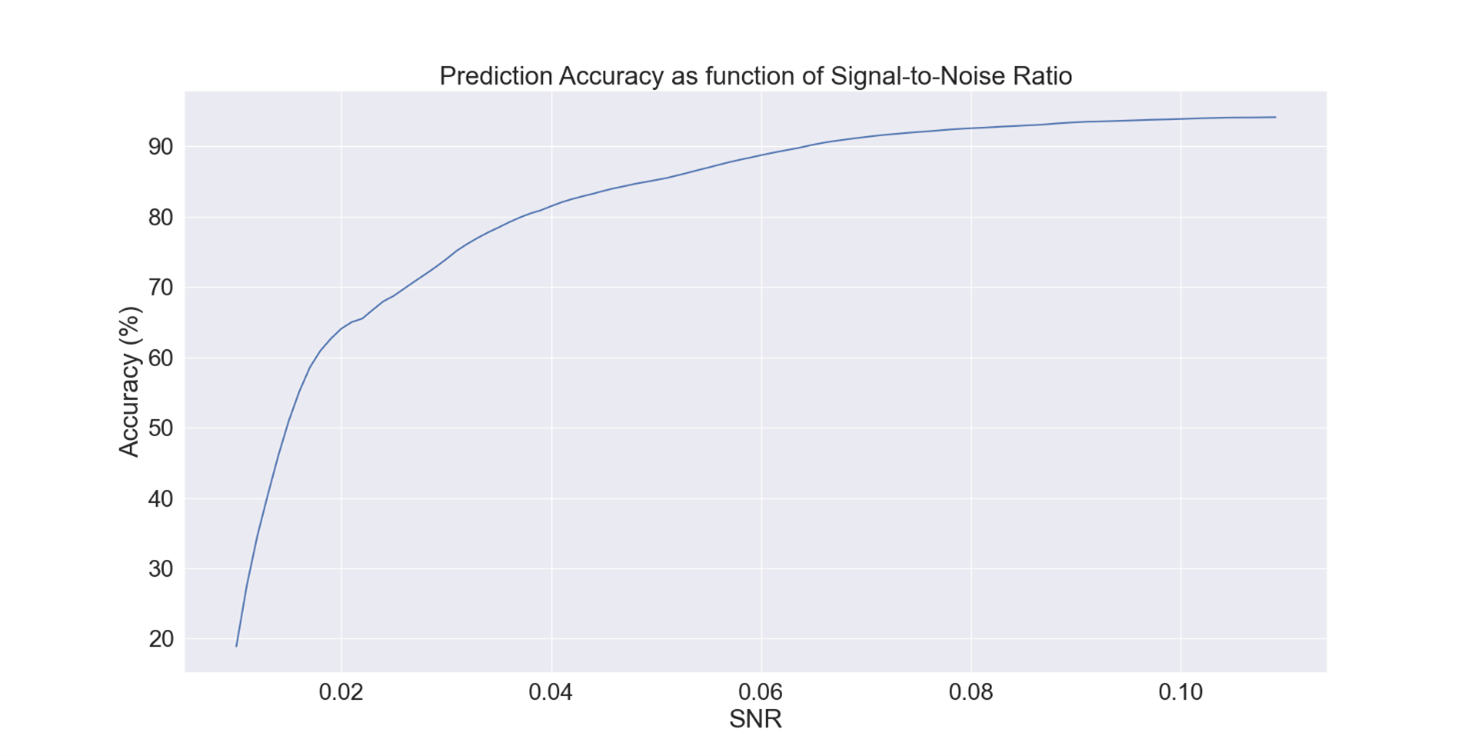

Supplement: Supplementary file 2 — LaTeX Supplementary File [file 41467_2025_62602_MOESM2_ESM.zip › SI_Figures/SNRAcc.png]

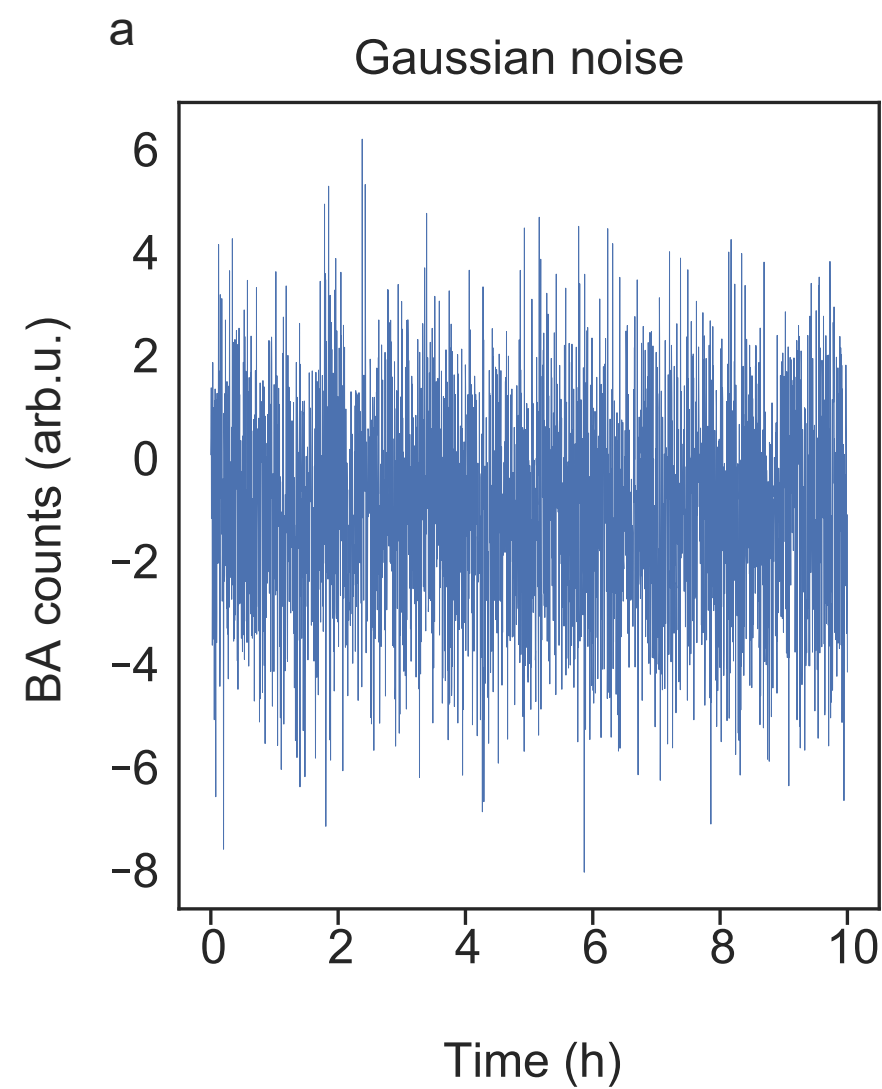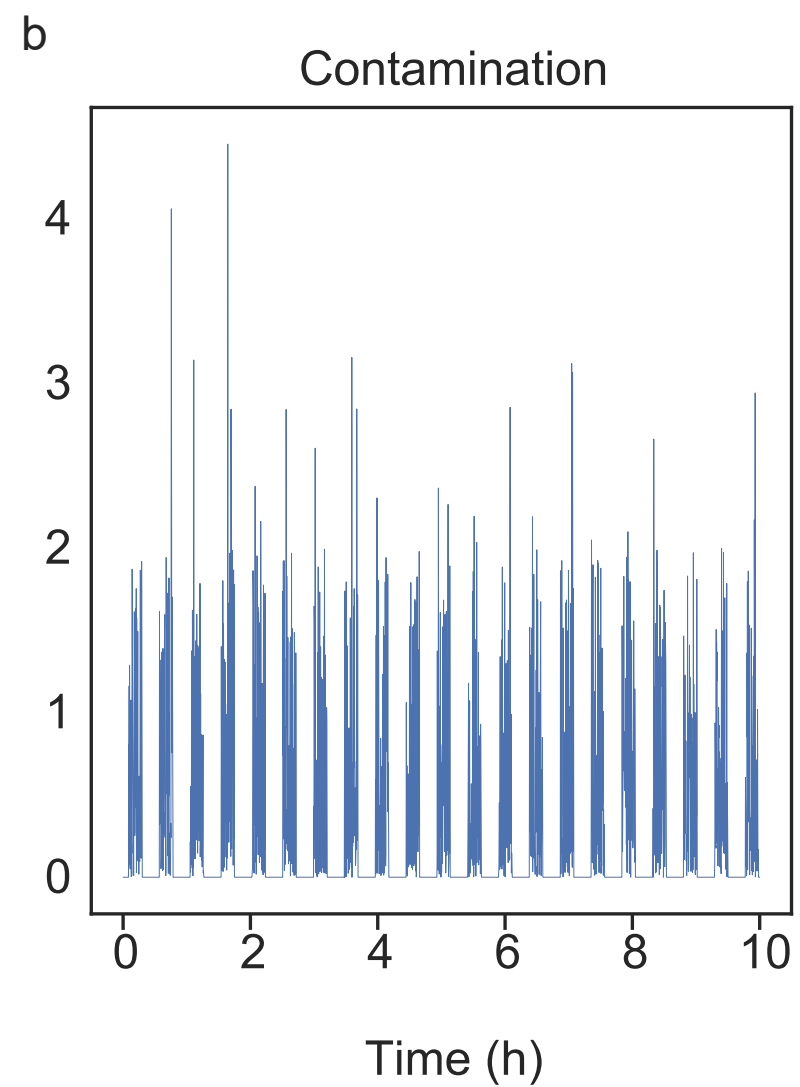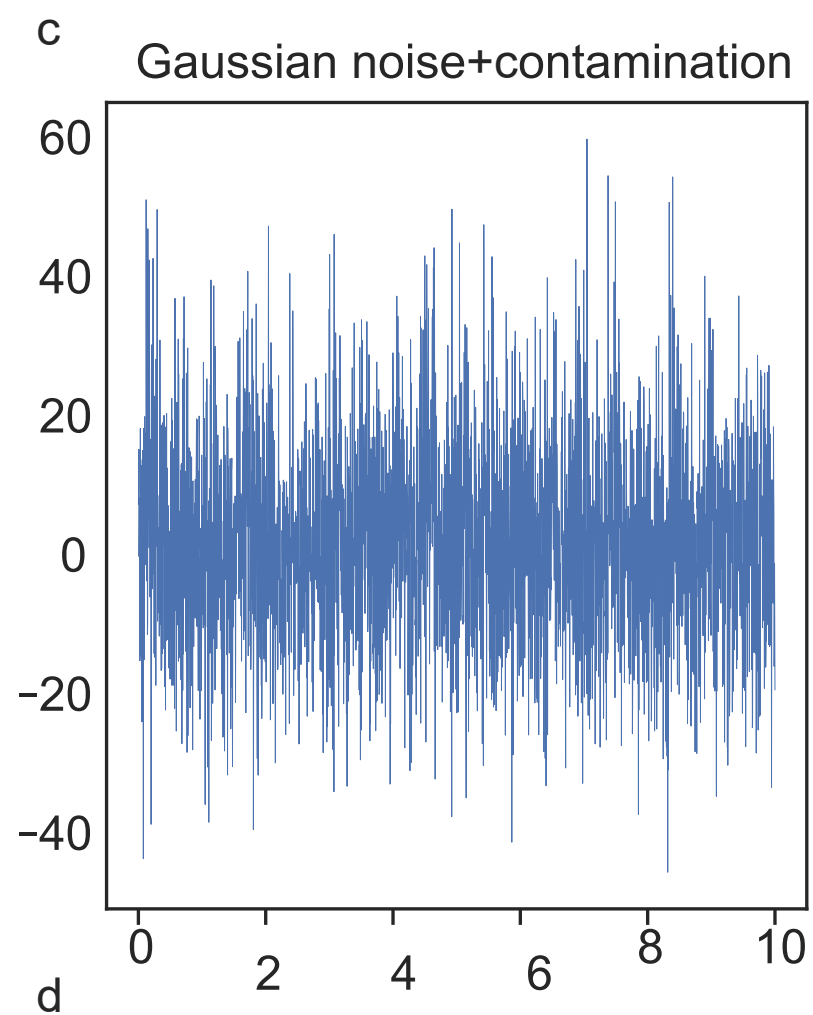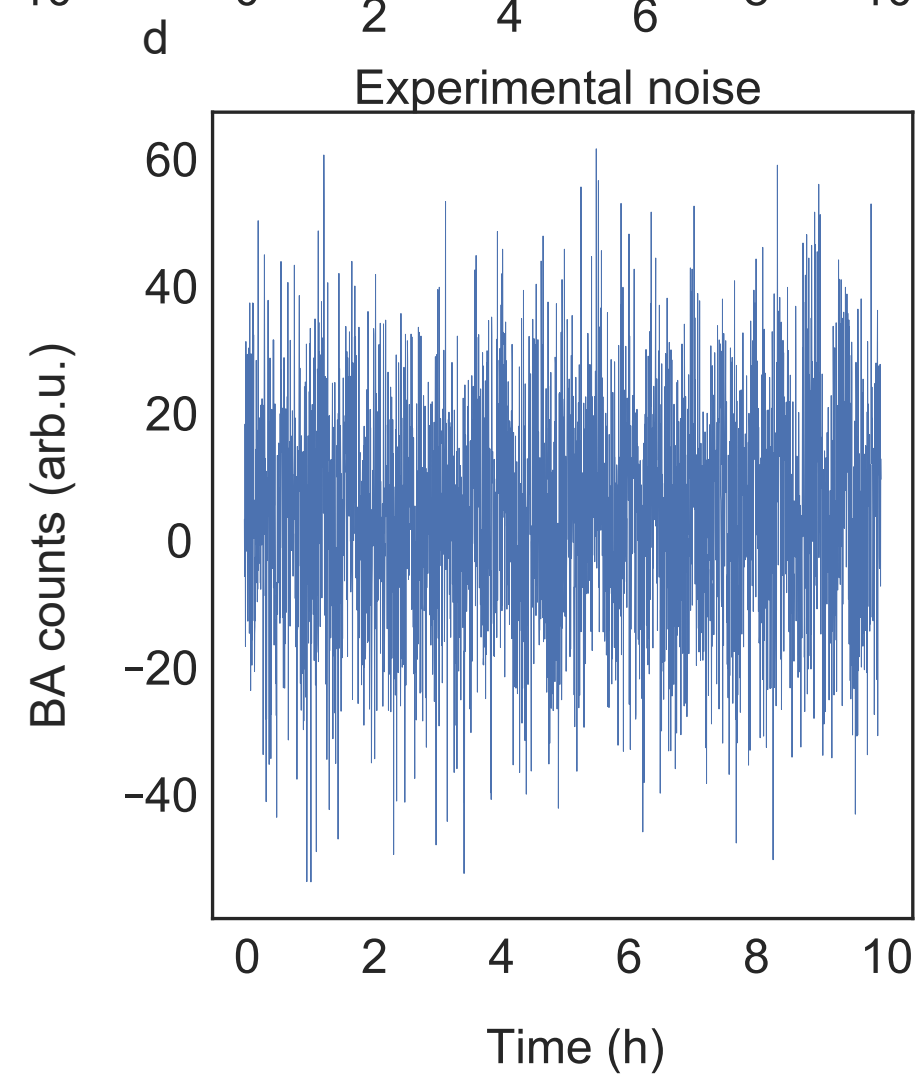

Supplement: Supplementary file 2 — LaTeX Supplementary File [file 41467_2025_62602_MOESM2_ESM.zip › SI_Figures/SI_noise_components.pdf]

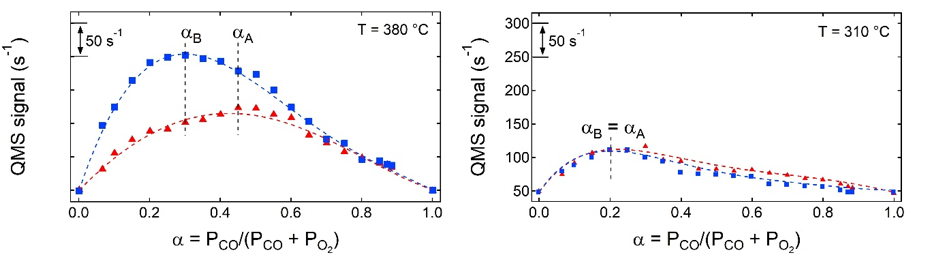

Supplement: Supplementary file 2 — LaTeX Supplementary File [file 41467_2025_62602_MOESM2_ESM.zip › SI_Figures/AlphaAB38010.png]

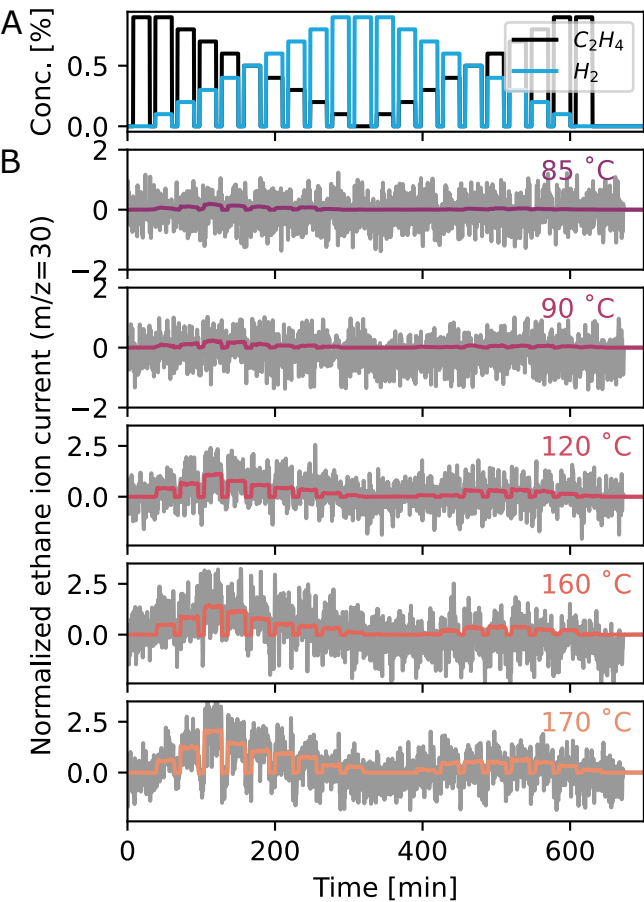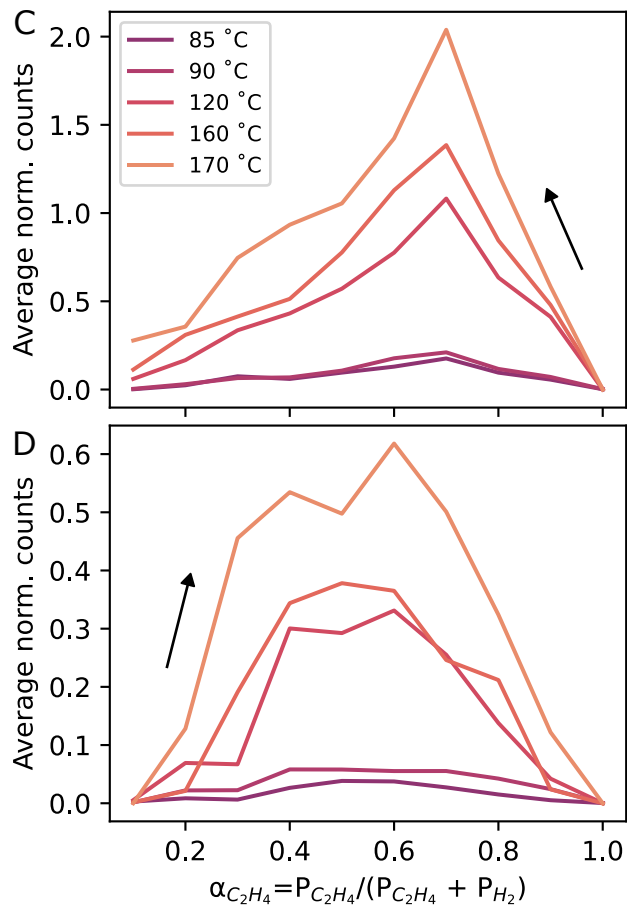

Supplement: Supplementary file 2 — LaTeX Supplementary File [file 41467_2025_62602_MOESM2_ESM.zip › SI_Figures/ethyelene_figure_v1.pdf]

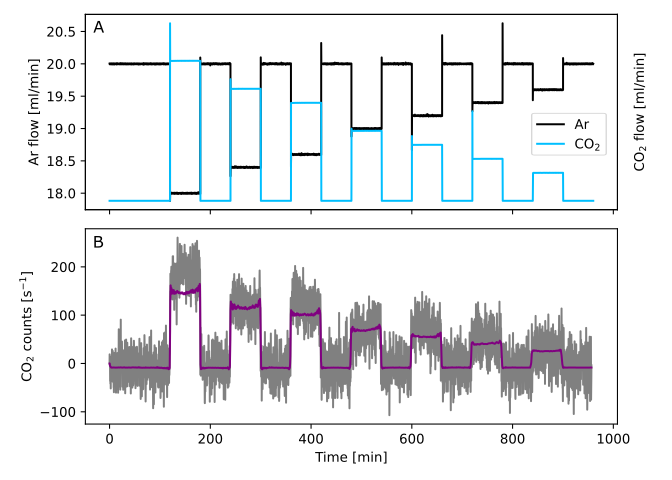

Supplement: Supplementary file 2 — LaTeX Supplementary File [file 41467_2025_62602_MOESM2_ESM.zip › SI_Figures/SI_CO2_experiment_figure.png]

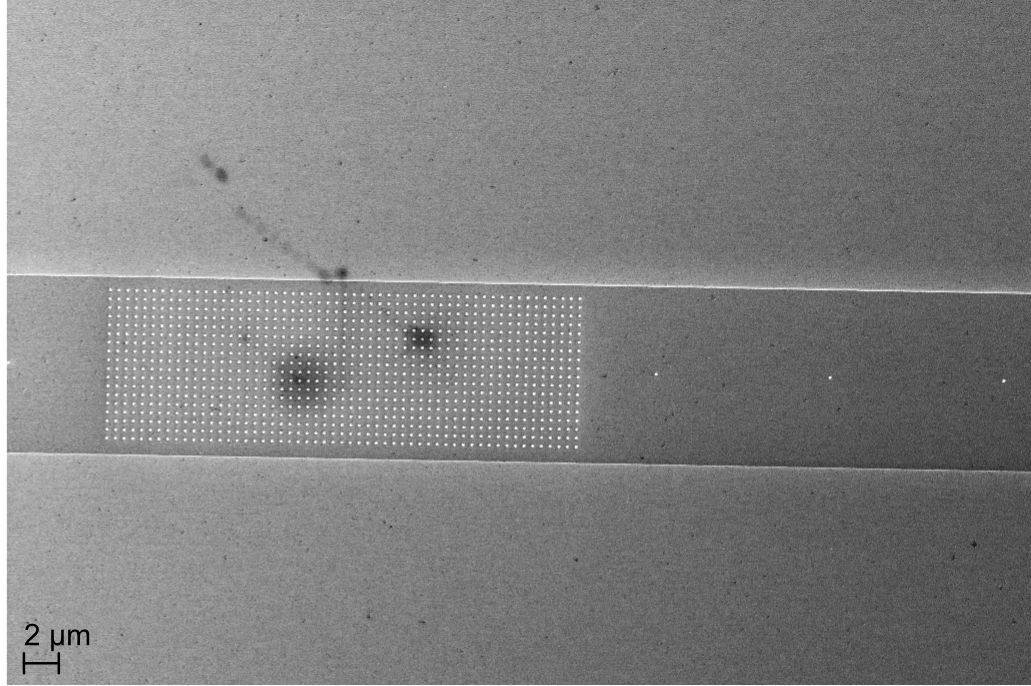

Supplement: Supplementary file 2 — LaTeX Supplementary File [file 41467_2025_62602_MOESM2_ESM.zip › SI_Figures/chipA_01.png]

Training loss curve

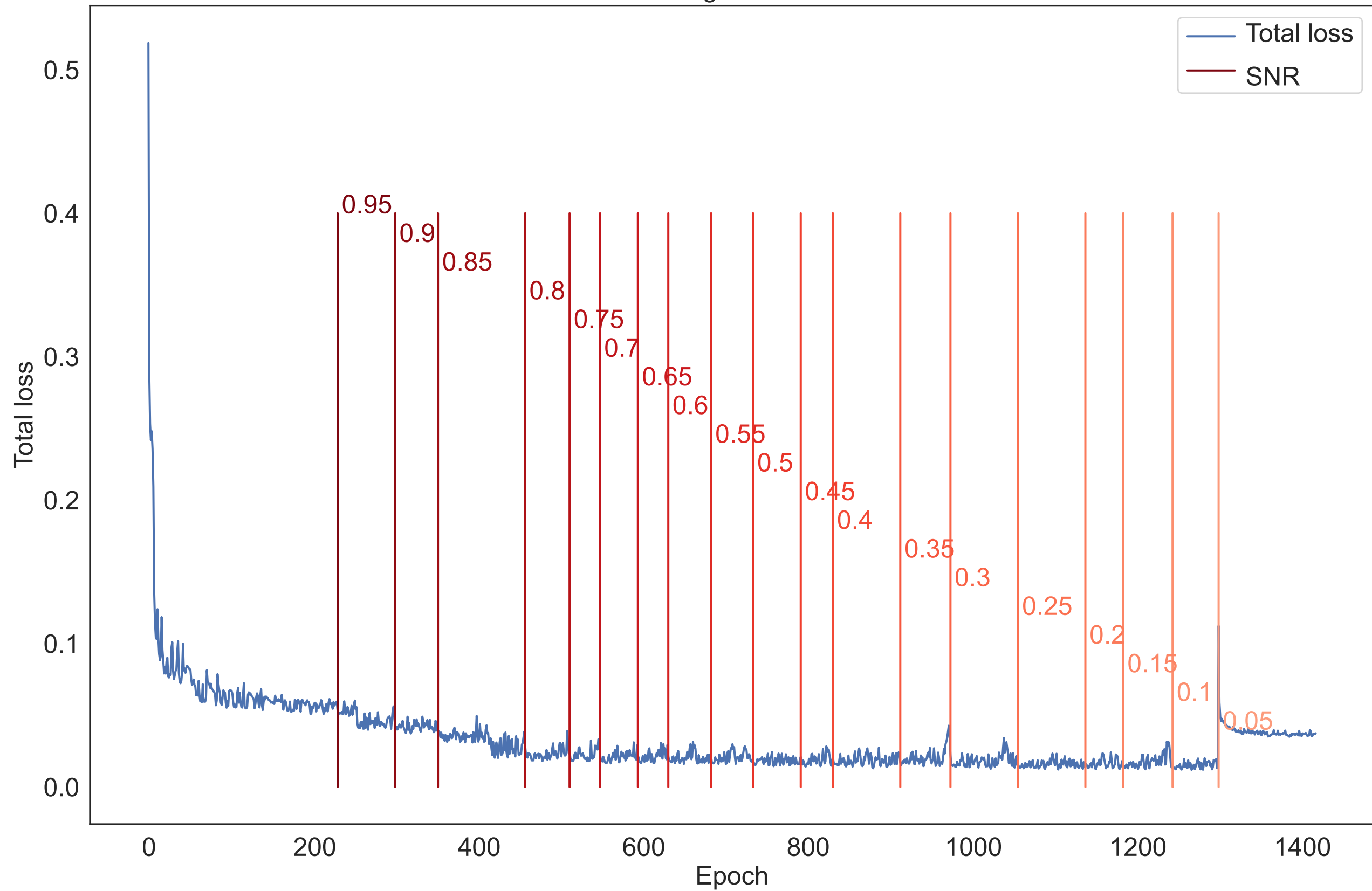

Supplement: Supplementary file 2 — LaTeX Supplementary File [file 41467_2025_62602_MOESM2_ESM.zip › SI_Figures/SI_LossCurve-1.pdf]

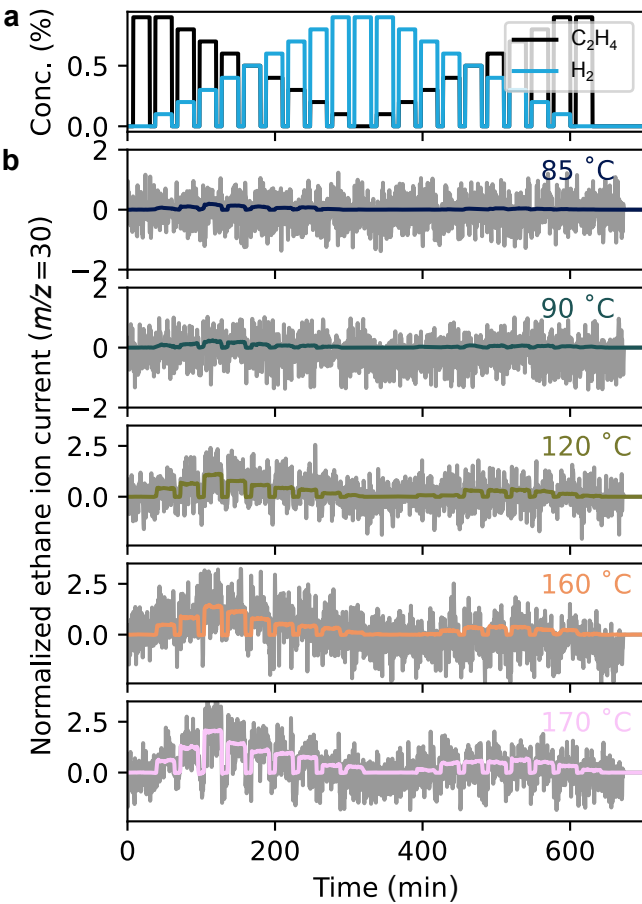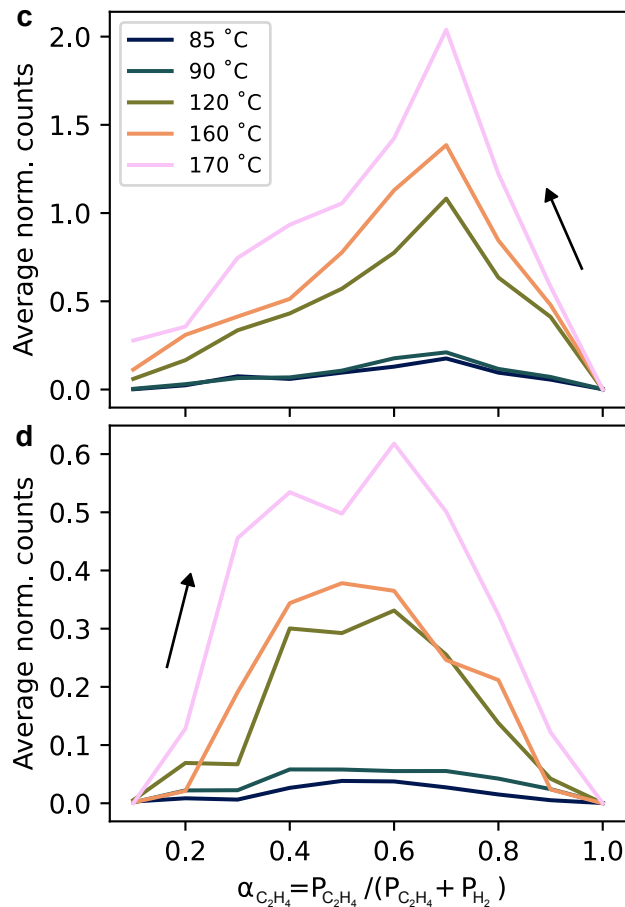

Supplement: Supplementary file 2 — LaTeX Supplementary File [file 41467_2025_62602_MOESM2_ESM.zip › SI_Figures/ethylene_figure_v1_revised.pdf]

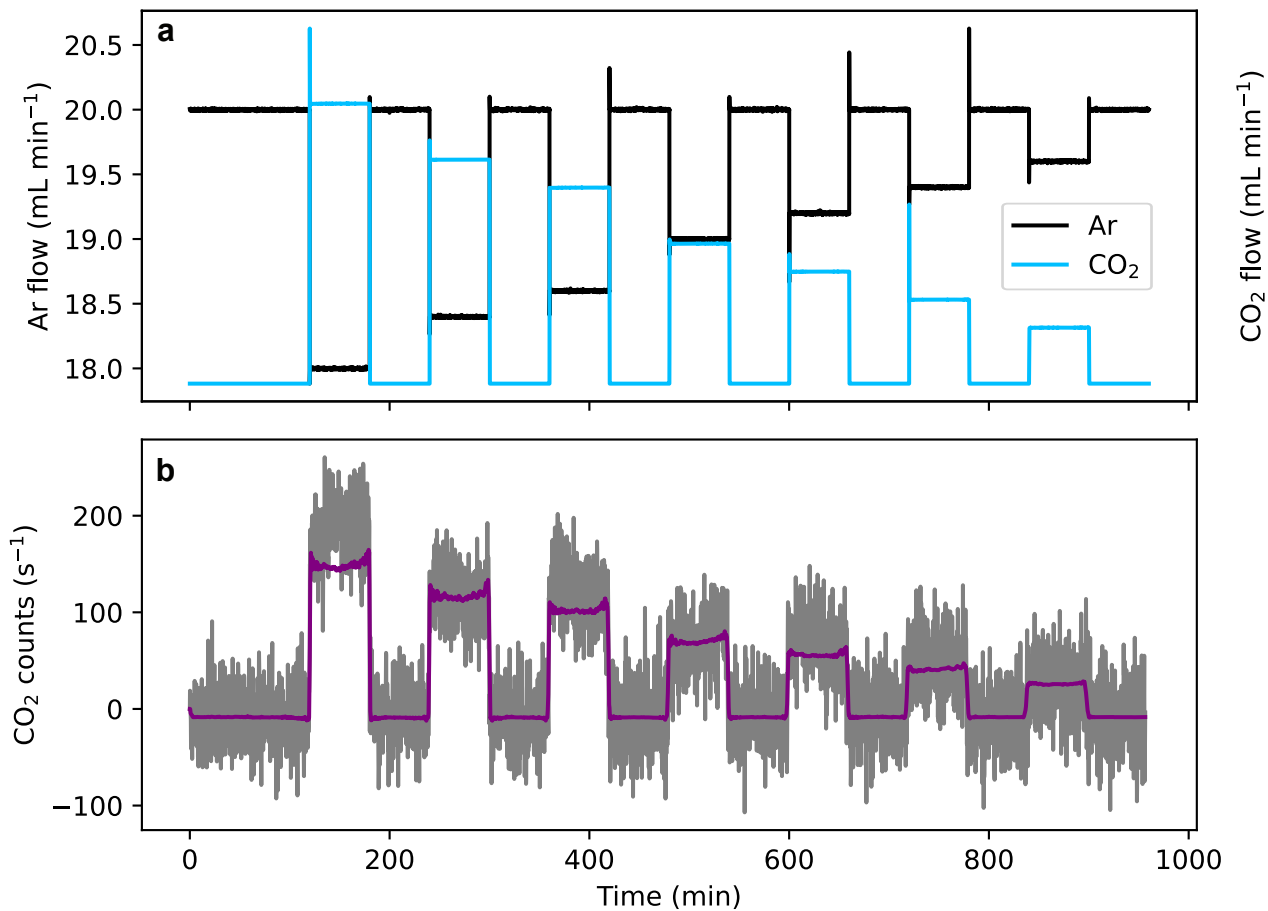

Supplement: Supplementary file 2 — LaTeX Supplementary File [file 41467_2025_62602_MOESM2_ESM.zip › SI_Figures/SI_CO2_revised.pdf]
